# Transcriptomic response of breast cancer cells to anacardic acid

**DOI:** 10.1038/s41598-018-26429-x

**Published:** 2018-05-23

**Authors:** David J. Schultz, Abirami Krishna, Stephany L. Vittitow, Negin Alizadeh-Rad, Penn Muluhngwi, Eric C. Rouchka, Carolyn M. Klinge

**Affiliations:** 1 Department of Biology, University of Louisville, Louisville, KY USA; 2 Department of Biochemistry & Molecular Genetics, University of Louisville School of Medicine, Louisville, KY USA; 3 Bioinformatics and Biomedical Computing Laboratory, Department of Computer Engineering and Computer Science, Louisville, KY 40292 USA

## Abstract

Anacardic acid (AnAc), a potential dietary agent for preventing and treating breast cancer, inhibited the proliferation of estrogen receptor α (ERα) positive MCF-7 and MDA-MB-231 triple negative breast cancer cells. To characterize potential regulators of AnAc action, MCF-7 and MDA-MB-231 cells were treated for 6 h with purified AnAc 24:1n5 congener followed by next generation transcriptomic sequencing (RNA-seq) and network analysis. We reported that AnAc-differentially regulated miRNA transcriptomes in each cell line and now identify AnAc-regulated changes in mRNA and lncRNA transcript expression. In MCF-7 cells, 80 AnAc-responsive genes were identified, including lncRNA *MIR*2*2HG*. More AnAc-responsive genes (886) were identified in MDA-MB-231 cells. Only six genes were commonly altered by AnAc in both cell lines: *SCD, INSIG1*, and *TGM2* were decreased and *PDK4, GPR176*, and *ZBT20* were increased. Modeling of AnAc-induced gene changes suggests that AnAc inhibits monounsaturated fatty acid biosynthesis in both cell lines and increases endoplasmic reticulum stress in MDA-MB-231 cells. Since modeling of downregulated genes implicated NFκB in MCF-7, we confirmed that AnAc inhibited TNFα-induced NFκB reporter activity in MCF-7 cells. These data identify new targets and pathways that may account for AnAc’s anti-proliferative and pro-apoptotic activity.

## Introduction

A number of plants produce anacardic acid (AnAc) which is a mixture of 6-alkylbenzoic acid congeners^1^. Previously, we showed that a specific congener, AnAc 24:1n5, acts as a concentration-dependent mixed agonist/antagonist of estrogen receptor (ERα)-induced proliferation and transcription and inhibits ERα-estrogen response element (ERE) binding by interacting with the DNA binding domain (DBD), thus acting as a nuclear receptor alternate site modulator (NRAM)^2^. AnAc 24:1n5 also inhibited MDA-MB-231 triple negative breast cancer (TNBC) cell proliferation, although at a higher IC_50_ and via an unknown mechanism^2^. We reported that the expression of endogenous estrogen-regulated genes, *i.e., TFF1, CCND1*, and *CTSD*, was inhibited by AnAc 24:1n5 in breast cancer cell lines^2^. However, because AnAc affects multiple molecular targets (reviewed in^3^) and since we detected an ERα-independent inhibition of TNBC cell proliferation by AnAc 24:1n5, we suspect additional unknown molecular targets, independent of ERα, are altered by AnAc in these cells. Gene expression profiling is used in drug development to understand and predict the activity of novel therapeutic compounds in pre-clinical settings. Transcriptome analysis using bioinformatics tools gives an overview of biological processes and pathways affected by a ‘drug’; thus providing new insights about the potential cellular targets and mechanisms of action of that ‘drug’. Identification of such targets using RNA-seq would be beneficial in identifying AnAc-regulated pathways and targets in both luminal A breast cancer and in TNBC which primarily affects premenopausal women with a predominance in women of African and Hispanic ancestry^4,5^.

In previous work using RNA-seq analysis of AnAc-treated MCF-7 and MDA-MB-231 cells we identified 69 and 37 AnAc-regulated miRNAs, respectively^6^. MetaCore enrichment analysis revealed that no miRNAs were downregulated by AnAc in both cell lines while two miRNAs were increased by AnAc in both cell lines: miR-612 and miR-20b with the common gene ontology (GO) process “cellular response to inorganic substance”^6^.

The goal of the study reported here was to use RNA-seq to identify alterations in mRNA target transcript levels in the same representative ERα-positive and TNBC breast cancer cell lines after AnAc 24:1n5 treatment. AnAc up- or down- regulated divergent and common mRNA transcripts in MCF-7 and MDA-MB-231 cells. These results provide an overview of the processes and targets of AnAc in representative ERα+ and TNBC breast cancer cells *in vitro*.

## Results and Discussion

### RNA-seq analysis of AnAc-regulated RNAs

To identify primary transcriptome changes in AnAc 24:1n5 (hereafter AnAc)-treated MCF-7 (ERα+) and MDA-MB-231 TNBC cells, cells were treated with the previously established IC_50_ concentrations of AnAc for MCF-7 (13.5 µM) and MDA-MB-231 (35.0 µM)^2^ prior to RNA isolation^6^. We note that AnAc has no overt effect on the viability of either cell line or cellular bioenergetics at that time^2,7^. The treatment duration was selected since primary gene targets have been identified in MCF-7 cells with a 6 h treatment^8^ and because the goal was to identify early transcriptome changes in response to AnAc in each cell line. For target analysis, only transcripts that showed a log2 fold-change greater than 1 (or −1 for repressed mRNAs) were included^9^.

**Figure 1:**
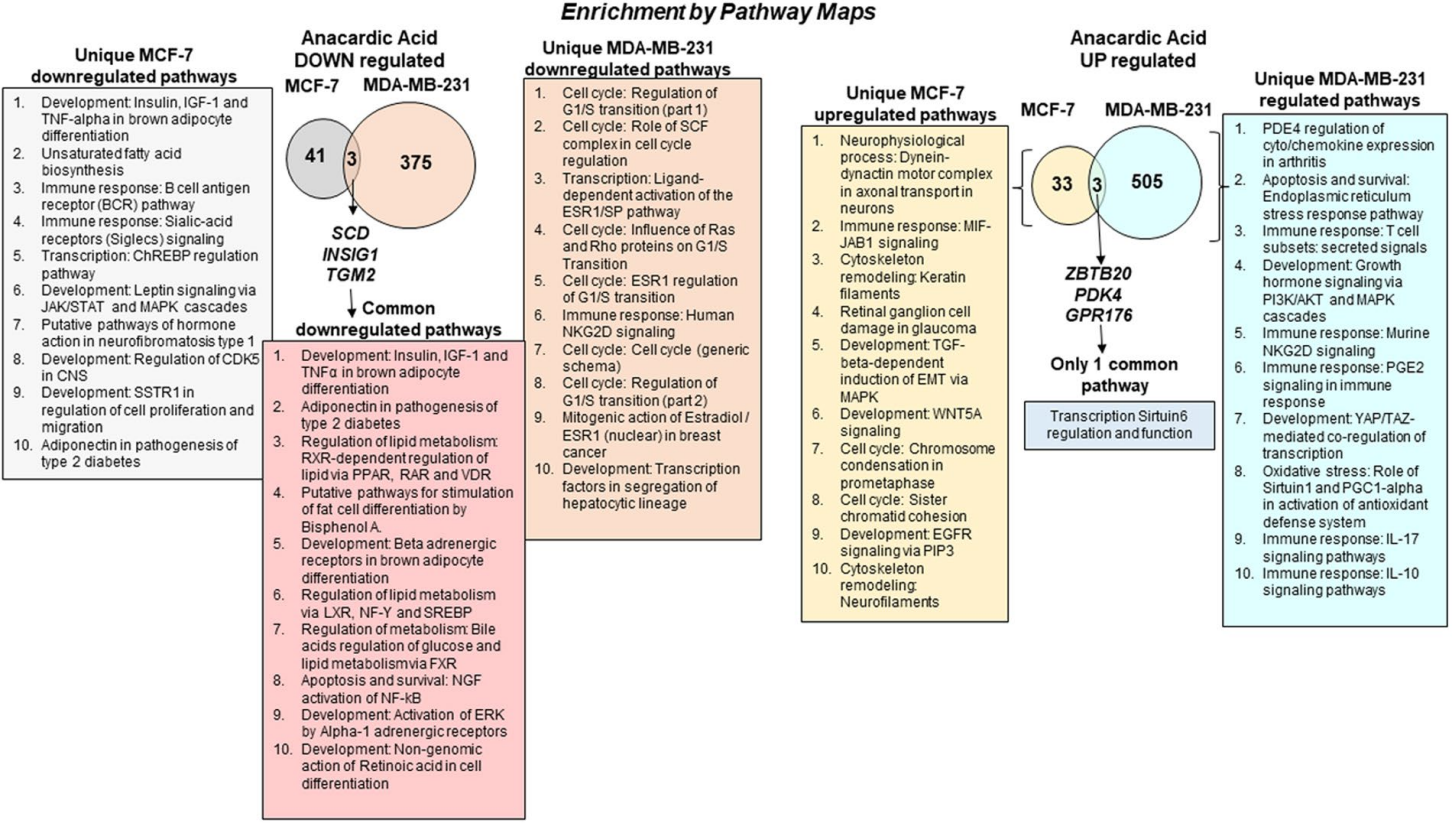
Enrichment analysis of RNA-seq data. Differentially expressed genes were identified in pairwise comparisons: MCF7 AnAc vs. MDA-MB-231 AnAc using the Tuxedo Suite of programs including Cufflink-Cuffdiff2. The Venn diagrams show the number of common and differentially expressed genes significantly downregulated (A) and upregulated (B). Pathway analysis was performed using GeneGo Pathways Software (MetaCore). The pathways identified for each comparison are listed in the order provided by MetaCore analysis.

Differentially expressed genes (DEGs) were identified for four pairwise comparisons (MCF-7 control *vs*. MCF-7 AnAc-treated; MDA-MB-231 control *vs*. MDA-MB-231 AnAc-treated; MCF-7 and MDA-MB-231 control *vs*. MCF-7 and MDA-MB-231 AnAc-treated; MDA-MB-231 control and AnAc-treated *vs*. MCF-7 control and AnAc treated) using cufflinks and cuffdiff ^6,10,11^. Table 1 shows the number of DEGs in each comparison. More genes were significantly changed in response to AnAc in MDA-MB-231 cells *vs* MCF-7 cells (Fig. 1). These data suggest selectivity of AnAc-induced transcriptional perturbations between these cell lines.

**Table 1:** Differentially expressed genes (DEGs).

Comparison	Cutoff	Number of DEGs
MCF-7 AnAc *vs*. control	P ≤ 0.05	80 (**↑36**, **↓44**)
MDA-MB-231 AnAc *vs*. control	P ≤ 0.05	886 (**↑508**, **↓ 378**)
All Cells AnAc *vs*. All Cells control^z^	P ≤ 0.05	25 (**↑11**, **↓14**)
All MCF-7 *vs*. All MDA-MB-231^y^	Q ≤ 0.01; |FC| ≥ 2	6124 (**↑3190**, **↓2934**)

The log2-fold change with zero value in the control conditions were arbitrarily set to one plus the maximum log2-fold change value and those with zero value in the treatment conditions were arbitrarily set to the minimum log2-fold change value minus one. The number of differentially expressed genes in each comparison is shown and the number of upregulated genes indicated with the upward arrow and downregulated genes indicated by downward arrow.

^Z^All Cells is the sum of both cell lines.

^Y^Sum of AnAc treatment and control for each cell line.

DEGs for each comparison were used for further analysis of enriched GO:BP^12,13^ and KEGG Pathways^14^ using CategoryCompare^15^. Table 2 and Supplementary Tables 1 and 2 list the top enriched GO:BP terms with p-value cutoff 0.001 for each of the four pairwise comparisons of DEGs while Supplementary Table 3 lists the top enriched KEGG pathways identified in AnAc-treated vs. control for each cell line. None of the top five GO terms for DEGs from (MCF-7 control and AnAc-treated) *vs*. (MDA-MB-231 control and AnAc-treated) (Supplementary Table 1) overlapped with those previously identified using Agilent microarrays to identify differential gene expression between non-treated MCF-7 *vs*. MDA-MB-231 cells^16^. The difference in these results may reflect changes in the rank order of differentially expressed genes of cell lines treated with AnAc or may reflect a difference in methodological approaches to analyze transcriptomes.

**Table 2:** Top enriched GO:BP terms for DEGs from MCF-7 and MDA-MB-231 AnAc *vs*. MCF-7 and MDA-MB-231 control using CategoryCompare.

GO term	Description	Gene#	P value
GO:0008203	Cholesterol metabolic process	3	0.00016
GO:0016125	Sterol metabolic process	3	0.00022
GO:0046165	Alcohol biosynthetic process	3	0.00025
GO:0006066	Alcohol metabolic process	4	0.00032
GO:1901617	Organic hydroxyl compound biosynthetic process	3	0.00062

For MCF-7 cells, only one GO term was identified for DEGs in AnAc cells: “Cellular response to acid chemical” with four genes in that pathway (Supplementary Table 2). In contrast, for AnAc-treated MDA-MB-231 cells five GO:BP terms were identified with 15–27 genes/GO:BP term and GO:BP terms related to the endoplasmic reticulum (ER) stress (ERS) and the unfolded protein response (UPR) as well as cholesterol and sterol biosynthetic responses (Supplementary Table 2). Since AnAc 24:1n5 inhibits cell proliferation in both cell lines after 24 h (18 h longer than the treatment here)^2^ these gene changes/pathways suggest mechanisms by which AnAc achieves its anti-proliferative effects differ between the two cell lines. Cholesterol and sterol biosynthesis take place in the ER and thus, the identification of these GO terms suggest that AnAc targets the ERS signaling pathway that is a survival factor in cancer^17,18^. Others reported that targeting MAPK-activation of the ERS response in TNBC cells, including MDA-MB-231, induces apoptosis^19^. We reported that 24 h treatment with 10–25 µM AnAc stimulates basal oxygen consumption and proton leak and reduces mitochondrial reserve in both MCF-7 and MDA-MB-231 cells, hallmarks of the apoptotic response^7^.

MetaCorenetwork enrichment analysis of the DEGs identified in AnAc-treated MCF-7 vs. MDA-MB-231 cells identified both cell line-specific and common enrichment pathways (Fig. 1) and GO processes (Supplementary Figure 1). MetaCore shortest direct pathways analysis of AnAc-regulated genes in MCF-7 cells suggests that increased JNK (MAPK8–10) is associated with higher *ERV6* (TEL1) and decreased *STIM1* associating with reduced *EGR1* that associates with lower *TGM2* (Supplementary Figure 2). Further discussion of these genes follows.

### AnAc-downregulated genes in common to MCF-7 and MDA-MB-231 cells

**Figure 2:**
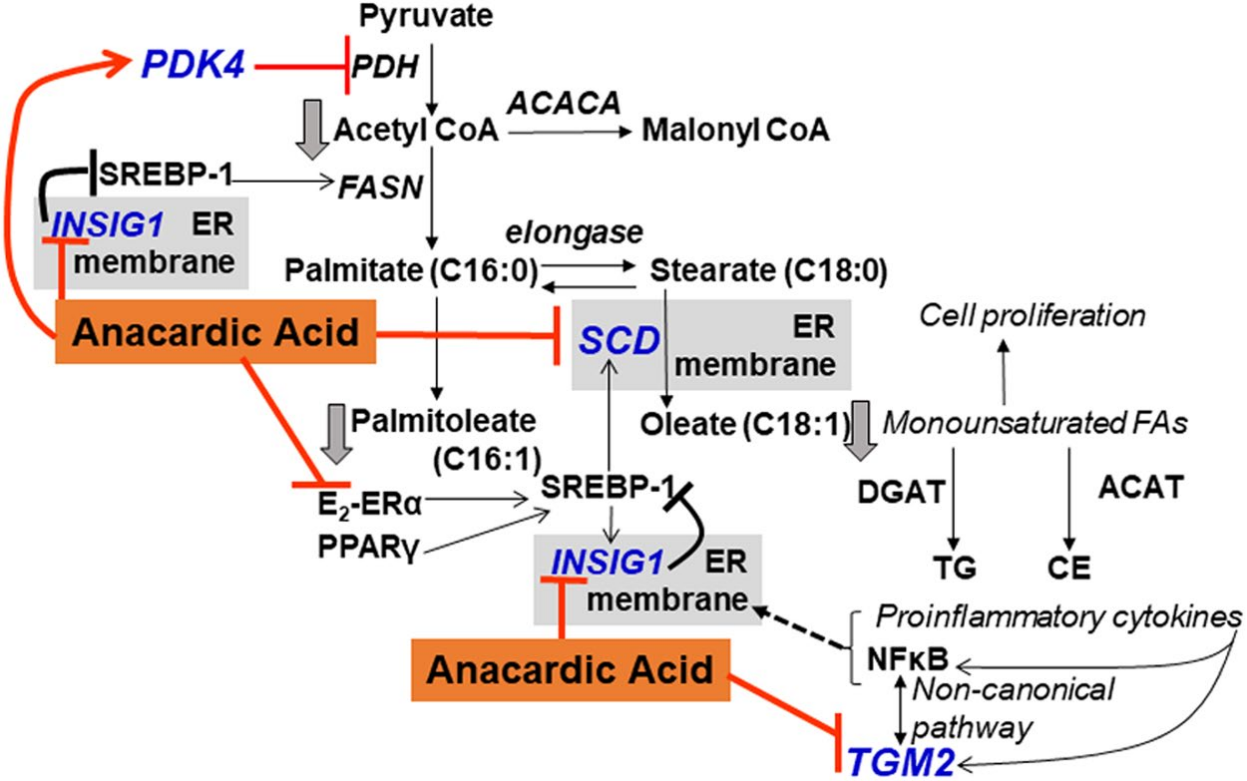
Modeling roles of four AnAc-regulated genes in MCF-7 and MDA-MB-231 cells. AnAc treatment reduced *SCD, INSIG1*, and *TGM2* and increased *PDK4* in both MCF-7 and MDA-MB-231 cells. PDK4 phosphorylates and inhibits pyruvate dehydrogenase (PDH), which would be expected to decrease acetyl CoA. SCD-1 (SCD, stearoyl-CoA desaturase-1) is a key rate-limiting enzyme for the synthesis of monounsaturated fatty acids. Endogenously synthesized monounsaturated fatty acids are metabolized by diacylglycerol acyltransferase (DGAT) to synthesize triglycerides (TG) or by acyl-CoA:cholesterol acyltransferase (ACAT) for cholesterol esters (CE) synthesis. INSIG1 anchors sterol regulatory element-binding protein (SREBP)/cleavage-activating protein (SCAP) in the endoplasmic reticulum (ER) membrane. SREBP-1 upregulates SCD and FASN transcription. TGM2 (transglutaminase 2) has various functions described in the text including activation of NFκB, which in turn regulates TGM2 expression. NFκB and proinflammatory cytokines, elevated in breast cancer, activate ER stress and SREBP-1.

AnAc treatment downregulated three genes (*SCD, INSIG1*, and *TGM2*) in both MCF-7 and MDA-MB-231 cells (Fig. 1). Hence, we would expect this downregulation to be ERα-independent. The third of the top 10 common downregulated pathways was “Regulation of lipid metabolism” (Fig. 1), which relates to *SCD* and *INSIG1*. The top GO processes identified were “response to fatty acid, triglyceride metabolic process”, and “regulation of steroid metabolic processes” (Supplementary Figure 1). Aberrant activation of lipid biosynthesis is involved in the early stages of breast cancer development (reviewed in^20^). Further, cell migration, invasion, and angiogenesis are all associated with increased SREBP-coordinated lipid biosynthesis^20^, results which may help to explain the more general, *i.e*., ERα-independent, breast cancer cell inhibition demonstrated by AnAc. We modeled the roles of the three AnAc-downregulated genes (*SCD, INSIG1*, and *TGM2*) and one of the three commonly AnAc-upregulated genes (*PDK4*) in lipid biosynthesis in Fig. 2. Each gene is discussed individually below. Supporting this model, ginkgolic acid (an AnAc congener from *Ginkgo biloba*) that suppresses pancreatic cancer cell viability, colony formation, migration, and invasion while increasing apoptosis, was reported to inhibit expression of enzyme targets involved in lipid biogenesis^21^.

AnAc reduced *SCD* (stearoyl-CoA desaturase, also called SCD1) transcript levels in both MCF-7 and MDA-MB-231 cells, suggesting an ERα-independent effect. However, different mechanisms may be responsible for *SCD* downregulation by AnAc in each cell line. For example, E_2_ stimulates *SCD* transcription by increasing transcription of SREBP-1C in MCF-7 cells^22^; thus, it is possible that the ERα-dependent NRAM activity of AnAc^2^ in MCF-7 contributes to *SCD* inhibition. Whereas an ERα-independent activity in MDA-MB-231 cells (or both cell lines) may be involved in the observed decrease in *SCD* transcript expression. *SCD* is anchored in the ER where it catalyzes the production of monounsaturated fatty acids (MUFAs, primarily oleic acid, oleate and palmitoleate) that are essential for membrane biogenesis in cancer cell proliferation^20^. Interestingly, oleic acid promotes proliferation in a number of breast cell lines, including MCF-7 and MDA-MB-231^23^. Importantly, oleic acid was also shown to inhibit apoptosis while palmitic acid (a precursor of oleic acid, Fig. 2) increased apoptosis in MDA-MB-231 cells^24^. *SCD* was also one of the most downregulated genes in primary breast cancer cells treated with 5 µM curcumin, another anticancer phytochemical^25^. SCD protein, not mRNA, was inhibited by *cis*−9, trans-11 and *trans*−10, *cis*−12 conjugated linoleic acid (CLA) isomers (45 μM) in MDA-MB-231 cells, but the mechanism was not identified^26^. A recent study demonstrated that SCD is essential for viability in three out of the four TNBC cell lines studied, including MDA-MB-231, that showed high sensitivity to SCD depletion^27^. Localized and systemic SCD deficiency causes ERS by increasing peroxisome proliferator active receptor ϒ (PPARϒ) Coactivator 1α (PGC-1α) and activates UPR (reviewed in^28^). “Apoptosis and survival: ERS response pathway” was upregulated by AnAc specifically in MDA-MB-231 cells (Fig. 1).

Upregulation of *SCD* in B16F10 mouse melanoma cells contributed to tumor formation and metastasis *in vivo* and CAY10566, a selective SCD inhibitor (IC_50_ ~7 nM), reduced lung metastasis *in vivo*^29^. That paper reported high *SCD* was associated with shorter disease free survival (DFS) in skin cutaneous and uveal melanoma, renal clear cell carcinoma, and pancreatic adenocarcinoma^29^. We used BreastMark^30^ and KM plotter^31^ to examine the correlation of *SCD* transcript expression and DFS in breast tumors (Supplementary Fig. 2). These analyses reveal that high *SCD* correlates with lower DFS in all breast and luminal A tumors, but does not reach statistical significance in TNBC, perhaps due to a lower number of tumor samples analyzed (Supplementary Fig. 2C). While the mechanism of AnAc inhibition of *SCD* expression reported here is unknown, the *SCD* promoter binds and is upregulated by AP1, C/EBPα, LXR, TR, SREBP1, NF1, NFY, SP1, C/EBPα, PPARα and PPARγ^32^, possible targets of AnAc action. Although 13 miRNAs were predicted to target the 3-UTR^33^, few have been experimentally validated. miRNAs downregulating SCD by direct interaction with its 3′UTR include miR-125b^34^, miR-199a-3p^35^, miR-212-5p^36^, and miR-27a^37^. None of these miRNAs were upregulated by AnAc with a 6 h treatment of MCF-7 or MDA-MB-231 cells^6^. Further studies will be necessary to delineate the mechanism for *SCD* downregulation in both cell lines.

AnAc inhibited *INSIG1* (Insulin Induced Gene 1) expression in MCF-7 and MDA-MB-231 cells (Fig. 1), again implying an ERα-independent mechanism. INSIG-1 anchors sterol regulatory element-binding protein (SREBP)/cleavage-activating protein (SCAP) in the ER membrane prior to its glycosylation or cholesterol binding which reduces its affinity to INSIG-1 allowing movement of SCAP/SREBP to the Golgi. Subsequent proteolytic activation of SREBP leads to its nuclear localization and upregulation of genes important in the uptake and synthesis of fatty acids, cholesterol, and phospholipids^38^. INSIG1 is a direct target of SREBP^39^. Supporting a role for *INSIG1* in cell viability, knockdown of *INSIG1* inhibited ZR-75-1 and MDA-MB-468 breast cancer and MCF-10A immortalized normal breast epithelial cell viability^40^. A methanol extract of black cohash (40 µg/ml) first stimulated (6 h) and then inhibited (24 h) *INSIG1* transcript expression in MDA-MB-453 breast cancer cells^41^. In contrast, gemcitabine, a nucleoside analog used to treat breast cancer, stimulated *INSIG1* expression in MCF-7 and MDA-MB-231 cells with MCF-7 cells showing higher *INSIG1* than MDA-MB-231 cells^42^.

AnAc reduced *TGM2* (transglutaminase 2) transcript levels in MCF-7 and MDA-MB-231 cells. TGM2 is a tumor and stem cell survival factor in breast and other cancers^43,44^. TGM2 has intrinsic and Ca^2+^dependent kinase activity and phosphorylates target proteins involved in cell proliferation and/or apoptosis^45^. TGM2 results in constitutive activation of NFκB via the noncannonical pathway, creating a feedback loop where NFκB upregulates TGM2 expression^46^. The increased NFκB and TGM2 results in drug-resistance and increased cancer stemness^47^. Knockdown of *TGM2* in MDA-MB-231 cells reversed epithelial to mesenchymal transition (EMT) and stimulated doxetaxel-induced apoptosis^48^. Overexpression of *TGM2* in MCF-10A cells inhibited basal oxygen consumption rate (OCR) and stimulated glycolysis as measured by extracellular acidification (ECAR) whereas *TGM2* knockdown in MCF-7 cells had the opposite effect^49^. Interestingly, we reported that AnAc stimulates basal OCR in both MCF-7 and MDA-MB-231 cells^7^, a result correlating with the reduction in *TGM2* transcript detected here.

### Genes uniquely inhibited by AnAc in MCF-7 cells downstream of NFκB

**Figure 3:**
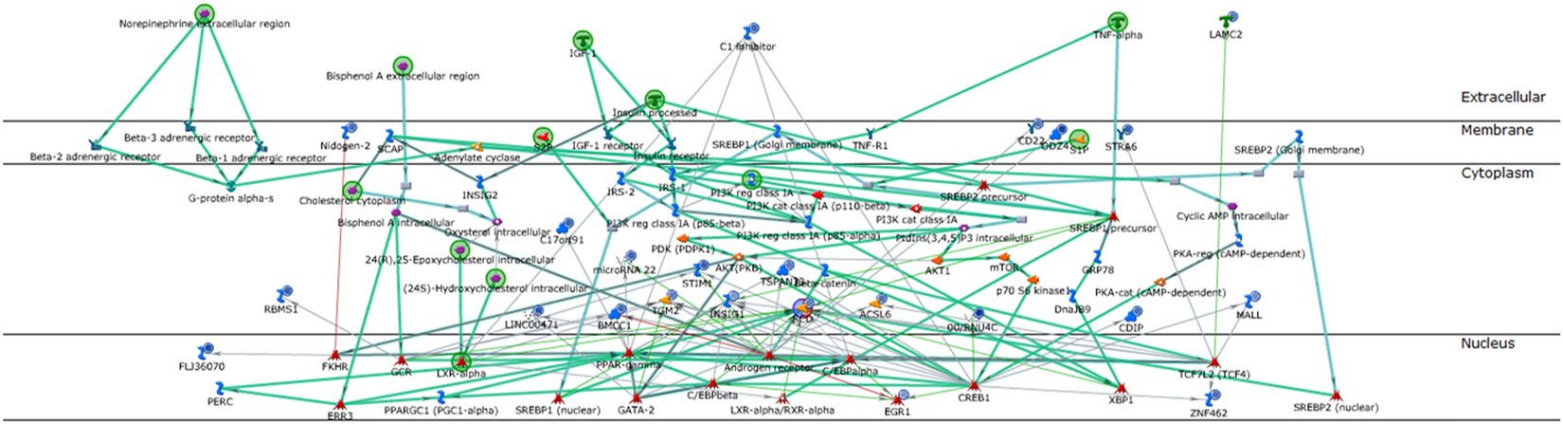
AnAc downregulated genes canonical pathway map for MCF-7 cells generated by MetaCore. Networks identified were: 1) SCD, LXRα, Insulin, Norepinephrine, IGF-1; 2) miR-22, CDIP; 3)MALL, NCOA2, E2 cytoplasm, hyaluronic acid extracellular, ESR1 (nuclear); 4) MRLC, CaMK II, STIM1, CARACM1, Ca; 5) uPAR, fibrinogen, BDKRB2, C2b, alpha-X/beta-2 integrin. All objects with the blue circle are downregulated by AnAc. The lines are connections that have been documented in the literature with green lines indicating canonical pathways.

Of the 44 gene transcripts identified as downregulated by AnAc in MCF-7 cells, 19 were matched to genes, 12 were protein-coding genes, and 13 are unannotated (Table 2). The canonical network analysis of the 19 genes downregulated by AnAc in MCF-7 generated by pathway enrichment analysis in MetaCore is shown in Fig. 3. The pathways and GO processes identified by MetaCore in the AnAc-downregulated genes in MCF-7 cells are shown in Fig. 1 and the pathway enrichment analysis of networks associated with DEGs in MCF-7 is shown in Supplementary Fig. 3. The top network for AnAc-downregulated genes centers on Acyl-CoA synthetase, *ACSL6, APBECH3, CDIP*, and *EGR1* (Supplementary Fig. 4). MetaCore transcription factor network analysis identified 30 transcription factors in the DEGs in MCF-7 cells including CREB, p53, ESR1 (ERα), and RelA/NFκB (Supplementary Fig. 5). AnAc was previously reported to inhibit NFκB activation in KBM-5 cells^50^.

### AnAc inhibits tumor necrosis factor α (TNFα)-stimulated NFκB in MCF-7 cells

**Figure 4:**
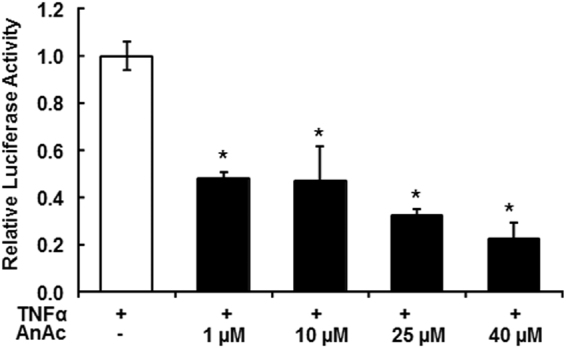
AnAc inhibits TNFα-induced NFκB luciferase reporter activity in transiently transfected MCF-7 cells. MCF-7 cells were transfected with a NFκB response element luciferase reporter and a Renilla reporter for 48 h. Cells were treated with 10 ng/ml TNFα EtOH (vehicle control, open bar, and 1–40 µM AnAc for 6 h before performing dual luciferase assay. Values are the average of three separate wells in one experiment ± SEM. *p < 0.01 versus EtOH control (open bar).

The pathway enrichment analysis of networks associated with downregulated genes in AnAc-treated MCF-7 cells (Supplementary Fig. 6) suggests involvement of NFκB. MCF-7 cells have low NFκB activity^51^. We examined if AnAc would inhibit TNFα-stimulated NFκB luciferase reporter activity in transiently transfected MCF-7 cells (Fig. 4). Consistent with the DEGs identified in RNA-seq analysis of AnAc-treated MCF-7 cells and with the AnAc inhibition of *TGM2* that stimulates NFκB expression and activity (modeled in Fig. 2), AnAc inhibited TNFα-stimulated NFκB luciferase reporter activity (Fig. 4). We reported that AnAc inhibits NFκB target gene *CCND1* expression in MCF-7 cells^2^ and AnAc reduced *CCND1* in MDA-MB-231 cells (Supplementary Table 5), results in agreement with the antiproliferative activity of AnAc.

### Genes downregulated in MCF-7 cells by AnAc

We hypothesized that the ERα antagonist activity of AnAc^6^ might be involved in the decrease of selected gene transcripts in AnAc-treated MCF-7 cells and not in MDA-MB-231 cells. Based on our data and the literature reports cited below, we suggest that this hypothesis may support the downregulation of *ZNF462*, *MALL (BENE)*, and *EGR1* transcript expression by AnAc in MCF-7 cells.

AnAc inhibited *ZNF462* expression in MCF-7 cells (Table 3). *ZNF462* was identified as a putative target of miR-210 which is upregulated by HIF-1α in pancreatic cancer^52^. A search in the NURSA Transcriptomine database^53^ revealed that both E_2_ and 4-OHT increase transcript levels of *ZNF462* in MCF-7 cells. Thus, the NRAM activity of AnAc with ERα^2^ may be responsible for the observed decrease in *ZNF462* expression.

**Table 3:** Genes significantly inhibited in MCF-7 cells after 6 h. of 13.5 µM anacardic acid (AnAc) treatment.

Gene	Control	AnAc	P value	Description	Top 3 GO terms
*RBMS1*	35.69	15.14	5.00E-05	RNA Binding Motif Single Stranded Interacting Protein	GO:0006396: RNA processing; GO:0006260: DNA replication; GO:0003697: single-stranded DNA binding
*SCD*	400.61	234.01	0.001	stearoyl-CoA desaturase	GO:0004768: stearoyl-CoA 9-desaturase activity;GO:0006633: fatty acid biosynthetic process;GO:0005506: iron ion binding
*RNU4-2*	104.59	27.41	0.0016	U4 Small Nuclear 2	
*TSPAN33*	104.59	27.41	0.0034	Tetraspanin 33	GO:0016021; integral to membrane
*STIM1*	25.05	10.56	0.0038	Stromal Interaction Molecule 1	GO:0006812: cation transport; GO:0043234: protein complex; GO:0005515: protein binding
*GATA6-AS1*	1.19	0.19	0.0047	GATA6 Antisense RNA 1 (Head To Head)	
*TGM2*	3.82	2.27	0.0069	Transglutaminase 2	GO:0043277: apoptotic cell clearance; GO:0018149: peptide cross-linking; GO:0060662: salivary gland cavitation
*CD22*	1.04	0.57	0.0161	Sialic Acid Binding Ig-Like Lectin 2	GO:0009897:external side of plasma membrane; GO:0005887: integral to plasma membrane; GO:0005515:protein binding
*RN7SL389P*	1.15	0.00	0.02285	RNA, 7SL, Cytoplasmic 389, Pseudogene	
*CDIP1*	1.25	0.61	0.0229	Cell Death-Inducing P53 Target 1	GO:0042771: intrinsic apoptotic signaling pathway in response to DNA damage by p53 class mediator; GO:0033209: tumor necrosis factor-mediated signaling pathway; GO:0006915:apoptotic process
*INSIG1*	86.16	64.07	0.0285	Insulin Induced Gene 1	GO:1901303: negative regulation of cargo loading into COPII-coated vesicle; GO:0032937: SREBP-SCAP-Insig complex GO:0032933: SREBP signaling pathway
*LAMC2*	5.68	3.17	0.03285	Laminin Subunit Gamma 2	GO:0005610: laminin-5 complex; GO:0034329: cell junction assembly; GO:0031581: hemidesmosome assembly
*SAMD9*	2.16	1.44	0.0353	SAM Domain-Containing Protein 9	GO:0043231: intracellular membrane-bounded organelle; GO:0005737: cytoplasm; GO:0005515: protein binding
*ZNF462*	1.35	0.67	0.0413	Zinc Finger Protein 462	GO:0006325: chromatin organization; GO:0043392: negative regulation of DNA binding; GO:0003677: DNA binding
*MIR22HG*	12.49	5.52	0.0421	MIR22 Host Gene	
*MALL*	15.93	10.98	0.04315	Mal, T-Cell Differentiation Protein-L; Protein BENE	GO:0030136: clathrin-coated vesicle; GO:0016023: cytoplasmic membrane-bounded vesicle; GO:0042632: cholesterol homeostasis
*RNU5B-1*	76.15	25.32	0.0453	U5B Small Nuclear 1	
*RNU4-1*	57.57	26.33	0.0467	U4 Small Nuclear 1	
*EGR1*	17.13	13.03	0.0474	Early Growth Response 1	GO:0072303: positive regulation of glomerular metanephric mesangial cell proliferation GO:0072110: glomerular mesangial cell proliferation; GO:0071873: response to norepinephrine

Genes are arranged from the most to least statistical significance. Values are FKPM. All values are significantly different, P < 0.05. The GO terms are listed in the order provided by DEG analysis and subsequent network analysis in MetaCore Gene Ontology (GO) algorithm to characterize the biological pathways altered by AnAc.

AnAc inhibited *MALL* (BENE) expression in MCF-7 cells (Table 3). *MALL* is a member of the proteolipid family that localizes in glycolipid- and cholesterol-enriched membrane rafts and it interacts with CAV-1. A search in the NURSA Transcriptomine database^53^ revealed that both E_2_ (100 nM, 12 h) and fulvestrant (100 nM, 12 h) inhibited *MALL* transcript expression in MCF-7 cells, a result that seems contradictory for an ERα-mediated response, but not one mediated by GPER1 that binds E_2_ and fulvestrant as agonists with Kd = 3–6 and 10–100 nM, respectively^54^. No references regarding the regulation or function of *MALL* in breast or other cancers were found in PubMed.

AnAc inhibited *EGR1* expression in MCF-7 cells (Table 3). *EGR1* is a member of the immediate-early gene group of transcription factors whose transcription is rapidly increased by E_2_ in MCF-7 cells^55^ and deleted in ERα- breast tumors where it is thought to be a tumor suppressor^56^. 4-OHT suppresses E_2_-simulated *EGR1* transcription in MCF-7 cells^57^. Thus, the repression of *EGR1* expression by AnAc in MCF-7 cells may reflect its ability to block E_2_-induced gene transcription by inhibiting ERα-DNA binding^2^.

AnAc had the greatest inhibitory activity on *RBMS1* (also called MSSP, MSSP-1) transcript expression in MCF-7 cells (Table 3). RBMBS1/MSSP1 has been implicated in DNA replication, gene transcription, cell cycle progression and apoptosis^58^. Increased RBMBS1/MSSP-1 was associated with cisplatin resistance in ovarian cancer cells^59^. A search in the NURSA Transcriptomine database^53^ revealed that E_2_ inhibited *RBMS1* expression in MCF-7 cells. Another dietary anticancer agent, bromelain, a mixture of proteolytic enzymes found in pineapples (reviewed in^60^) also downregulated *RBMS1* expression in MCF-7 cells^61^. Thus, two dietary phytochemicals inhibit *RBMS1* expression in MCF-7 cells. *RBMS1* is downregulated by miR-383^62^, but miR-383 was not regulated by AnAc treatment of MCF-7 cells^6^. It is unknown how *RBMS1* downregulation by AnAc in MCF-7 cells contributes to AnAc’s antiproliferative/pro-apoptotic activity in these cells.

No information about *RNU4-2, TSPAN33* (PEN, an alternative protein name), *GATA6*-*AS1, RN7SL389P, CDIP1, RNU5B-1*, or *RNU4-1* in breast or other cancers was found in PubMed. A search in the NURSA Transcriptomine database^53^ revealed no reports *of RNU4-2, TSPAN33, GATA6-AS1, RN7SL389P, RNU5B-1*, or *RNU4-1*, in mammary gland/human transcriptome data sets curated in that collection. However, *CDIP1* was repressed by 30 pM E_2_ in MCF-7 cells with 48 h of treatment whereas ERα knockdown increased *CDIP1* expression in MCF-7 cells^63^, implying a possible role for E_2_-ERα in reducing *CDIP1* transcript levels. *CDIP* is a key downstream effector of p53‐dependent apoptosis^64^. In contrast to our findings with AnAc, phytochemicals (xanthones) from *Garcinia* increased *CDIP* transcript expression in NCI-H1650 lung adenocarcinoma cells which correlated with antiproliferative activity^65^.

AnAc inhibited *STIM1* and *LAM2* expression in MCF-7 cells (Table 3). STIM1 is an ER Ca^2+^ sensor that triggers Ca^2+^ influx by activating store-operated calcium entry and is involved in the TGF-β-induced suppression of cell proliferation^66^. Inhibition of *STIM1* expression by TGF-β in MCF-7 and MDA-MB-231 cells inhibited cell proliferation^66^. LAMC2 is a subunit of the basement membrane protein laminin-332 that interacts with CD44 on the membrane of breast cancer cells, stimulates cell migration, and is regarded as a typical cancer invasion marker corresponding with poor patient prognosis^67^. Thus, the inhibition of *LAMC2* by AnAc provides a potential mechanism for the anti-proliferative activity of AnAc in breast cancer cells *in vitro*.

AnAc inhibited *SAMD9* expression in MCF-7 cells (Table 3). *SAMD9* has antiproliferative activity in H1299 lung adenocarcinoma cells *in vitro* and in tumor xenografts *in vivo*^68^. Further, *SAMD9* expression is lower in breast tumors than normal breast^69^. A search in the NURSA Transcriptomine database^53^ revealed that knockdown of ERα coactivator SRC-1/NCOA1 increased *SAMD9* in LY2 endocrine-resistant cells derived from MCF-7 cells^70^. However, we did not detect any change in *NCOA1* transcript expression in our RNA-seq data of AnAc-treated MCF-7 cells. The inhibition of putative tumor suppressor *SAMD9* expression by AnAc seems to conflict with AnAc’s anti-cancer activity in MCF-7 cells. However, this may be a time-dependent effect since we analyzed gene expression after only 6 h of AnAc treatment.

### LncRNA MIR22HG is inhibited by AnAc in MCF-7 cells

AnAc reduced *MIR22HG* lncRNA transcript in MCF-7 cells (Table 3). *PTGES3* is a long non-coding RNA (lncRNA) and is the host gene for miR-22 that functions as a tumor suppressor by repressing *CDK6, CCNA2, SP1*, and *PTGES3* (p23) (reviewed in^71^). Chemical stressors (24 h) 100 µM cycloheximide, 100 µM hydrogen peroxide, 1 µM cadmium nitrate, or 100 nM arsenic trioxide stimulated *MIR22HG* expression in human-induced Pluripotent Stem Cell (hiPSC) line 201B7^72^. Ischemia increased *MIR22HG* in a mouse hind limb ischemia model^73^ and *MIR22HG* was downregulated in human lung adenocarcinomas^74^. miR-22 directly targets *ESR1* (ERα)^75^. Further studies will be needed to address the role of inhibition of *MIR22HG* in downstream effects of AnAc in MCF-7 cells.

### Genes downregulated by AnAc in MDA-MB-231 cells

AnAc inhibited the expression of 378 genes in MDA-MB-231 cells (Table 1, Supplementary Table 4). The top 10 downregulated pathways identified by MetaCore enrichment analysis is shown in Fig. 1. Not surprisingly since AnAc inhibits MDA-MB-231 cell proliferation^2^, the top two pathways involve cell cycle regulation. Intriguingly, third on the list is “Transcription: Ligand-dependent activation of the ESR1/SP pathway”. Although MDA-MB-231 cells are ERα-, they were reported to express ERβ protein^76^. Recent studies indicate that activation of peroxisome proliferator activated receptor (PPAR) δ and inhibition of PPARγ stimulate ERα expression in ERα- mouse mammary tumors^77,78^. We did not detect *ESR1* or any of the PPAR genes among those regulated by AnAc in MDA-MB-231 cells. A PubMed search for RNA-seq studies in MDA-MB-231 cells treated with an ‘anti-cancer drug’, found only one report^79^. A comparison of the genes regulated by the ruthenium-derived compound NAMI-A anti-metastasis compound in MDA-MB-231 cells and AnAc-regulated DEGs identified three common genes: *HES1*, *RIPK4* (downregulated by AnAc, upregulated by NAMI-A) and *SPRY1* (upregulated by AnAc and NAMI-S) (Supplementary Table 5 and^79^). These data confirm that anticancer compounds work through distinct processes in MDA-MB-231 cells.

**Figure 5:**
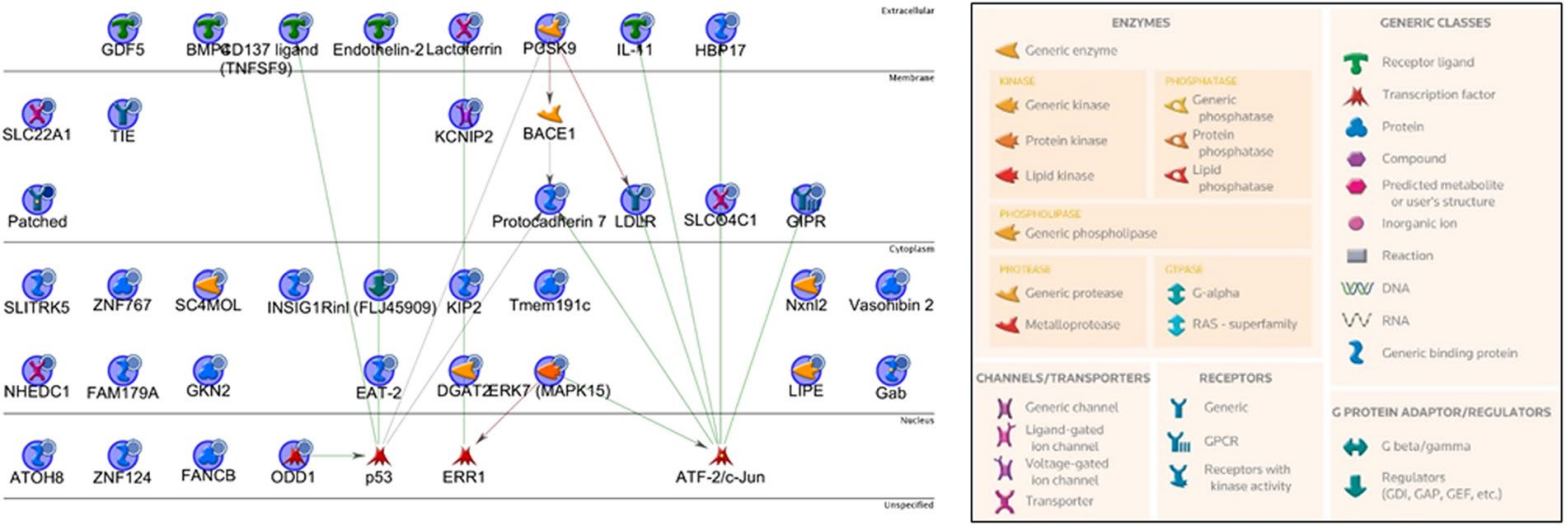
MetaCore network analysis of the top 50 genes downregulated by AnAc in MDA-MB-231cells. Shown is Dijkstra’s shortest paths algorithm calculating the shortest directed paths between the top 50 genes downregulated by AnAc in MDA-MB-231 with two steps in the path and genes fitting this model were arranged by cellular location. The network of up-regulated genes centers on p53, ERR1, and ART-2/c-Jun. The MetaCore legend is shown at the right.

Network analysis of the top 50 downregulated genes in AnAc-treated MDA-MB-231 cells using Dijkstra’s shortest paths algorithm calculating the shortest direct paths with 2 steps. This analysis shows connections of a number of downregulated genes to each other and to p53, ERR1, and ARF-2/c-Jun (Fig. 5). The function of the top 18 genes downregulated by AnAc in MDA-MB-231 cells, based on statistical evaluation, are summarized in Supplementary Table 6.

### Three genes upregulated by AnAc in both MCF-7 and MDA-MB-231 cells

**Figure 6:**
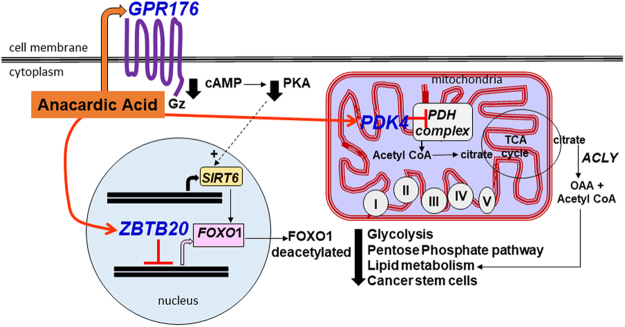
Modeling the potential cellular roles of three genes upregulated by AnAc in MCF-7 and MDA-MB-231 cells. As indicted in Fig. 1, MetaCore analysis identified only one pathway for these three common upregulated genes: Transcription Sirtuin6 (SIRT6) regulation and function. AnAc increased GPR176 in both cell lines. GPR176 is a GZ-coupled receptor that decreases cAMP, which would be expected to decrease PKA, both of which would maintain SIRT6 protein by preventing its ubiquitinylation and proteasome-mediated degradation (dashed arrow,+). AnAc increased ZPTB20, a transcriptional repressor that inhibits FOXO1 transcription. SIRT6 deacetylates FOXO1 which decreases its nuclear localization, hence reducing glycolysis, pentose phosphate pathway, lipid metabolism, and cancer stem cell biogenesis. AnAc increased PDK4 in both MCF-7 and MDA-MB-231 cells. PDK4 phosphorylates and inhibits pyruvate dehydrogenase (PDH), which would be expected to decrease acetyl CoA, possibly inhibiting the TCA cycle, oxidative phosphorylation, and FA biosynthesis. Taken together, the observed gene changes are commensurate with the observed ability of AnAc to inhibit the proliferation of these two breast cancer cell lines.

AnAc stimulated *ZBTB20*, *PDK4*, and *GPR176* expression in MCF-7 and MDA-MB-231 cells (Fig. 1). We would expect these increases to be ERα-independent. MetaCore analysis identified only one pathway in common for these three upregulated genes: “Transcription Sirtuin6 (*SIRT6*) regulation and function”. SIRT6, an established chromatin regulatory protein, is a tumor suppressor that has three enzymatic activities: deacetylase, ADP-ribosyltransferase, and de-fatty-acylase^80^. SIRT6 overexpression inhibited breast cancer stem cell biogenesis in cells with a PI3K mutation and murine PyMT mammary tumor progression *in vivo*^81^. Hence if *ZBTB20*, *PDK4*, and *GPR176* indeed stimulate SIRT6 function, *e.g*., by increasing transcription, stabilizing the protein, or increasing its activity by increasing NAD+ and free FAs^80^, this could provide a mechanism by which AnAc inhibits MCF-7 and MDA-MB-231 viability. Further study will be needed to examine AnAc-mediated metabolic changes in these cells. Figure 6 is model of the potential cellular functions of the three AnAc-upregulated genes common to MCF-7 and MDA-MB-231 breast cancer cells.

ZBTB20 (also called DPZF, HOF, and ZNF288) is a member of the POK (POZ and Krüppel) family of transcriptional repressors^82^. *ZBTB20* is upregulated in HCC^82^ and non-small cell lung cancer (NSCLC)^83^, but downregulated in primary prostate cancer samples and metastases^84^. *ZBTB20* was among the genes downregulated in primary breast tumors from patients treated with aromatase inhibitors, either letrozole or anastrozole for 2 weeks prior to surgery^85^. In NSCLC, ZBTB20 downregulated FOXO1 by binding to its 5′ promoter^83^. Although *FOXO1* was not among the AnAc-regulated genes in MCF-7 cells, *FOXO1* was upregulated by AnAc in MDA-MB-231 cells (Supplementary Table 5), a result opposite of what might be anticipated if an AnAc-mediated increase in ZBTB20 inhibits FOXO1 expression. However, SIRT6 deacetylates FOXO1 leading to its export from the nucleus to the cytoplasm and hence inhibiting its transcriptional activity, as well as glycolysis, the pentose phosphate pathway, lipid metabolism, and breast cancer stem cell biogenesis^81,86^. ZBTB20 was recently identified as a tumor suppressor that cooperates with PTEN to prevent malignant progression in prostate cancer^84^. We did not detect *PTEN* among the AnAc-regulated genes in either cell line. Together these studies suggest that an increase in tumor suppressor ZBTB20 may contribute to the anti-proliferative activity of AnAc in breast cancer cells.

When active, PDK4 phosphorylates and inhibits pyruvate dehydrogenase (PDH) which converts pyruvate to acetyl-CoA for the TCA cycle or fatty acid biosynthesis. Thus, an increase in PDK4 would decrease glucose carbon flux into the TCA cycle and lipid biosynthesis, consistent with the three common downregulated genes modeled in Fig. 2. Estrogen-related receptor gamma (ERRγ, *ESRRG*) is a major activator of PDK4^87,88^, but we did not find *ESRRG* among the AnAc-regulated genes in either cell line. *PDK4* expression was higher in TAM-resistant MCF-7 cells than parental MCF-7 cells and siPDK4 sensitized the cells to growth inhibition by fulvestrant^89^. Increased PDK4 results in an increase in mitochondrial ROS^88^ and ERS^90^, findings commensurate with MetaCore-identified upregulated pathway 2: “Apoptosis and Survival: ERS response” in MDA-MB-231 cells (Fig. 1).

AnAc increased *GPR176*, orphan G-protein coupled receptor (GPCR), in both MCF-7 and MDA-MB-231 cells. GPR176 is an evolutionarily conserved, vertebrate class A orphan GPCR that acts in a ligand-independent manner and can repress adenyl cyclase^91^. A sequence-structure based alignment of known GPCRs to identify putative ligand associations for orphan GPRs posited free fatty acids as ligand for GPR176^92^. This association raises an interesting speculation that AnAc may activate GPR176. Since an increase in active GPR176 would be expected to decrease cAMP, and hence decrease active PKA, we wondered if cAMP regulates SIRT6. Indeed an increase in cAMP-activated PKA reduced SIRT6 by promoting its ubiquitin-proteasome-mediated degradation^93^. This is modeled in Fig. 6. Taken together, the observed common gene changes in response to AnAc correspond with the observed ability of AnAc to inhibit the proliferation of these two breast cancer cell lines.

### Genes uniquely increased by AnAc in MCF-7 cells

Of the 36 gene transcripts increased by AnAc in MCF-7 cells (Table 1, Fig. 1), 12 were protein-coding genes that were not affected by AnAc in MDA-MB-231 cells (Table 4). Only 2 genes have been reported to be regulated by ERα; hence, both ERα and ERα-independent mechanisms are likely involved in the AnAc-regulated changes in their expression specifically in MCF-7 cells. MetaCore network analysis of these AnAc-upregulated genes identified only one direct interaction between *JNK* and *ETV6* (Supplementary Fig. 7). When analyzed by pathway enrichment using two steps, MetaCore calculated ‘hub proteins’ including Vimentin, JNK, PKA, and GSK3beta (Supplementary Fig. 8).

**Table 4:** AnAc-regulated lncRNAs in MDA-MB-231 cells.

Gene nc RNA	control	AnAc	log2FC (AnAc/ctrl)	P value	function	Ref.
*GRM5-AS1*	0.811	0.046	−4.133	0.037		
*ARHGEF26-AS1*	1.703	0.171	−3.317	0.023		
*CFLAR-AS1*	0.997	0.127	−2.976	0.004	potential circulating biomarker (up) for early esophageal squamous cell carcinoma	^106^
*HOXB-AS3*	0.392	0.147	−1.413	0.023		
*UBL7-AS1*	2.004	0.803	−1.320	0.011	Interrupted by a translocation joining RUNX1 to UBL7-AS1 in myeloid leukemia	^107^
*LINC00639*	0.465	0.204	−1.187	0.027		
*MIR210HG*	14.986	6.997	−1.099	0.001	Induced by hypoxia in human HKC-8 kidney cells and HUVECs; higher in glioma tumor tissue and patient serum	^73,108,109^
*MIR663A*	0.860	0.443	−0.956	0.029		
*ASB16-AS1*	4.360	2.633	−0.728	0.033		
*LINC01125*	1.335	0.829	−0.686	0.023		
*LINC00638*	0.741	0.470	−0.655	0.037		
*MIR24-2*	6.318	4.077	−0.632	0.004		
*LINC00116*	70.421	55.217	−0.351	0.048		
*LINC00973*	37.637	48.114	0.354	0.033		
*LINC01003*	1.684	2.378	0.498	0.039		
*LINC00669*	1.126	1.711	0.603	0.016		
*LINC00662*	14.109	28.440	1.011	2.00E-04		
*SLC2A1-AS1*	0.319	0.809	1.344	0.014		

LINC = Long Intergenic Non-Protein Coding RNA. Genes are arranged from the most downregulated to most upregulated based on log2FC values. Values are gene expression are FKPM. All values are significantly different, P < 0.05.

The mixed agonist/antagonist activity of AnAc in MCF-7 cells^2^ may play a role in the increase in transcript level of *VIM* (vimentin), *TMC5*, a multi-pass membrane protein, *ETV6*, and *MAPK10* (Table 5). This suggestion is based on data in the NURSA Transcriptomine database^53^ that revealed E_2_ inhibited *VIM*, *TMC5*, *ETV6*, and *MAPK10* expression in MCF-7 cells. We reported that AnAc reduced miR-378g in MCF-7 cells^6^ and note that miR-378 targets *VIM*^94^, suggesting a possible mechanism for the increase in *VIM* by relief of repression. *ETV6* is a dominant-acting cancer gene that appears to be a site of frequent genomic rearrangements in human breast tumors, but is not amplified^95^. The mechanism for AnAc regulation of *ETV6* and *MAPK10* will require further investigation.

**Table 5:** Genes uniquely upregulated by anacardic acid in MCF-7 cells after 6 h of treatment with 13.5 µM.

Gene	Control	AnAc	P value	Description	Top 3 GO terms	Gene function
*CELSR3*	0.10084	8.2254	5.00E-05	Cadherin, EGF LAG Seven-Pass G-Type Receptor 3, a GPCR	GO:0007413:axonal fascicula-tion: GO:0032880: regulation of protein localization: GO: 0007166: cell surface receptor signaling pathway	CELSR3 is a multi-pass membrane protein. CELSR3 is methylated in neuroendocrine^110^ and oral tumors^111^.
*CACNG8*	3.82558	9.87498	5.00E-05	Calcium Channel, Voltage-Dependent, Gamma Subunit 8	GO:0030666:endocytic vesicle membrane; GO:2000311: regulation of alpha-amino-3-hydroxy-5-methyl-4-isoxazole propionate selective glutamate receptor activity;	CACNG8 is a transmembrane voltage-gated calcium channel subunit regulatory protein.
*FAM73A*	0.45163	1.4673	0.00045	Family With Sequence Similarity 73	GO:0016021; integral to membrane	FAM73 (Mitoguardin-1, MIGA1) is a regulator of mitochondrial fusion.
*NTRK3*	0.47030	1.2800	0.0039	Neurotrophic Receptor Tyrosine Kinase 3	GO:0048691:positive regulation of axon extension; GO:0038179: neurotrophin signaling; GO:0019056: modulation by virus of host transcription	NTRK3 is a membrane bound receptor that binds neurotrophin and activates MAPK signaling.
*LEKR1*	0.59333	1.64146	0.0044	Leucine, Glutamate And Lysine Rich 1	GO:0005840:ribosome GO:0006412:translation GO:0003735:structural constituent of ribosome	A missense mutation in the *LEKR1* gene was associated with epithelial ovarian cancer risk^112^.
*HIST1H1D*	0.74898	1.6380	0.0051	Histone Cluster 1, H1d	GO:0000790:nuclear chromatin GO:0006334:nucleosome assembly; GO:0000786: nucleosome	nucleosomal linker histone increased after *PPM1D* knockdown ZR-75-1 breast cancer cells^113^.
*VIM*	105.535	166.93	0.0061	Vimentin	GO:0006921:cellular component disassembly involved in apoptosis; GO:0060020: Bergmann glial cell differentiation; GO:0045109: intermediate filament organization	
*RN7SL2*	0.24437	2.3353	0.00655	RNA, 7SL, Cytoplasmic 2		RNA component of the signal recognition particle, a cytoplasmic ribonucleo-protein complex
*ARHGEF26-AS1*	53.1195	76.392	0.0079	ARHGEF26 Antisense RNA1		Non-coding RNA
*RABGGTB*	0.49542	2.43989	0.00925	Rab Geranylgeranyltransferase Beta Subunit	GO:0018344:protein geranylgeranylation GO:0004663:Rab geranyl-geranyltransferase activity; GO:0006464:cellular protein modification process	RABGGTB is the beta-subunit of the enzyme Rab geranylgeranyl-transferase (RabGGTase).
*TMC5*		1.43847	0.0121	Transmembrane Channel-Like 5	GO:0016021:integral to membrane; GO:0006811:ion transport	A multi-pass membrane protein inhibited by E_2_ in MCF-7 cells^53^.
*RPS4XP16*	1.97726	3.21612	0.013	Ribosomal Protein S4X Pseudogene 16		
*ETV6*	0.94319	1.5434	0.0285	Ets Variant, TEL1 oncogene, transcription factor	GO:0043565:sequence-specific DNA binding; GO:0006355: regulation of transcription, DNA-dependent; GO:0019904: protein domain specific binding	*ETV6* interacts with corepressors: NCOR and SMRT (reviewed in^114^). *ETV6* is downstream of JNK (Supplementary Fig. 7).
*MAPK10*	0.43154	1.19202	0.0399	Mitogen-Activated Protein Kinase 10	GO:0007258:JUN phosphorylation; GO:0034146: TLR5 signaling pathway; GO:0038124: TLR6:TLR2 signaling pathway	MAPK10 is a proapoptotic protein^115,116^. Higher in luminal breast cancer cell lines was associated with sensitivity to everolimus^117^
*HIST1H2AK*			0.04925	Histone Cluster 1, H2ak	GO:0006334:nucleosome assembly; GO:0000786: nucleosome; GO:0019899: enzyme binding	E_2_ inhibits *HIST1H2AK* expression in MCF-7 cells^53^.

Genes are arranged from the most to least statistical significance. Values are FKPM. All values are significantly different, P < 0.05. The GO terms are listed in the order provided by DEG analysis and subsequent network analysis in MetaCore.

### Genes uniquely increased by AnAc in MDA-MB-231 cells

**Figure 7:**
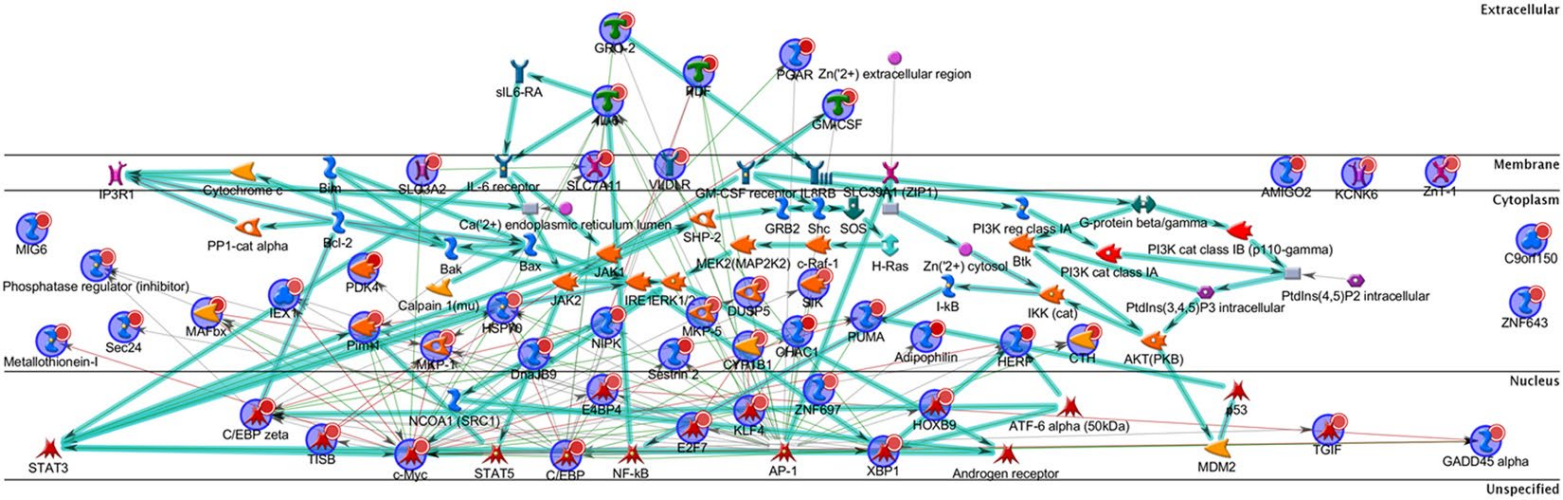
MetaCore network analysis of the top 50 genes upregulated by AnAc in MDA-MB-231cells. Shown is Dijkstra’s shortest paths algorithm calculating the shortest directed paths between the top 50 genes downregulated by AnAc in MDA-MB-231 with one step in the path and genes fitting this model were arranged by cellular location. The network of up-regulated genes shows a number of nodes including c-MYC, C/EBP, XBP1, E4BP4, AP-1, NFκB, STAT3, STAT5, and PIM1.

AnAc uniquely upregulated 503 genes in MDA-MB-231 and not in MCF-7 cells (Table 1, Fig. 1, and Supplementary Table 5). The top 10 upregulated pathways identified by MetaCore enrichment analysis are shown in Fig. 1 and the top 10 GO processes in Fig. 2. Notably, the ERS response pathway was increased by AnAc in MDA-MB-231 cells. Network analysis of the top 50 upregulated genes using Dijkstra’s shortest paths algorithm calculating the shortest direct paths with one step shows connections of a number of upregulated genes to each other and to STAT3, STAT5, NFκB, AP1, and AR in MDA-MB-231 cells (Fig. 7). The top 10 genes upregulated uniquely by AnAc in MDA-MB-231 cells and their roles in the ER stress response are summarized in Supplementary Table 7.

When cellular stresses perturb energy levels, the redox state, or Ca^2+^ concentrations, unfolded proteins accumulate triggering the unfolded protein response (UPR) and proteins aggregate, contributing to ERS^96^. Related to the ERS response, AnAc increased the transcript levels of *HSPA5*, *SLC3A2*, *IER3*, *ERRFI1*, *HERPUD1*, *PLIN2*, *MT1X*, *MYC*, *PPP1R15A*, *DDIT3*, *XBP1*, *TRIB3*, *SEC. 24D*, *DUSP1*, *GADD45A*, *AMIGO2*, and *GDF15* in MDA-MB-231 cells (Supplementary Table 7). The UPR inhibits protein translation, induces expression of chaperones, and exports misfolded proteins to the cytosol for degradation. If the UPR fails to relieve the stress, the function of the UPR switches from promoting cell survival to promoting cell death^17,97^, which we speculate may be involved in the observed AnAc-mediated inhibition of cell viability and increased apoptosis^2^.

### AnAc regulated lncRNAs in MDA-MB-231 cells

Data from the Encyclopedia of DNA Elements (ENCODE) Project Consortium indicate that the human genome encodes >28,000 long noncoding RNAs (lncRNAs) that are transcribed by RNA pol II, capped and polyadenylated and expressed in a tissue-specific manner (reviewed in^98^). lncRNAs are involved in regulating numerous biological processes including roles as scaffolds, decoys or signals, cis- and trans- regulation of transcription, antisense interference, imprinting genomic loci, shaping chromosome conformation and allosterically regulating enzymatic activity (reviewed in^99^). Currently, the rate of lncRNA discovery outpaces lncRNA characterization; thus, relatively few lncRNAs are fully characterized. Long intervening noncoding RNAs (lincRNAs) are a subset of lncRNAs and are transcribed from thousands of loci preferentially found within 10 kb of protein-coding genes (reviewed in^100^). lncRNAs are involved in cotranscriptional regulation and act to bridge proteins and chromatin.

AnAc significantly downregulated thirteen and upregulated five lncRNAs in MDA-MB-231 cells (Table 4). These changes are likely due to changes in the entire transcriptome in response to AnAc. MetaCore enrichment analysis only included *MIR24-2*, reflecting the paucity of information included in this database on lncRNAs. The GO Processes and Diseases identified by MetaCore analysis are shown in Supplementary Fig. 9A and B. Only three of the lncRNAs (all downregulated by AnAc) were found in PubMed: *CFLAR-AS1, UBL7-AS1*, and *MIR210HG* (Table 4). Future studies will be needed to address the roles of these lncRNAs in mediating MDA-MB-231 cellular responses to AnAc.

### qPCR validation of selected changes in AnAc-regulated genes

**Figure 8:**
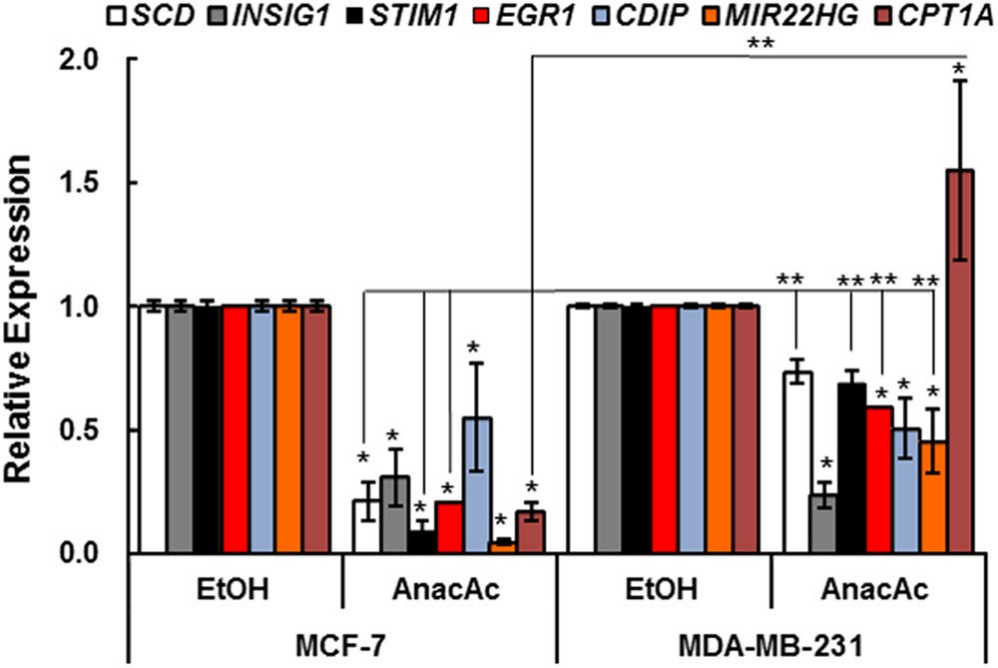
qPCR validation of changes in AnAc target genes. MCF-7 and MDA-MB-231 cells were grown in hormone-depleted medium for 48 h prior to 6 h treatment with vehicle control (ethanol EtOH) 13.5 and 35 µM AnAc, respectively. mRNA expression of the indicated genes relative to GAPDH is shown. Bars are the average of 6 samples from two separate experiments. Statistical evaluation was performed by two way ANOVA followed by Bonferroni post hoc test. *p < 0.05 versus the EtOH vehicle control; ** versus the same gene in MCF-7 AnAc.

We selected *SCD, STIM1, EGR1, CDIP, INSIG1, MIR22HG*, and *CPT1A* for validation by qPCR. Based on the effect of AnAc on each of these genes detected by RNA-seq, we expected *SCD* and *INSIG1* to be decreased in both MCF-7 and MDA-MB-231 cells while *STIM1, EGR1*, and *CDIP* would be inhibited in MCF-7, but not MDA-MB-231 cells and *CPT1A* would be increased in MDA-MB-231 and not significantly regulated in MCF-7 cells. AnAc inhibited *STIM1*, *CDIP*, and *MIR22HG* transcript expression in both cell lines with a greater effect in MCF-7 than MDA-MB-231 cells (Fig. 8). AnAc inhibited *SCD*, *STIM1*, and *CPT1A* in MCF-7 cells, but not in MDA-MB-231 cells. The inhibitory effect of AnAc on *SCR, STIM1, EGR1*, *MIR22HG*, and *CPT1A* was significantly greater in MCF-7 than MDA-MB-231 cells. In fact, AnAc stimulated *CPT1A* in MDA-MB-231 cells. Overall, qPCR results generally confirmed the RNA-seq data except that *EGR1* was inhibited in both MCF-7 and MDA-MB-231 cells.

## Conclusions

The major goal of this study was to identify the global effects of AnAc on the RNA transcriptome of two well-characterized cell lines representing luminal A, ERα+ (MCF-7) and TNBC (MDA-MB-231) breast cancer. We have provided the comprehensive mRNA and lncRNA sets for each cell line and defined their common and cell-specific expression. Notably, AnAc regulated more transcripts in MDA-MB-231 than MCF-7 cells. Only three genes were commonly down- and up-regulated, respectively, by AnAc in both cell lines. The cell-specific and common up- and down-regulated genes were characterized using the MetaCore Gene Ontology (GO) enrichment analysis algorithm. Top among the downregulated enrichment pathways were Development, Unsaturated fatty acid biosynthesis, and Immune response in MCF-7 and Cell cycle and Transcription: ligand-dependent activation of the ESR1/SP pathway in MDA-MB-231 cells, respectively. ERα-dependent and independent pathways are suggested to be involved in the AnAc-mediated transcriptome responses. Top among the upregulated enrichment pathways were Neurophysiological process and Immune response: MIF-JAB1 signaling in MCF-7 cells and PDE4 regulation of cyto/chemokine expression in arthritis and apoptosis and survival: Endoplasmic reticulum stress response pathway in MDA-MB-231 cells. Only one pathway was identified for the three common upregulated genes: Transcription Sirtuin6 regulation and function. qPCR confirmed AnAc regulation of seven genes. Our results suggest that AnAc regulates common and different pathways in ERα+ MCF-7 and MDA-MB-231 TNBC cells.

## Methods

### Materials

AnAc 24:1n5 was purified to greater than 95% as previously reported^2,101^. AnAc 24:1n5 was dissolved in ethanol (EtOH); thus, EtOH was used as a vehicle control.

### Cell culture and treatments

MCF-7 and MDA-MB-231 cells were purchased from American Type Tissue Collection (ATCC, Manassas, VA) and were used within 9 passages from ATCC. MCF-7 and MDA-MB-231 cells were grown as described previously^6^ prior to a 6 h treatment with established IC_50_ concentrations of AnAc 24:1n5: 13.5 µM for MCF-7 and 35.0 µM for MDA-MB-231 cells^2^.

### For mRNA RNA-seq

RNA was isolated from three separate experiments for each cell line and treatment as previously reported^6^. The Truseq Stranded mRNA kit (Illumina) was used to prepare mRNA libraries from 1 µg total RNA. Libraries were confirmed on the Agilent 2100 Bioanalyzer and quantitated using the Illumina Library Quantification Kit, ABI Prism qPCR Mix from Kapa Biosystems and the ABI7900HT real-time PCR instrument at the University of Louisville Center for Genetics and Molecular Medicine (CGeMM) DNA Sequencing Core Facility. 76 cycle single read sequencing was performed with the 500 High-output v2 (75cycle) sequencing kit on the Illumina NextSeq. 500 instrument. The sequence reads were mapped to the human reference genome, version GRCh37.1 (hg19) using the mapping algorithm tophat^11^ version 2.0.2. The expression levels were quantified at loci specified by the annotation found within the ENSEMBL release 73 gene description file (Homo_sapiens.GRCh37.73.gtf) using cufflinks version 2.2.1. Contributions to the annotation file from both ribosomal RNA (rRNA) and mitochondrial RNA (mtRNA) were removed from the gtf file prior to use. Differential analyses between the specified conditions was performed using cuffdiff version 2.2.1. The raw data were uploaded in the Gene Expression Omnibus (GEO) database as GSE78011.

### Differential Gene Expression Analysis

The analysis was similar to that used to identify miRNAs regulated by AnAc in these cell lines^6^ and that data analysis pipeline is shown in Supplementary Figure 10. The number of raw reads, number of reads after trimming, and number of reads successfully aligned for each of the samples is provided in Supplementary Table 8. Aligned RNA-seq reads were assembled according to the hg19.gtf annotation file (downloaded from ENSEMBL^102^) using Cufflinks (version 2.2.1)^11^. For each comparison, both cufflinks assemblies were merged, and the resulting merged gtf file serves as the transcript input for differential gene expression analysis in Gene Ontology and KEGG pathways (below). For three of the comparisons, a p-value cutoff ≤0.05 was used to determine differential expression. For the MCF-7 *vs*. MDA-MB-231 comparison, differential genes were determined using a q-value of ≤0.01 and a |FC| ≥2. Differentially expressed genes (DEGs) for each comparison were used for further analysis of enriched Gene Ontology Biological Processes (GO:BP)^12,13^ and KEGG Pathways^14^ using categoryCompare^15^. The Entrez gene ID for each DEG was obtained from the human Entrez IDs database downloaded from NCBI. Tables for the enriched GO:BP and KEGG pathways were generated as text files from four lists of DEGs with a unique Entrez gene ID.

### *In silico* MetaCore network analysis

Pathway and network analysis of differentially expressed genes was performed in MetaCore version 6.27 (GeneGO, Thomson Reuters, New York, N.Y.)^103^.

### Luciferase assay

To analyze NFκB activity, MCF-7 cells were transiently transfected with pGL4.32[luc2P/NF-κB-RE/Hygro] (Promega, Madison, WI) containing five copies of a NFκB response element and pGL4-hRluc-TK (Renilla, Promega) for 48 h and treated with 10 ng/ml TNFα and 0–40 µM AnAc 24:1n5 for 6 h before performing dual luciferase assay (Promega). Firefly luciferase was normalized by *Renilla* luciferase. Values are the average of three separate wells in one experiment ± SEM.

RNA isolation, RT-PCR and quantitative real-time PCR (qPCR) was performed essentially as described previously in MCF-7 and MDA-MB-231 cells treated with vehicle control (EtOH) or AnAc 24:1n5 (13.5 and 35 µM, respectively) for 6 h^6^. PCR Primers were synthesized by Integrated DNA Technologies (Coralville, IA) and sequences used were are listed in Supplementary Table 9. *GAPDH* was used as a reference for normalization^104^. qPCR was performed in triplicate using ABI Viia 7 (LifeTechnologies). Fold change relative to vehicle-treated, control cells was estimated by the comparative threshold cycle (Ct) method (2^−ΔΔCT^)^105^.

### Data availability statement

Raw sequencing data files obtained from our analysis are available at GEO: accession number GSE78011. All data analyzed during this study are included in this published article (and its Supplementary Information files).

## Electronic supplementary material

Supplementary Information
