# Supplementary material for: Transcriptomic response of breast cancer cells to anacardic acid
Source: Sci Rep. 2018 May 23;8:8063. doi: 10.1038/s41598-018-26429-x (PMC5966448; doi:10.1038/s41598-018-26429-x)
Supplement: Supplementary file 1 — Supplementary Information [file 41598_2018_26429_MOESM1_ESM.pdf]

Supplementary Tables (9) and Figures (10)

Transcriptomic response of breast cancer cells to anacardic acid

David J. Schultz, Abirami Krishna, Stephany Vittitow, Negin Alizadeh-Rad, Penn Muluhngwi, Eric C. Rouchka, and Carolyn M. Klinge

List of files:

Supplementary Tables 1-9

Supplementary Table 1: Top five enriched GO:BP terms for DEGs from MCF-7 AnAc and MCF-7 control vs. MDA-MB-231 AnAc and MDA-MB-231 control using CategoryCompare.

Supplementary Table 2: Enriched GO:BP terms for DEGs from MCF-7 AnAc vs. control and top five enriched GO:BP terms for DEGs in MDA-MB-231 AnAc vs. control using CategoryCompare.

Supplementary Table 3: Enriched KEGG pathway from MCF-7 AnActrt vs. control and MDA-MB-231 AnAc vs. control using CategoryCompare

Supplementary Table 4: Genes downregulated by AnAc in MDA-MB-231 breast cancer cells.

Supplementary Table 5: Genes upregulated by AnAc in MDA-MB-231 breast cancer cells.

Supplementary Table 6: Top 18 genes uniquely inhibited by anacardic acid in MDA-MB-231 cells after 6 h treatment with 35 µM.

Supplementary Table 7: Genes uniquely upregulated in MDA-MB-231 cells after 6 h. of 35 µM AnAc treatment.

Supplementary Table 8: Summary of sequence analysis

Supplementary Table 9: Primers used for qPCR

Supplementary Figures 1-10

Supplementary Figure 1: MetaCore *GO Processes*

Supplementary Figure 3: MetaCore network analysis of DEG in MCF-7 cells treated with AnAc – 1 step

Supplementary Figure 4: MetaCore top network for AnAc-downregulated genes in MCF-7 cells generated by MetaCore

Supplementary Figure 5: Transcription factor regulation of DEGs in AnAc- treated MCF-7 cells generated by MetaCore

Supplementary Figure 6: Shortest paths with 1 step generated by MetaCore for genes downregulated by AnAc in MCF-7 cells

Supplementary Figure 7: Shown is the shortest paths analysis with 1 step generated by MetaCore for genes upregulated by AnAc in MCF-7 cells

Supplementary Figure 8: Shown is the shortest paths analysis with 2 steps generated by MetaCore for genes upregulated by AnAc in MCF-7 cells.

Supplementary Figure 9: These are Gene Ontology (GO) cellular processes (A) and diseases (B) and the Network (5 steps) identified by MetaCore by enrichment analysis of the lncRNAs regulated by AnAc in MDA-MB-231 cells (Table 9).

Supplementary Figure 10: Data analysis pipeline using command line

Supplementary Table 1: Top five enriched GO:BP terms for DEGs from MCF-7 AnAc and MCF-7 control *vs.* MDA-MB-231 AnAc and MDA-MB-231 control using CategoryCompare. These GO terms suggest differences between MCF-7 and MDA-MB-231 cells.

GO term	Description	gene #	P value
GO:0007156	Homophilic cell adhesion	78	1.27E-10
GO:0051056	Regulation of small GTPase mediated signal transduction	203	2.30E-10
GO:0043087	Regulation of GTPase activity	210	8.84E-10
GO:0022612	Gland morphogenesis	65	2.15E-09
GO:0043547	Positive regulation of GTPase activity	196	2.26E-09

Supplementary Table 2: Enriched GO:BP terms for DEGs from MCF-7 AnAc *vs.* control and top five enriched GO:BP terms for DEGs in MDA-MB-231 AnAc *vs.* control using CategoryCompare. These GO terms suggest AnAc elicits different responses in these two breast cancer cell lines.

Control *vs.* AnAc	GO term (description)	gene #	P value
MCF-7	GO:0071229 (cell. response to acid chem.)	4	0.00072
MDA-MB-231	GO:0034976 (response to ER stress)	27	7.22E-11
MDA-MB-231	GO:0006695 (cholesterol biosyn. process)	15	1.45E-10
MDA-MB-231	GO:0016126 (sterol biosyn. process)	15	8.16E-10
MDA-MB-231	GO:0006986 (response to unfolded protein)	24	1.62E-09
MDA-MB-231	GO:0030968 (ER unfolded protein response)	19	2.66E-09

Supplementary Table 3: Enriched KEGG pathways from MCF-7 AnAc *vs.* control and MDA-MB-231 AnAc *vs.* control using CategoryCompare. biosyn = biosynthesis

Control *vs.* AnAc	KEGG term (description)	Gene #	P value
MCF-7	5322 (systemic lupus erythematosus)	20	5.45E-24
MDA-MB-231	0100 (steroid biosyn.)	8	1.13E-06
MDA-MB-231	5215 (prostate cancer)	13	3.0E-4
MDA-MB-231	4115 (p53 signaling pathway)	11	3.1E-4
MDA-MB-231	0532 (glycosaminoglycan biosyn.-chondroitin sulfate)	6	4.3E-4
MDA-MB-231	0900 (terpenoid backbone biosyn.)	5	4.7E-4

Supplementary Table 4: Genes downregulated by AnAc in MDA-MB-231 breast cancer cells. MDA-MB-231 cells were grown in hormone-depleted medium prior to 6 h treatment with 35 µM AnAc.

ENSEMBL GENE	Gene	MDA_ctrl	MDA_AnAc	log2FC (MDA_AnAc /MDA_ctrl)	p_value
ENSG00000227682	ATP5A1P2	1.01217	0	-7.2969	0.02005
ENSG00000265095	FTLP12	0.721445	0	-7.2969	0.0001
ENSG00000164972	C9orf24	0.578662	0	-7.2969	0.00035
ENSG00000253409	TRBV7-4	0.445133	0	-7.2969	0.02005
ENSG00000214374	RPLP2P1	0.397048	0	-7.2969	0.02905
ENSG00000117425	PTCH2	9.15899	0.155354	-5.88156	5.00E-05
ENSG00000255082	GRM5-AS1	0.810723	0.0462224	-4.13255	0.0371
ENSG00000243069	ARHGEF26-AS1	1.70296	0.17091	-3.31673	0.0232
ENSG00000175003	SLC22A1	0.821053	0.0871177	-3.23644	0.0016
ENSG00000253981	ALG1L13P	0.576739	0.0671003	-3.10353	0.015
ENSG00000226312	CFLAR-AS1	0.997156	0.126716	-2.97622	0.00445
ENSG00000164037	SLC9B1	0.409842	0.0557936	-2.87689	0.0071
ENSG00000160219	GAB3	0.374295	0.0516503	-2.85733	0.0188
ENSG00000080947	CROCCP3	0.658834	0.109159	-2.59349	0.025
ENSG00000196196	HRCT1	0.636227	0.124505	-2.35334	0.02735
ENSG00000136425	CIB2	0.458355	0.0972356	-2.23691	0.0303
ENSG00000010310	GIPR	1.09821	0.249149	-2.14007	0.0048
ENSG00000173338	KCNK7	0.81508	0.19151	-2.08953	0.01855
ENSG00000187994	RINL	0.983732	0.234154	-2.07081	0.0396
ENSG00000188549	C15orf52	65.0323	15.5922	-2.06033	0.04
ENSG00000206140	TMEM191C	1.09296	0.279869	-1.96542	0.00055
ENSG00000240021///ENSG00000213057///ENSG00000184909	TEX35	0.449311	0.120052	-1.90405	0.00185
ENSG00000101670	LIPG	4.05963	1.10967	-1.87122	5.00E-05
ENSG00000120049	KCNIP2	0.413398	0.113734	-1.86187	0.00295
ENSG00000260781	ARHGAP23P1	0.374862	0.10681	-1.81132	0.02245
ENSG00000137440	FGFBP1	0.413941	0.119193	-1.79613	0.03235
ENSG00000171798	KNDC1	0.485858	0.143453	-1.75996	0.00815
ENSG00000181085	MAPK15	0.741469	0.221906	-1.74044	0.00275
ENSG00000127957	PMS2P3	2.4767	0.746104	-1.73097	0.0193
ENSG00000169851	PCDH7	4.53208	1.50944	-1.58616	0.0038
ENSG00000143867	OSR1	7.19085	2.41984	-1.57125	5.00E-05
ENSG00000186480	INSIG1	114.522	39.6346	-1.53079	5.00E-05
ENSG00000127129	EDN2	5.03275	1.75244	-1.52198	5.00E-05

ENSG00000062282	DGAT2	6.86483	2.43933	-1.49274	0.0028
ENSG00000099810///<br>ENSG00000264545	MTAP	0.652794	0.232091	-1.49194	5.00E-05
ENSG00000210135	MT-TN	2089.08	746.329	-1.48498	0.00085
ENSG00000066056	TIE1	2.35319	0.869109	-1.43701	0.0116
ENSG00000233101	HOXB-AS3	0.392222	0.147338	-1.41254	0.02345
ENSG00000173930	SLCO4C1	0.702222	0.266641	-1.39703	0.0003
ENSG00000198574	SH2D1B	0.635187	0.246508	-1.36555	0.00275
ENSG00000012223	LTF	0.534282	0.210294	-1.34519	0.03415
ENSG00000130164	LDLR	173.557	68.7098	-1.33682	5.00E-05
ENSG00000247240	UBL7-AS1	2.00353	0.802718	-1.31958	0.0112
ENSG00000130045	NXNL2	0.392139	0.15721	-1.31868	0.03745
ENSG00000125965	GDF5	1.9726	0.819815	-1.26673	5.00E-05
ENSG00000169174	PCSK9	52.2434	21.7172	-1.26641	5.00E-05
ENSG00000052802	MSMO1	161.865	68.7072	-1.23626	5.00E-05
ENSG00000165388	ZNF488	2.75824	1.17449	-1.23171	5.00E-05
ENSG00000133624	ZNF767	1.71912	0.748641	-1.19933	0.00485
ENSG00000125378	BMP4	145.799	63.6598	-1.19552	5.00E-05
ENSG00000259070	LINC00639	0.46453	0.20404	-1.18692	0.0274
ENSG00000128536	CDHR3	1.21083	0.53551	-1.17701	0.0183
ENSG00000168874	ATOH8	0.717217	0.32177	-1.15638	0.0064
ENSG00000144134	RABL2A	1.61075	0.723276	-1.15511	0.0067
ENSG00000125657	TNFSF9	6.69557	3.06582	-1.12693	5.00E-05
ENSG00000247095	MIR210HG	14.9862	6.99695	-1.09884	0.0005
ENSG00000068781	STON1-<br>GTF2A1L	0.714413	0.335396	-1.09089	0.02385
ENSG00000197816	CCDC180	6.49356	3.08414	-1.07414	0.005
ENSG00000165300	SLITRK5	0.769602	0.366814	-1.06907	0.0001
ENSG00000095752	IL11	57.0761	27.2148	-1.06849	5.00E-05
ENSG00000183607	GKN2	1.54229	0.739009	-1.06141	0.01425
ENSG00000181544	FANCB	3.73472	1.80045	-1.05264	0.0076
ENSG00000181577	C6orf223	1.3176	0.643293	-1.03437	0.00105
ENSG00000189350	FAM179A	1.44784	0.714045	-1.01982	0.0206
ENSG00000143494	VASH2	1.40135	0.693218	-1.01543	0.0299
ENSG00000196418	ZNF124	4.20176	2.0841	-1.01157	0.00655
ENSG00000104140	RHOV	1.28164	0.650075	-0.979317	0.0039
ENSG00000189184	PCDH18	0.74273	0.379004	-0.970625	0.01505
ENSG00000189057	FAM111B	15.6497	8.01749	-0.964909	5.00E-05
ENSG00000170379	FAM115C	0.691659	0.355043	-0.962069	0.0018
ENSG00000227195	MIR663A	0.859964	0.44336	-0.955797	0.0286
ENSG00000164045	CDC25A	14.8524	7.66873	-0.953639	5.00E-05
ENSG00000105605	CACNG7	2.3927	1.23795	-0.950682	0.02035

ENSG00000125872	LRRN4	0.431455	0.223712	-0.947569	0.0168
ENSG00000163083	INHBB	15.1299	7.85008	-0.946622	5.00E-05
ENSG00000171408	PDE7B	1.65795	0.863145	-0.941728	0.0009
ENSG00000187244	BCAM	9.25063	4.82536	-0.938915	5.00E-05
ENSG00000165507	C10orf10	4.63801	2.44689	-0.922557	0.0041
ENSG00000122863	CHST3	14.8508	7.88712	-0.91297	5.00E-05
ENSG00000158246	FAM46B	4.5257	2.41127	-0.908349	5.00E-05
ENSG00000157193	LRP8	32.4757	17.4643	-0.894954	0.00075
ENSG00000158458	NRG2	1.95832	1.06855	-0.873964	0.0122
ENSG00000187566	NHLRC1	1.12235	0.613355	-0.871729	0.0052
ENSG00000146950	SHROOM2	2.1876	1.19817	-0.868516	0.0003
ENSG00000163701	IL17RE	8.47736	4.6532	-0.865391	0.00085
ENSG00000117461	PIK3R3	3.37731	1.87098	-0.852079	0.0004
ENSG00000007968	E2F2	1.71895	0.954074	-0.849358	0.00015
ENSG00000147119	CHST7	4.1212	2.29237	-0.846224	5.00E-05
ENSG00000152291	TGOLN2	35.4383	19.7857	-0.840854	5.00E-05
ENSG00000145632	PLK2	233.484	130.539	-0.838851	0.0237
ENSG00000171992	SYNPO	4.27327	2.39683	-0.834215	0.019
ENSG00000184588	PDE4B	1.16262	0.658491	-0.820148	0.01745
ENSG00000121858	TNFSF10	21.0923	11.9514	-0.819538	5.00E-05
ENSG00000133874	RNF122	1.47604	0.836422	-0.819431	0.00485
ENSG00000170577	SIX2	5.72678	3.24606	-0.819035	5.00E-05
ENSG00000171451	DSEL	0.703282	0.400615	-0.811889	0.00265
ENSG00000183426	NPIPA1	1.79111	1.02165	-0.809952	0.0187
ENSG00000099194	SCD	358.989	204.864	-0.809276	5.00E-05
ENSG00000169418	NPR1	0.765861	0.438847	-0.803366	0.01755
ENSG00000176092	AIM1L	2.33421	1.35101	-0.7889	0.04685
ENSG00000054967	RELT	3.87021	2.24316	-0.786876	0.0056
ENSG00000140451	PIF1	7.10517	4.14134	-0.778771	0.02835
ENSG00000196110	ZNF699	4.80647	2.82189	-0.768318	0.0096
ENSG00000184574	LPAR5	0.5851	0.344487	-0.764233	0.02295
ENSG00000148926	ADM	46.9353	27.7699	-0.757149	0.00015
ENSG00000132386	SERPINF1	1.42954	0.845855	-0.757072	0.048
ENSG00000155034	FBXL18	3.76393	2.2293	-0.755651	0.0146
ENSG00000129173	E2F8	6.13297	3.63657	-0.754007	0.0002
ENSG00000100065	CARD10	20.6778	12.2717	-0.752751	0.0046
ENSG00000134851	TMEM165	84.7055	50.3243	-0.751201	5.00E-05
ENSG00000233493	TMEM238	1.80949	1.07817	-0.747005	0.0255
ENSG00000142279	WTIP	20.404	12.1694	-0.745598	0.0023
ENSG00000099251	HSD17B7P2	1.24992	0.746907	-0.742833	0.0228
ENSG00000139438	FAM222A	2.18754	1.31241	-0.737089	0.00315

ENSG00000170801	HTRA3	2.30302	1.38399	-0.734694	0.0017
ENSG00000168497	SDPR	23.6906	14.2682	-0.731508	5.00E-05
ENSG00000109084	TMEM97	37.8085	22.7842	-0.730675	0.00705
ENSG00000031003	FAM13B	10.4019	6.27593	-0.728948	0.0001
ENSG00000067836	ROGDI	6.65686	4.01681	-0.728792	0.03995
ENSG00000267080	ASB16-AS1	4.35952	2.63269	-0.727632	0.03275
ENSG00000176406	RIMS2	2.03304	1.22783	-0.727527	0.0498
ENSG00000175305	CCNE2	18.7471	11.3349	-0.725898	0.0201
ENSG00000106003	LFNG	11.6485	7.05589	-0.72325	5.00E-05
ENSG00000145882	PCYOX1L	5.19718	3.15144	-0.721716	0.00045
ENSG00000164761	TNFRSF11B	4.29734	2.60845	-0.720252	0.0073
ENSG00000006638	TBXA2R	1.3359	0.811556	-0.719044	0.04685
ENSG00000205476	CCDC85C	31.1205	18.9304	-0.717159	0.04135
ENSG00000226943	ALG1L5P	2.98741	1.83437	-0.703611	0.02865
ENSG00000164211	STARD4	27.7474	17.0422	-0.703239	0.02495
ENSG00000109929	SC5D	81.0862	50.0496	-0.696098	0.0461
ENSG00000184254	ALDH1A3	20.8685	12.9009	-0.693847	0.0403
ENSG00000114019	AMOTL2	32.5065	20.1039	-0.693253	5.00E-05
ENSG00000176248	ANAPC2	17.6265	10.909	-0.69223	0.015
ENSG00000180739	S1PR5	1.08534	0.671778	-0.692096	0.0206
ENSG00000234769	WASH4P	1.45318	0.900706	-0.690085	0.006
ENSG00000168646///ENSG00000154240///ENSG00000266076	AXIN2	5.71753	3.54554	-0.689387	0.02515
ENSG00000228486	LINC01125	1.33461	0.829433	-0.686219	0.02265
ENSG00000204516	MICB	46.455	29.035	-0.67804	5.00E-05
ENSG00000160285///ENSG00000235878	LSS	50.0053	31.3738	-0.67252	0.02665
ENSG00000092470	WDR76	13.8438	8.68831	-0.672089	0.00295
ENSG00000145604	SKP2	16.605	10.437	-0.669901	0.0382
ENSG00000105519///ENSG00000187650///ENSG00000267314	CAPS	1.58735	0.998345	-0.669009	0.0468
ENSG00000162062	C16orf59	8.38423	5.27917	-0.667367	0.01145
ENSG00000164976	KIAA1161	1.50099	0.948215	-0.662627	0.00475
ENSG00000099849	RASSF7	35.7954	22.6607	-0.659581	0.00665
ENSG00000162073	PAQR4	8.51016	5.3944	-0.657723	0.03955
ENSG00000162878	PKDCC	4.78557	3.03443	-0.657267	0.02185
ENSG00000162512	SDC3	17.4861	11.0926	-0.656612	5.00E-05
ENSG00000144354	CDCA7	10.368	6.57849	-0.656316	0.00045
ENSG00000258701	LINC00638	0.740618	0.470402	-0.654836	0.0374
ENSG00000142235	LMTK3	0.427163	0.272987	-0.645956	0.02885

ENSG00000163026	C2orf44	5.87022	3.77432	-0.637198	0.00135
ENSG00000143772	ITPKB	0.82493	0.531056	-0.635408	0.0089
ENSG00000183779	ZNF703	7.47505	4.82183	-0.632504	0.0003
ENSG00000267519	MIR24-2	6.31835	4.07702	-0.63203	0.00425
ENSG00000065328	MCM10	13.222	8.54909	-0.629096	0.00275
ENSG00000092969	TGFB2	53.2601	34.7987	-0.614023	0.0122
ENSG00000177842	ZNF620	3.68808	2.41229	-0.612463	0.03315
ENSG00000114315	HES1	4.25226	2.78551	-0.610289	0.00555
ENSG00000154734	ADAMTS1	23.8236	15.63	-0.60807	0.0006
ENSG00000111981	ULBP1	0.544825	0.357883	-0.606305	0.0493
ENSG00000172123	SLFN12	11.9801	7.87188	-0.605854	0.00765
ENSG00000166166	TRMT61A	10.5705	6.9625	-0.602365	0.00035
ENSG00000105717	PBX4	4.87941	3.22003	-0.599631	0.0242
ENSG00000109689	STIM2	10.7553	7.14527	-0.589983	0.04085
ENSG00000174669	SLC29A2	10.9652	7.30004	-0.586962	0.00065
ENSG00000147383	NSDHL	56.1151	37.4034	-0.585221	0.00025
ENSG00000225756	DBH-AS1	1.16735	0.778693	-0.584106	0.04765
ENSG00000197530	MIB2	14.8325	9.89452	-0.584061	0.0456
ENSG00000034063	UHRF1	22.0197	14.8172	-0.571525	0.00065
ENSG00000001630///<br>ENSG00000240720	CYP51A1	92.1557	62.1014	-0.569446	0.00305
ENSG00000183655	KLHL25	2.40145	1.61919	-0.568639	0.0365
ENSG00000131061	ZNF341	1.91798	1.29609	-0.56542	0.0157
ENSG00000241360///<br>ENSG00000100092	PDXP	23.1317	15.6855	-0.560444	0.0013
ENSG00000085063///<br>ENSG00000205177	CD59	509.267	345.553	-0.559514	0.0123
ENSG00000167767	KRT80	29.5954	20.0962	-0.558451	0.00035
ENSG00000135114	OASL	10.1833	6.91763	-0.557859	0.00255
ENSG00000173894	CBX2	4.88068	3.31619	-0.557556	0.01145
ENSG00000149930	TAOK2	7.78207	5.29229	-0.556262	0.00215
ENSG00000070413	DGCR2	11.6492	7.93919	-0.553167	0.00995
ENSG00000128294	TPST2	20.922	14.3016	-0.548844	0.00095
ENSG00000149541	B3GAT3	36.3181	24.8326	-0.54845	0.00495
ENSG00000174775	HRAS	196.688	134.524	-0.548045	0.04655
ENSG00000127663	KDM4B	6.87352	4.71192	-0.544733	0.04875
ENSG00000188827	SLX4	1.33261	0.914868	-0.54262	0.00655
ENSG00000124104	SNX21	19.5648	13.4337	-0.5424	0.04245
ENSG00000170542	SERPINB9	14.1113	9.70024	-0.54076	0.0013
ENSG00000158169	FANCC	7.35897	5.05944	-0.540526	0.04965
ENSG00000131019	ULBP3	4.83252	3.32263	-0.540447	0.0455
ENSG00000183508	FAM46C	5.63156	3.87388	-0.539755	0.0018

ENSG00000178234	GALNT11	23.4387	16.1706	-0.535517	0.00605
ENSG00000125968	ID1	15.2311	10.5087	-0.535437	0.00495
ENSG00000170745	KCNS3	4.97618	3.43887	-0.533102	0.0053
ENSG00000142627	EPHA2	144.592	100.164	-0.529622	0.0061
ENSG00000187908	DMBT1	2.97489	2.06349	-0.527754	0.008
ENSG00000175793	SFN	120.141	83.4401	-0.525911	0.0009
ENSG00000169129	AFAP1L2	7.92092	5.50579	-0.524718	0.0027
ENSG00000175040	CHST2	4.27025	2.98239	-0.51785	0.00475
ENSG00000131944	C19orf40	6.41733	4.48267	-0.517616	0.03775
ENSG00000130193	THEM6	24.6895	17.2735	-0.51534	0.00185
ENSG00000151413	NUBPL	8.88083	6.21433	-0.515095	0.04955
ENSG00000183421	RIPK4	3.32894	2.3346	-0.511885	0.0059
ENSG00000180834	MAP6D1	5.45255	3.82926	-0.509868	0.01005
ENSG00000108963	DPH1	31.5378	22.161	-0.509054	0.0325
ENSG00000164056	SPRY1	18.063	12.6953	-0.508735	0.0089
ENSG00000187210	GCNT1	10.3563	7.29296	-0.505937	0.0359
ENSG00000136720	HS6ST1	14.9226	10.5096	-0.505795	0.0013
ENSG00000159792	PSKH1	9.20227	6.492	-0.503325	0.02945
ENSG00000172197	MBOAT1	3.31742	2.34262	-0.50194	0.0106
ENSG00000182541	LIMK2	4.96594	3.5086	-0.501169	0.0424
ENSG00000213672	NCKIPSD	20.5488	14.5322	-0.499802	0.04025
ENSG00000196247	ZNF107	0.880795	0.623263	-0.498966	0.03715
ENSG00000177427	MIEF2	8.22972	5.84054	-0.494742	0.0347
ENSG00000133317	LGALS12	3.71863	2.65149	-0.487965	0.0294
ENSG00000070614	NDST1	21.2156	15.1342	-0.487311	0.0102
ENSG00000160193	WDR4	6.89268	4.92503	-0.484933	0.0251
ENSG00000204682	CASC10	2.14582	1.53553	-0.482795	0.0177
ENSG00000169213	RAB3B	0.556598	0.398397	-0.482429	0.0208
ENSG00000212916	MAP10	1.26994	0.910726	-0.479668	0.02435
ENSG00000179403	VWA1	20.6909	14.8565	-0.477901	0.0428
ENSG00000186283	TOR3A	29.5867	21.2746	-0.475817	0.016
ENSG00000243449	C4orf48	20.6027	14.8272	-0.47459	0.03395
ENSG00000196584	XRCC2	3.4213	2.46498	-0.472966	0.00915
ENSG00000198934	MAGEE1	1.15071	0.830342	-0.470751	0.0484
ENSG00000162063	CCNF	10.6928	7.71727	-0.470479	0.0049
ENSG00000136856	SLC2A8	7.71924	5.57216	-0.47022	0.03995
ENSG00000106077	ABHD11	12.2554	8.86119	-0.46785	0.04975
ENSG00000255198	SNHG9	57.0177	41.235	-0.467538	0.03965
ENSG00000110092	CCND1	179.335	129.846	-0.465857	0.00965
ENSG00000188070	C11orf95	7.55303	5.47305	-0.46471	0.0138
ENSG00000184992	BRI3BP	6.87329	4.98705	-0.462814	0.0054

ENSG00000150687	PRSS23	444.051	322.215	-0.462703	0.02165
ENSG00000124541	RRP36	132.075	95.957	-0.460894	0.0029
ENSG00000166106	ADAMTS15	13.2995	9.67794	-0.458598	0.0039
ENSG00000143816	WNT9A	1.82275	1.32642	-0.458582	0.0383
ENSG00000137040	RANBP6	4.78422	3.48273	-0.458065	0.0124
ENSG00000147576///<br>ENSG00000169085///<br>ENSG00000179041	ADHFE1	22.8259	16.6245	-0.457359	0.00675
ENSG00000157240	FZD1	6.59584	4.80835	-0.456015	0.0085
ENSG00000099308	MAST3	2.05406	1.50225	-0.451353	0.0468
ENSG00000133069	TMCC2	3.51782	2.57427	-0.450517	0.02345
ENSG00000169105	CHST14	11.5742	8.47067	-0.45037	0.01
ENSG00000138623	SEMA7A	32.501	23.8428	-0.446929	0.0083
ENSG00000077454///<br>ENSG00000205307	LRCH4	19.1194	14.064	-0.443028	0.03865
ENSG00000132669	RIN2	17.4782	12.8577	-0.442923	0.00805
ENSG00000106009	BRAT1	17.1104	12.614	-0.439847	0.0331
ENSG00000109576	AADAT	5.67023	4.18565	-0.437957	0.02845
ENSG00000138131	LOXL4	37.5726	27.772	-0.43605	0.00495
ENSG00000171448	ZBTB26	1.61039	1.19102	-0.435217	0.0415
ENSG00000102034	ELF4	22.6166	16.7694	-0.431551	0.0057
ENSG00000160326	SLC2A6	6.67123	4.95153	-0.430078	0.0183
ENSG00000131779	PEX11B	13.0928	9.7248	-0.429037	0.04735
ENSG00000099812	MISP	2.84337	2.12359	-0.421099	0.0375
ENSG00000158715	SLC45A3	3.70556	2.77326	-0.418107	0.03635
ENSG00000112576	CCND3	140.897	105.469	-0.41782	0.01895
ENSG00000188486	H2AFX	78.8196	59.0084	-0.417635	0.00775
ENSG00000100075	SLC25A1	54.3263	40.7099	-0.416271	0.0091
ENSG00000121236///<br>ENSG00000258588///<br>ENSG00000258659	TRIM6	18.407	13.8199	-0.413505	0.04705
ENSG00000064300	NGFR	2.79656	2.10154	-0.412205	0.03645
ENSG00000100034	PPM1F	18.4796	13.891	-0.411782	0.01805
ENSG00000139546	TARBP2	25.7515	19.3585	-0.411695	0.02265
ENSG00000139645	ANKRD52	8.81643	6.62981	-0.411226	0.02545
ENSG00000204520	MICA	75.2617	56.6066	-0.410947	0.01055
ENSG00000170498	KISS1	300.68	226.369	-0.409554	0.00855
ENSG00000184557	SOCS3	22.2287	16.7452	-0.408679	0.01055
ENSG00000186185	KIF18B	13.7183	10.3384	-0.40808	0.0118
ENSG00000136982	DSCC1	14.0372	10.6288	-0.401283	0.01725
ENSG00000165802	NSMF	40.6084	30.7787	-0.399846	0.03235
ENSG00000172428	MYEOV2	62.9608	47.728	-0.399618	0.02255

ENSG00000153391///<br>ENSG00000267140	INO80C	82.6562	62.6816	-0.399081	0.044
ENSG00000126709	IFI6	14.5799	11.0758	-0.396577	0.04375
ENSG00000178409	BEND3	4.20852	3.19977	-0.395342	0.02085
ENSG00000125266	EFNB2	10.3783	7.89734	-0.394128	0.0184
ENSG00000115884	SDC1	38.6848	29.49	-0.39154	0.0323
ENSG00000132819	RBM38	13.8724	10.5899	-0.38952	0.0353
ENSG00000183741	CBX6	10.6343	8.12435	-0.388406	0.0155
ENSG00000187123	LYPD6	4.65298	3.56026	-0.386174	0.03745
ENSG00000171161	ZNF672	11.3229	8.67595	-0.38415	0.0339
ENSG00000114383///<br>ENSG00000068001	TUSC2	56.9465	43.6749	-0.382803	0.02295
ENSG00000148334	PTGES2	42.0741	32.2847	-0.382081	0.04705
ENSG00000115526	CHST10	15.0575	11.573	-0.379716	0.03045
ENSG00000060491	OGFR	42.3422	32.5787	-0.378167	0.0224
ENSG00000110328	GALNT18	7.95326	6.12095	-0.37779	0.0354
ENSG00000116685	KIAA2013	27.248	20.9727	-0.377639	0.01555
ENSG00000178567	EPM2AIP1	2.30261	1.77306	-0.377032	0.0365
ENSG00000141314///<br>ENSG00000010244	RHBDL3	95.1033	73.4328	-0.373071	0.03745
ENSG00000112312	GMNN	150.269	116.202	-0.370921	0.02485
ENSG00000198959	TGM2	75.3926	58.4489	-0.367248	0.01495
ENSG00000148384	INPP5E	5.98537	4.64023	-0.367243	0.0403
ENSG00000149177	PTPRJ	11.8808	9.25384	-0.36051	0.03
ENSG00000198890	PRMT6	10.5977	8.26052	-0.359454	0.03565
ENSG00000169258	GPRIN1	4.0788	3.17931	-0.35943	0.04835
ENSG00000124145	SDC4	68.8093	53.6628	-0.358681	0.03805
ENSG00000149218	ENDOD1	15.6069	12.1762	-0.358124	0.02355
ENSG00000198695	MT-ND6	709.886	554.798	-0.355626	0.0206
ENSG00000175970	UNC119B	11.4484	8.94807	-0.355501	0.04415
ENSG00000150990	DHX37	9.46615	7.40127	-0.355006	0.03325
ENSG00000088356	PDRG1	43.2186	33.8032	-0.354493	0.0299
ENSG00000075218	GTSE1	27.5686	21.5733	-0.353778	0.0414
ENSG00000104998	IL27RA	7.64905	5.98683	-0.353489	0.0465
ENSG00000175701	LINC00116	70.4213	55.2165	-0.350913	0.04765
ENSG00000156802	ATAD2	35.6715	27.9955	-0.349574	0.02465
ENSG00000186834	HEXIM1	23.3117	18.2974	-0.349417	0.03915
ENSG00000101447	FAM83D	39.2941	30.8678	-0.348211	0.0255
ENSG00000074266	EED	27.6479	21.7299	-0.347489	0.03565
ENSG00000164430	MB21D1	17.9892	14.1569	-0.345623	0.0317
ENSG00000197019	SERTAD1	15.6249	12.3141	-0.343539	0.04185
ENSG00000124496	TRERF1	13.0566	10.3014	-0.341938	0.0379

ENSG00000171345	KRT19	509.235	403.012	-0.337506	0.0409
ENSG00000131873	CHSY1	14.242	11.2812	-0.336234	0.04175
ENSG00000162783	IER5	43.9163	34.7944	-0.335903	0.0335
ENSG00000137166	FOXP4	25.1236	19.912	-0.335404	0.03635
ENSG00000101216	GMEB2	6.86472	5.44344	-0.334684	0.0485
ENSG00000198728	LDB1	18.3352	14.5605	-0.332563	0.0439
ENSG00000070047	PHRF1	8.60909	6.84284	-0.331267	0.0428
ENSG00000160949	TONSL	10.1383	8.08273	-0.326899	0.047
ENSG00000249992	TMEM158	26.7121	21.3981	-0.320009	0.0496
ENSG00000124664	SPDEF	46.9362	37.6424	-0.318341	0.04495
ENSG00000101412	E2F1	36.7193	29.4606	-0.317749	0.04435
ENSG00000134690	CDCA8	56.2885	45.2998	-0.313335	0.0432
ENSG00000141753	IGFBP4	609.843	491.278	-0.311898	0.0498

SupplementaryTable 5: Genes upregulated by AnAc in MDA-MB-231 breast cancer cells. MDA-MB-231 cells were grown in hormone-depleted medium prior to 6 h treatment with 35 µM AnAc.

ENSEMBL GENE	Gene	MDA_ctrl	MDA_AnAc	log2FC (MDA_AnAc/MDA_ctrl)	p_value
ENSG00000223298	RNY3P8	0	16.3345	7.34514	4.45E-03
ENSG00000145975	FAM217A	0	0.600164	7.34514	0.011
ENSG00000214578	HMGN2P15	0	0.654405	7.34514	0.025
ENSG00000212456	RNVU1-13	0	2.89777	7.34514	0.043
ENSG00000003096	KLHL13	0.0172958	0.469017	4.76114	5.00E-04
ENSG00000215262	KCNU1	0.0602063	0.579293	3.26631	0.013
ENSG00000101144	BMP7	0.0755262	0.621689	3.04114	8.00E-04
ENSG00000189334	S100A14	0.104125	0.691765	2.73196	3.05E-03
ENSG00000152763	WDR78	0.173957	1.15255	2.72802	1.45E-03
ENSG00000197140	ADAM32	0.208588	1.37697	2.72277	2.45E-03
ENSG00000127084	FGD3	0.0891125	0.462018	2.37425	0.026
ENSG00000160460	SPTBN4	0.211645	1.0432	2.30129	1.50E-04
ENSG00000242550/// ENSG00000197632	SERPINB10	0.132319	0.587327	2.15015	7.50E-04
ENSG00000143341	HMCN1	0.187817	0.808715	2.1063	0.039
ENSG00000239473	RPL7P38	0.162947	0.692614	2.08765	0.044
ENSG00000140563	MCTP2	0.0919062	0.38658	2.07253	9.50E-04
ENSG00000147872	PLIN2	93.6029	387.497	2.04956	5.00E-05
ENSG00000004799	PDK4	2.47102	9.38758	1.92564	5.00E-05
ENSG00000060566	CREB3L3	0.199814	0.722407	1.85415	1.50E-03
ENSG00000087916	SLC6A14	0.119998	0.433308	1.85238	0.017
ENSG00000121101	TEX14	0.131666	0.474859	1.85062	0.029
ENSG00000184012	TMPRSS2	0.243279	0.872223	1.84208	4.90E-03
ENSG00000184305	CCSER1	0.133902	0.463072	1.79006	0.020
ENSG00000167772	ANGPTL4	12.8031	44.2304	1.78854	5.00E-05
ENSG00000139269	INHBE	0.140902	0.482176	1.77487	0.033
ENSG00000128965	CHAC1	14.0622	46.5167	1.72592	5.00E-05
ENSG00000104413	ESRP1	0.246026	0.783711	1.67151	0.022
ENSG00000051108	HERPUD1	157.727	496.484	1.65431	5.00E-05
ENSG00000144355	DLX1	0.123688	0.388723	1.65204	6.70E-03
ENSG00000175197	DDIT3	52.9803	160	1.59455	5.00E-05
ENSG00000183317	EPHA10	0.133315	0.40035	1.58642	0.044
ENSG00000128165	ADM2	1.72528	5.07581	1.55681	5.00E-05
ENSG00000223345	HIST2H2BA	0.98125	2.81864	1.52231	0.032
ENSG00000135903	PAX3	0.158953	0.451991	1.5077	0.032
ENSG00000181722	ZBTB20	0.425491	1.20977	1.50754	0.015

ENSG00000130513	GDF15	20.9477	58.9224	1.49203	5.00E-05
ENSG00000125409	TEKT3	0.203787	0.568952	1.48125	0.014
ENSG00000130766	SESN2	6.02464	16.6168	1.4637	5.00E-05
ENSG00000168772	CXXC4	0.282382	0.761563	1.43132	7.25E-03
ENSG00000153930	ANKFN1	0.956443	2.57447	1.42853	2.00E-04
ENSG00000165935	SMCO2	0.993131	2.66633	1.4248	0.034
ENSG00000157551///<br>ENSG00000198054	KCNJ15	0.163938	0.439266	1.42194	5.95E-03
ENSG00000156804	FBXO32	19.0915	49.2924	1.36844	5.00E-05
ENSG00000135638	EMX1	0.147553	0.380274	1.36581	0.043
ENSG00000134864	GGACT	0.889396	2.29178	1.36557	0.036
ENSG00000154654	NCAM2	0.632685	1.62917	1.36458	0.020
ENSG00000088992	TESC	0.410002	1.05417	1.3624	0.032
ENSG00000067715	SYT1	0.713704	1.82926	1.35786	0.024
ENSG00000227533	SLC2A1-AS1	0.318854	0.809367	1.3439	0.014
ENSG00000116761	CTH	17.7104	44.6686	1.33466	5.00E-05
ENSG00000162892	IL24	8.51645	21.4041	1.32956	0.011
ENSG00000174808	BTC	0.279233	0.699148	1.32413	7.60E-03
ENSG00000165868	HSPA12A	0.179321	0.44437	1.30922	0.032
ENSG00000182931	WFDC10B	0.695606	1.70225	1.2911	0.026
ENSG00000128590	DNAJB9	21.1266	51.6861	1.29072	5.00E-05
ENSG00000150961	SEC24D	29.8738	72.7621	1.2843	5.00E-05
ENSG00000147082	CCNB3	0.371613	0.89877	1.27415	0.030
ENSG00000214575///<br>ENSG00000182774///<br>ENSG00000260836	CPEB1	0.242708	0.581981	1.26175	8.00E-04
ENSG00000172216	CEBPB	22.7336	54.4971	1.26135	5.00E-05
ENSG00000086696	HSD17B2	0.344045	0.824476	1.26088	0.032
ENSG00000088826	SMOX	21.4847	50.8117	1.24185	0.038
ENSG00000139211	AMIGO2	25.9909	61.447	1.24133	5.00E-05
ENSG00000116285	ERRFI1	234.407	553.847	1.24047	5.00E-05
ENSG00000164400	CSF2	8.55727	20.1346	1.23445	5.00E-05
ENSG00000089163	SIRT4	0.748072	1.75825	1.23289	0.014
ENSG00000120519	SLC10A7	3.35725	7.88022	1.23096	4.25E-03
ENSG00000196542	SPTSSB	0.869262	2.02771	1.22199	0.011
ENSG00000101489	CELF4	0.368282	0.85614	1.21704	8.90E-03
ENSG00000166750	SLFN5	1.50574	3.49537	1.21497	5.00E-05
ENSG00000197279	ZNF165	1.3469	3.12192	1.21279	5.00E-05
ENSG00000151012	SLC7A11	7.34881	16.8939	1.20092	5.00E-05
ENSG00000159905	ZNF221	0.563026	1.28933	1.19535	0.022
ENSG00000184545	DUSP8	1.8801	4.26325	1.18115	4.20E-03
ENSG00000197697	HIST1H2BE	1.77782	4.01761	1.17623	5.05E-03

ENSG00000174912	METTL15P1	0.475836	1.07309	1.17323	0.012
ENSG00000196628	TCF4	0.350709	0.786028	1.16431	5.45E-03
ENSG00000101255	TRIB3	41.9728	93.5369	1.15608	5.00E-05
ENSG00000115008	IL1A	0.331623	0.737589	1.15327	3.45E-03
ENSG00000132881	RSG1	1.48833	3.29376	1.14604	0.029
ENSG00000105327	BBC3	5.37137	11.7324	1.12714	5.00E-05
ENSG00000180758	GPR157	2.95892	6.45638	1.12566	0.024
ENSG00000187193	MT1X	119.86	259.903	1.11663	5.00E-05
ENSG00000153714	LURAP1L	7.13343	15.4657	1.1164	5.00E-05
ENSG00000138670	RASGEF1B	0.322582	0.683632	1.08355	0.028
ENSG00000081041	CXCL2	5.30262	11.2025	1.07904	5.00E-05
ENSG00000182866	LCK	0.217938	0.457061	1.06847	0.030
ENSG00000172071	EIF2AK3	11.253	23.4517	1.05938	8.50E-04
ENSG00000163827	LRRC2	0.556179	1.14523	1.04201	6.00E-04
ENSG00000119138	KLF9	1.29261	2.64659	1.03385	5.00E-05
ENSG00000150051	MKX	1.52562	3.12353	1.03379	1.00E-04
ENSG00000101842	VSIG1	0.729835	1.48716	1.02691	0.038
ENSG00000146592	CREB5	2.27821	4.63666	1.02518	2.50E-03
ENSG00000125285	SOX21	0.25439	0.514564	1.01631	9.45E-03
ENSG00000168672	FAM84B	14.095	28.4574	1.01362	4.00E-04
ENSG00000261824	LINC00662	14.1092	28.4403	1.01129	2.00E-04
ENSG00000187672	ERC2	1.03938	2.07395	0.996655	0.023
ENSG00000185650	ZFP36L1	63.5182	126.662	0.995738	5.00E-05
ENSG00000164932	CTHRC1	1.65444	3.29771	0.995116	0.010
ENSG00000220785	MTMR9LP	0.534069	1.06241	0.992241	4.05E-03
ENSG00000164463	CREBRF	2.92192	5.80538	0.990474	5.00E-05
ENSG00000137193	PIM1	26.0825	51.6991	0.987059	5.00E-05
ENSG00000138166	DUSP5	79.6557	156.805	0.97712	5.00E-05
ENSG00000011465	DCN	0.379118	0.745555	0.975668	0.049
ENSG00000187801	ZFP69B	3.23187	6.32588	0.968897	5.00E-05
ENSG00000140044	JDP2	15.5376	30.288	0.962987	5.00E-05
ENSG00000136235	GPNMB	0.646573	1.25139	0.952652	0.042
ENSG00000168003	SLC3A2	381.435	737.146	0.950512	5.00E-05
ENSG00000118971	CCND2	0.528409	1.01816	0.946236	0.013
ENSG00000198417	MT1F	5.73049	10.999	0.940645	2.30E-03
ENSG00000021300	PLEKHB1	2.84649	5.45101	0.937337	8.05E-03
ENSG00000126368	NR1D1	4.9725	9.50748	0.935092	0.025
ENSG00000008086	CDKL5	0.321679	0.608574	0.919812	0.030
ENSG00000170689	HOXB9	11.3578	21.4012	0.914012	5.00E-05
ENSG00000233117	LINC00702	6.72302	12.6555	0.912582	1.75E-03
ENSG00000087074	PPP1R15A	85.9027	161.053	0.906757	5.00E-05

ENSG00000136244	IL6	14.9122	27.3797	0.876612	5.00E-05
ENSG00000115290	GRB14	1.15348	2.10897	0.870546	0.046
ENSG00000145860	RNF145	59.9747	109.444	0.867762	1.00E-04
ENSG00000136997	MYC	91.5875	166.494	0.862251	5.00E-05
ENSG00000163739	CXCL1	3.63168	6.54954	0.850757	4.00E-04
ENSG00000127311	HELB	0.208265	0.374033	0.844747	0.048
ENSG00000158022	TRIM63	0.51366	0.91945	0.839957	0.013
ENSG00000110852	CLEC2B	3.29411	5.88431	0.836983	1.80E-03
ENSG00000143507	DUSP10	10.115	18.0522	0.835681	5.00E-05
ENSG00000166073///<br>ENSG00000150667	GPR176	8.78535	15.6489	0.832887	6.00E-04
ENSG00000147852	VLDLR	9.00647	15.9849	0.827676	5.00E-05
ENSG00000167550	RHEBL1	3.3955	6.0212	0.82643	6.90E-03
ENSG00000100219	XBP1	62.1633	110.115	0.824873	5.00E-05
ENSG00000111912	NCOA7	9.68114	17.1121	0.821771	1.15E-03
ENSG00000106105	GARS	263.011	462.484	0.814279	7.20E-03
ENSG00000128645	HOXD1	2.72701	4.79338	0.813724	0.028
ENSG00000108370	RGS9	2.40355	4.21655	0.810899	0.010
ENSG00000185652	NTF3	1.00624	1.76518	0.810845	0.019
ENSG00000197841	ZNF181	3.1852	5.58139	0.80924	5.95E-03
ENSG00000196517	SLC6A9	7.59204	13.26	0.804517	2.00E-04
ENSG00000139209	SLC38A4	4.58576	8.00719	0.804136	0.022
ENSG00000165891	E2F7	29.1705	50.9145	0.803566	5.00E-05
ENSG00000136826	KLF4	16.9272	29.525	0.802594	5.00E-05
ENSG00000147050	KDM6A	6.15219	10.7167	0.800684	3.55E-03
ENSG00000138061	CYP1B1	17.853	31.0924	0.800397	5.00E-05
ENSG00000177426	TGIF1	77.0166	134.093	0.79999	5.00E-05
ENSG00000172915	NBEA	0.723201	1.25431	0.794424	0.050
ENSG00000156273///<br>ENSG00000183653	BACH1	15.9347	27.543	0.789519	0.017
ENSG00000134294	SLC38A2	150.19	259.509	0.788999	1.75E-03
ENSG00000099337	KCNK6	3.7851	6.53723	0.788346	5.00E-05
ENSG00000172260	NEGR1	9.0541	15.6233	0.787053	0.030
ENSG00000212734	C17orf100	0.67492	1.1622	0.784068	0.014
ENSG00000113615	SEC24A	7.14223	12.2788	0.78172	1.85E-03
ENSG00000048052	HDAC9	10.8427	18.589	0.777717	1.00E-04
ENSG00000116717	GADD45A	37.4044	64.0088	0.775064	5.00E-05
ENSG00000165029	ABCA1	1.14349	1.95675	0.775017	1.50E-04
ENSG00000164221	CCDC112	7.92248	13.5426	0.773484	1.20E-03
ENSG00000166289	PLEKHF1	3.89892	6.65273	0.770872	1.00E-04
ENSG00000165030	NFIL3	17.0886	29.1089	0.768422	5.00E-05
ENSG00000174749	C4orf32	6.98828	11.8514	0.762047	5.00E-05

ENSG00000086619	ERO1LB	3.17232	5.37641	0.761102	1.30E-03
ENSG00000175155	YPEL2	1.21094	2.05091	0.760138	5.00E-04
ENSG00000164244	PRRC1	30.0203	50.81	0.759172	2.50E-04
ENSG00000169359	SLC33A1	10.692	18.0863	0.758362	4.40E-03
ENSG00000120129	DUSP1	38.3475	64.7803	0.756424	5.00E-05
ENSG00000118507	AKAP7	2.25997	3.81105	0.753888	0.020
ENSG00000059804	SLC2A3	10.4377	17.5341	0.748367	2.30E-03
ENSG00000116584	ARHGEF2	77.2629	129.762	0.74802	0.027
ENSG00000140743	CDR2	17.8456	29.9625	0.747592	1.00E-03
ENSG00000105829	BET1	20.8893	35.054	0.746813	1.00E-04
ENSG00000170385	SLC30A1	13.0623	21.8602	0.742894	5.00E-05
ENSG00000221878	PSG7	0.525546	0.879391	0.742688	0.043
ENSG00000139946	PELI2	0.817878	1.36785	0.741947	2.50E-03
ENSG00000142178	SIK1	6.19051	10.3092	0.735797	5.00E-05
ENSG00000114200	BCHE	1.48039	2.45787	0.731432	0.041
ENSG00000044574	HSPA5	509.641	845.973	0.731129	5.00E-05
ENSG00000176046	NUPR1	15.8745	26.3369	0.730368	6.50E-04
ENSG00000145050	MANF	334.025	551.999	0.724711	1.50E-04
ENSG00000113448	PDE4D	2.9324	4.84313	0.72386	3.50E-04
ENSG00000112812	PRSS16	1.50732	2.48893	0.723544	0.014
ENSG00000073849	ST6GAL1	1.90616	3.14708	0.723345	0.020
ENSG00000257773	ST13P3	1.48989	2.45845	0.72254	0.013
ENSG00000153879	CEBPG	19.5367	32.2358	0.722475	5.00E-05
ENSG00000151135	TMEM263	50.2101	82.7928	0.721528	5.00E-05
ENSG00000114796	KLHL24	6.00037	9.88764	0.720575	0.017
ENSG00000164674	SYTL3	0.711779	1.1722	0.719719	0.046
ENSG00000100628	ASB2	0.478828	0.78411	0.711549	0.049
ENSG00000173281	PPP1R3B	2.30877	3.77925	0.710975	5.00E-05
ENSG00000163347	CLDN1	3.89968	6.35953	0.705565	4.00E-04
ENSG00000105856	HBP1	24.2348	39.5058	0.704983	0.020
ENSG00000133639///<br>ENSG00000257242	BTG1	64.5822	105.133	0.703004	3.20E-03
ENSG00000166002	SMCO4	12.4097	20.2	0.702888	0.014
ENSG00000105281	SLC1A5	152.497	247.844	0.700654	0.040
ENSG00000165985	C1QL3	0.368446	0.597924	0.69851	0.037
ENSG00000166123	GPT2	15.0387	24.2711	0.690563	0.010
ENSG00000205189	ZBTB10	2.97316	4.79294	0.688914	5.55E-03
ENSG00000128692	EIF2S2P4	1.85066	2.97596	0.685315	7.20E-03
ENSG00000100558	PLEK2	28.634	45.9858	0.683456	2.70E-03
ENSG00000143067	ZNF697	3.75011	6.02029	0.682899	5.00E-05
ENSG00000141627	DYM	10.3869	16.6541	0.681113	2.60E-03
ENSG00000169306	IL1RAPL1	2.42869	3.89282	0.680639	1.60E-03

ENSG00000150907	FOXO1	2.40334	3.83404	0.673823	0.046
ENSG00000204767	FAM196B	2.91806	4.6417	0.669643	0.021
ENSG00000137331	IER3	417.898	664.571	0.669274	5.00E-05
ENSG00000164292	RHOBTB3	33.772	53.4618	0.662682	0.011
ENSG00000163393	SLC22A15	1.77932	2.81504	0.66183	0.010
ENSG00000114423	CBLB	4.51061	7.12975	0.660528	2.25E-03
ENSG00000146278	PNRC1	33.9363	53.5411	0.657819	5.00E-04
ENSG00000066455	GOLGA5	26.2167	41.1629	0.65086	4.15E-03
ENSG00000169249	ZRSR2	5.0872	7.97921	0.649374	0.038
ENSG00000143878	RHOB	8.61609	13.5018	0.648049	3.50E-04
ENSG00000198855	FICD	8.3977	13.1385	0.645731	0.036
ENSG00000223891	OSER1-AS1	8.45954	13.2223	0.644326	2.90E-03
ENSG00000143479	DYRK3	8.01316	12.4908	0.640419	3.50E-04
ENSG00000153066	TXNDC11	18.4096	28.6397	0.637557	0.031
ENSG00000128228	SDF2L1	26.3253	40.8955	0.635495	1.50E-04
ENSG00000125845	BMP2	0.488335	0.758329	0.634952	0.022
ENSG00000178150	ZNF114	9.6482	14.9264	0.629536	0.017
ENSG00000135338	LCA5	0.881954	1.3643	0.629386	4.05E-03
ENSG00000133135	RNF128	1.55702	2.39222	0.619565	4.20E-03
ENSG00000113083	LOX	29.665	45.5769	0.619541	4.60E-03
ENSG00000164171	ITGA2	23.3627	35.8542	0.617931	1.35E-03
ENSG00000182827	ACBD3	22.3597	34.2384	0.614711	0.032
ENSG00000075420	FNDC3B	17.046	26.0999	0.614616	9.05E-03
ENSG00000115902	SLC1A4	8.28859	12.6898	0.614467	2.25E-03
ENSG00000139289	PHLDA1	114.23	174.734	0.613224	4.00E-04
ENSG00000163734	CXCL3	2.64091	4.03045	0.609904	0.019
ENSG00000134070	IRAK2	5.606	8.55493	0.609785	4.00E-04
ENSG00000167074	TEF	1.16823	1.78117	0.608507	6.70E-03
ENSG00000118503	TNFAIP3	2.87887	4.37591	0.604082	0.033
ENSG00000204428	LY6G5C	2.47972	3.76891	0.603968	0.049
ENSG00000119899	SLC17A5	20.4568	31.0847	0.603628	3.50E-04
ENSG00000267374	LINC00669	1.12648	1.71052	0.602618	0.016
ENSG00000169087	HSPBAP1	4.11056	6.23402	0.600827	0.015
ENSG00000149150	SLC43A1	7.31935	11.0992	0.600671	5.10E-03
ENSG00000065060	UHRF1BP1	5.13249	7.77946	0.600011	0.012
ENSG00000163884	KLF15	1.2924	1.95779	0.599175	0.012
ENSG00000168209	DDIT4	57.7349	87.4505	0.599023	0.022
ENSG00000179222	MAGED1	48.2783	73.0275	0.597065	1.85E-03
ENSG00000156966	B3GNT7	3.57481	5.40345	0.596012	1.20E-03
ENSG00000138764	CCNG2	24.7554	37.3278	0.592511	5.40E-03
ENSG00000078177	N4BP2	1.68613	2.54123	0.591811	1.75E-03

ENSG00000074935	TUBE1	9.2443	13.9292	0.591481	0.029
ENSG00000182841///<br>ENSG00000251913	RRP7B	6.79844	10.2281	0.589256	0.036
ENSG00000111371	SLC38A1	40.1876	60.4582	0.589188	6.00E-04
ENSG00000157693	C9orf91	4.40974	6.63259	0.58888	0.042
ENSG00000122674	CCZ1	3.0699	4.61494	0.588121	0.032
ENSG00000111252	SH2B3	32.969	49.535	0.587339	2.00E-04
ENSG00000175592	FOSL1	180.777	271.298	0.585666	1.50E-04
ENSG00000176018	LYSMD3	13.178	19.7089	0.580714	9.50E-04
ENSG00000006459	KDM7A	0.762445	1.13857	0.578519	8.70E-03
ENSG00000150593	PDCD4	111.66	166.661	0.577814	3.95E-03
ENSG00000172006	ZNF554	0.785827	1.17073	0.575131	0.037
ENSG00000234975	FTH1P2	9.11561	13.579	0.574971	0.026
ENSG00000116711	PLA2G4A	1.82429	2.71066	0.571311	0.022
ENSG00000126947	ARMCX1	30.1146	44.7122	0.570205	4.50E-04
ENSG00000091844	RGS17	1.33374	1.97463	0.566104	0.028
ENSG00000181788	SIAH2	16.2343	24.022	0.565314	9.50E-04
ENSG00000265688	MAFG-AS1	3.51501	5.19917	0.564751	0.020
ENSG00000178764	ZHX2	2.27914	3.37085	0.564623	2.40E-03
ENSG00000172086	KRCC1	14.1073	20.8568	0.564076	1.05E-03
ENSG00000139793	MBNL2	17.1682	25.3113	0.560038	0.042
ENSG00000144674	GOLGA4	21.0748	30.9979	0.556649	1.35E-03
ENSG00000024862	CCDC28A	10.7862	15.8511	0.555397	2.20E-03
ENSG00000166483	WEE1	29.3766	43.1349	0.554192	0.031
ENSG00000198929///<br>ENSG00000239887///<br>ENSG00000254706	NOS1AP	2.86946	4.20961	0.552904	8.60E-03
ENSG00000160181	TFF2	16.297	23.8567	0.549789	6.00E-03
ENSG00000148396	SEC16A	13.9626	20.3675	0.544701	0.038
ENSG00000158470	B4GALT5	22.3827	32.6296	0.543797	4.50E-04
ENSG00000011638	TMEM159	18.6212	27.0844	0.540516	0.010
ENSG00000258818///<br>ENSG00000214274	RNASE4	22.0649	32.0713	0.539529	5.40E-03
ENSG00000126860///<br>ENSG00000185862///<br>ENSG00000265118	EVI2A	54.1924	78.5765	0.536007	2.50E-03
ENSG00000180730	SHISA2	6.1535	8.90825	0.533735	3.15E-03
ENSG00000172059	KLF11	8.66323	12.5398	0.533537	5.55E-03
ENSG00000143457	GOLPH3L	18.139	26.2289	0.532064	7.80E-03
ENSG00000198961	PJA2	32.7058	47.2469	0.530673	6.50E-04
ENSG00000198453	ZNF568	2.25737	3.25867	0.52964	0.040
ENSG00000120742	SERP1	47.2387	68.1753	0.52928	0.023
ENSG00000067082	KLF6	82.3739	118.773	0.527952	6.70E-03

ENSG00000147614	ATP6V0D2	2.74535	3.95567	0.526932	0.015
ENSG00000164615	CAMLG	46.3666	66.791	0.526569	4.85E-03
ENSG00000206538	VGLL3	1.31215	1.88861	0.525396	0.016
ENSG00000176624	MEX3C	14.5472	20.9153	0.523817	1.30E-03
ENSG00000171928	TVP23B	19.4041	27.8905	0.523412	2.10E-03
ENSG00000014123	UFL1	5.52047	7.93162	0.522824	4.35E-03
ENSG00000102804	TSC22D1	198.955	285.733	0.522226	9.00E-04
ENSG00000197355	UAP1L1	5.68161	8.13294	0.517478	3.15E-03
ENSG00000138678	AGPAT9	36.0411	51.5225	0.515558	2.80E-03
ENSG00000196151	WDSUB1	5.56166	7.94292	0.514154	8.30E-03
ENSG00000163565	IFI16	74.1405	105.877	0.514058	6.75E-03
ENSG00000128272	ATF4	261.059	372.748	0.513826	1.05E-03
ENSG00000172432	GTPBP2	47.2592	67.1899	0.507649	0.039
ENSG00000074695	LMAN1	50.3576	71.5148	0.506033	0.011
ENSG00000243137	PSG4	5.37581	7.63212	0.5056	0.036
ENSG00000140948	ZCCHC14	5.03311	7.14375	0.505232	3.10E-03
ENSG00000165312	OTUD1	11.0076	15.6175	0.504664	3.55E-03
ENSG00000164949	GEM	30.9927	43.9645	0.504414	0.024
ENSG00000169432	SCN9A	1.56498	2.21862	0.503519	0.025
ENSG00000135185	TMEM243	28.9884	41.0943	0.503464	0.035
ENSG00000155304	HSPA13	41.4895	58.7423	0.501655	0.027
ENSG00000136158	SPRY2	64.5467	91.3407	0.500915	8.00E-04
ENSG00000170448	NFXL1	8.72607	12.3443	0.500445	0.040
ENSG00000121797	CCRL2	24.9792	35.3008	0.498973	5.05E-03
ENSG00000150526///<br>ENSG00000150527///<br>ENSG00000258941	MIA2	16.9723	23.9753	0.498368	4.30E-03
ENSG00000102401	ARMCX3	25.9315	36.6261	0.498166	0.028
ENSG00000261455	LINC01003	1.68369	2.37778	0.497991	0.039
ENSG00000118689	FOXO3	5.98516	8.43494	0.494989	2.25E-03
ENSG00000109452	INPP4B	11.438	16.1114	0.494246	8.50E-03
ENSG00000177283	FZD8	5.31936	7.49179	0.494059	4.80E-03
ENSG00000172007	RAB33B	3.3855	4.75541	0.4902	0.019
ENSG00000150991	UBC	897.135	1259.69	0.489672	0.016
ENSG00000124786	SLC35B3	16.443	23.0729	0.488726	5.45E-03
ENSG00000174501	ANKRD36C	1.02849	1.44184	0.487386	0.040
ENSG00000125148	MT2A	1798.53	2518.04	0.485487	4.85E-03
ENSG00000164647	STEAP1	8.22823	11.5196	0.485431	0.026
ENSG00000141384	TAF4B	3.76744	5.26468	0.482761	8.95E-03
ENSG00000178726	THBD	21.9068	30.5427	0.47945	1.85E-03
ENSG00000163659	TIPARP	23.5018	32.743	0.478416	4.15E-03
ENSG00000100485	SOS2	9.7856	13.6164	0.476617	4.75E-03

ENSG00000168944	CEP120	7.40703	10.3004	0.475736	5.65E-03
ENSG00000198856	OSTC	201.803	280.034	0.472656	1.95E-03
ENSG00000169554	ZEB2	3.42674	4.74854	0.470645	0.046
ENSG00000069712	KIAA1107	0.767628	1.06343	0.470253	0.038
ENSG00000187840	EIF4EBP1	125.48	173.822	0.470159	3.10E-03
ENSG00000177951	BET1L	24.1993	33.5082	0.469544	0.040
ENSG00000164284	GRPEL2	7.68178	10.6367	0.469536	5.50E-03
ENSG00000137601	NEK1	3.74551	5.17074	0.465211	0.013
ENSG00000165181	C9orf84	1.06111	1.46352	0.463869	0.025
ENSG00000128052	KDR	1.30625	1.799	0.461763	0.047
ENSG00000112715	VEGFA	80.5748	110.872	0.460493	0.039
ENSG00000131725	WDR44	13.2292	18.1973	0.459992	0.014
ENSG00000168495	POLR3D	15.4683	21.2757	0.459892	0.018
ENSG00000068912	ERLEC1	48.4281	66.5793	0.459228	0.010
ENSG00000152749	GPR18	2.87531	3.95265	0.459105	5.85E-03
ENSG00000135842	FAM129A	11.3246	15.5546	0.45788	3.00E-03
ENSG00000169715	MT1E	758.455	1039.88	0.455285	0.011
ENSG00000114529	C3orf52	4.10565	5.61735	0.452279	0.027
ENSG00000136869	TLR4	8.15508	11.1548	0.451893	5.80E-03
ENSG00000185842	DNAH14	24.5437	33.5521	0.45105	0.032
ENSG00000116604	MEF2D	10.9373	14.9487	0.450761	9.30E-03
ENSG00000181274	FRAT2	7.49694	10.2416	0.450067	0.011
ENSG00000114861	FOXP1	11.7828	16.0881	0.449321	0.031
ENSG00000104635	SLC39A14	33.9893	46.3935	0.448845	4.45E-03
ENSG00000144824///<br>ENSG00000240891	PHLDB2	9.17602	12.5232	0.448668	0.043
ENSG00000152503	TRIM36	4.06103	5.53344	0.446331	0.049
ENSG00000148572	NRBF2	20.9969	28.5879	0.445228	6.15E-03
ENSG00000169155	ZBTB43	4.98647	6.78424	0.444169	7.60E-03
ENSG00000110048	OSBP	21.0231	28.6018	0.444132	0.042
ENSG00000148288///<br>ENSG00000160271	GBGT1	20.8857	28.413	0.444035	0.038
ENSG00000182831	C16orf72	8.94939	12.1564	0.441855	0.016
ENSG00000124882	EREG	5.99395	8.14056	0.441621	8.40E-03
ENSG00000170425	ADORA2B	62.991	85.5475	0.441581	4.35E-03
ENSG00000187164	KIAA1598	7.11055	9.64621	0.440001	0.025
ENSG00000049323	LTBP1	8.46529	11.4713	0.438404	0.047
ENSG00000165272	AQP3	6.76103	9.15488	0.437299	0.021
ENSG00000185753	CXorf38	8.15217	11.0218	0.435111	0.039
ENSG00000104047	DTWD1	17.276	23.3466	0.43444	0.034
ENSG00000147251	DOCK11	1.83541	2.47881	0.433546	0.020
ENSG00000165997	ARL5B	7.61952	10.2887	0.43329	7.95E-03

ENSG00000008869	HEATR5B	3.40604	4.59851	0.433071	0.042
ENSG00000173334	TRIB1	8.14327	10.987	0.432117	0.012
ENSG00000167797	CDK2AP2	67.5418	91.0237	0.430462	0.010
ENSG00000134955	SLC37A2	42.0356	56.6215	0.429736	0.016
ENSG00000115806	GORASP2	58.2626	78.4701	0.429571	0.041
ENSG00000134109	EDEM1	18.4315	24.8207	0.429371	0.014
ENSG00000110090	CPT1A	14.7485	19.8518	0.428697	0.014
ENSG00000145919	BOD1	102.911	138.33	0.426723	8.30E-03
ENSG00000083799	CYLD	18.835	25.2772	0.424425	0.037
ENSG00000115339	GALNT3	27.2376	36.4886	0.421846	0.040
ENSG00000131711	MAP1B	17.6632	23.6521	0.421228	5.90E-03
ENSG00000148680	HTR7	4.00914	5.35338	0.417159	0.021
ENSG00000182158	CREB3L2	9.55644	12.7078	0.411173	0.014
ENSG00000204019	CT83	18.3539	24.3089	0.405399	0.042
ENSG00000188042	ARL4C	36.1118	47.7979	0.404478	7.95E-03
ENSG00000112406	HECA	4.94064	6.51133	0.398254	0.018
ENSG00000145780	FEM1C	8.15635	10.7466	0.397891	0.013
ENSG00000197976	AKAP17A	7.68722	10.1202	0.396705	0.019
ENSG00000111801	BTN3A3	5.44471	7.16778	0.396672	0.040
ENSG00000102580	DNAJC3	16.2462	21.3785	0.396063	0.011
ENSG00000198833	UBE2J1	21.2484	27.9464	0.395306	0.012
ENSG00000168994	PXDC1	61.5391	80.8991	0.39462	0.019
ENSG00000181852	RNF41	16.6256	21.8541	0.394495	0.040
ENSG00000162236	STX5	44.4997	58.4683	0.39386	0.019
ENSG00000153989	NUS1	22.8171	29.973	0.393547	0.012
ENSG00000134259	NGF	10.9651	14.4032	0.393473	0.036
ENSG00000104312	RIPK2	19.0312	24.986	0.392755	0.020
ENSG00000137094	DNAJB5	6.23513	8.18549	0.39265	0.027
ENSG00000147905	ZCCHC7	11.7653	15.4303	0.39122	0.034
ENSG00000131732	ZCCHC9	37.4926	49.1016	0.389165	0.031
ENSG00000166016	ABTB2	4.82235	6.31344	0.388691	0.028
ENSG00000163235	TGFA	16.3733	21.4101	0.386948	0.019
ENSG00000125977	EIF2S2	134.203	175.079	0.383595	0.041
ENSG00000036054	TBC1D23	22.5979	29.4328	0.381232	0.031
ENSG00000165113	GKAP1	8.06563	10.5027	0.380906	0.042
ENSG00000110031	LPXN	12.0525	15.6891	0.380425	0.027
ENSG00000198369	SPRED2	17.1795	22.3566	0.380014	0.036
ENSG00000163162	RNF149	31.7843	41.316	0.378388	0.033
ENSG00000146242	TPBG	19.0224	24.7254	0.378292	0.019
ENSG00000111911	HINT3	9.47521	12.3131	0.377969	0.023
ENSG00000057704	TMCC3	8.32997	10.8218	0.377555	0.030

ENSG00000169826	CSGALNACT 2	31.6238	41.076	0.377286	0.013
ENSG00000157557	ETS2	10.744	13.9513	0.376868	0.038
ENSG00000162852	CNST	5.91767	7.66516	0.373288	0.034
ENSG00000151458	ANKRD50	5.29229	6.85382	0.373018	0.023
ENSG00000130449	ZSWIM6	6.73427	8.71268	0.371595	0.025
ENSG00000115520	COQ10B	18.7529	24.2615	0.371559	0.047
ENSG00000132823	OSER1	56.4468	72.9872	0.370751	0.016
ENSG00000197142	ACSL5	44.48	57.3579	0.366834	0.040
ENSG00000131871	VIMP	85.1464	109.796	0.366808	0.022
ENSG00000146386	ABRACL	54.9823	70.8043	0.364871	0.025
ENSG00000117597	DIEXF	6.03284	7.75932	0.363091	0.037
ENSG00000120063	GNA13	30.4638	39.1703	0.362668	0.020
ENSG00000173812	EIF1	900.645	1155.77	0.359828	0.029
ENSG00000159479	MED8	40.9284	52.4253	0.357159	0.032
ENSG00000136026	CKAP4	125.901	161.128	0.355912	0.024
ENSG00000128016	ZFP36	14.8213	18.9659	0.355733	0.035
ENSG00000240476	LINC00973	37.6366	48.1136	0.35431	0.033
ENSG00000168246	UBTD2	15.9582	20.3928	0.353761	0.026
ENSG00000140941	MAP1LC3B	51.0919	65.2748	0.353431	0.037
ENSG00000198455	ZXDB	3.22217	4.11643	0.35336	0.044
ENSG00000005238	FAM214B	11.004	14.0579	0.353354	0.036
ENSG00000123689	G0S2	172.33	220.127	0.353162	0.024
ENSG00000106541	AGR2	159.113	202.909	0.350788	0.028
ENSG00000157514	TSC22D3	13.5977	17.3402	0.350761	0.037
ENSG00000118482	PHF3	20.7791	26.4303	0.347059	0.029
ENSG00000151151	IPMK	2.7918	3.54955	0.346444	0.050
ENSG00000119986	AVPI1	14.0706	17.8825	0.345871	0.047
ENSG00000104412	EMC2	48.4732	61.5965	0.34566	0.049
ENSG00000189143	CLDN4	47.6689	60.5644	0.345422	0.029
ENSG00000153179	RASSF3	13.6162	17.2746	0.343329	0.038
ENSG00000135046	ANXA1	693.05	877.367	0.340222	0.028
ENSG00000115641	FHL2	64.6891	81.8412	0.339303	0.034
ENSG00000131263	RLIM	8.88609	11.2247	0.337057	0.033
ENSG00000169429	IL8	24.7324	31.0281	0.327169	0.045
ENSG00000187735	TCEA1	44.2754	55.4188	0.323869	0.039
ENSG00000159200	RCAN1	41.1904	51.4997	0.322253	0.039
ENSG00000117614	SYF2	54.7052	68.3857	0.322016	0.049
ENSG00000143614	GATAD2B	4.42957	5.53243	0.320746	0.049
ENSG00000115310	RTN4	436.913	544.978	0.318851	0.049
ENSG00000106299	WASL	13.0721	16.2838	0.316946	0.047

ENSG00000167306///<br>ENSG00000167315///<br>ENSG00000266997	MYO5B	32.6987	40.6949	0.315616	0.050
ENSG00000148677	ANKRD1	420.407	521.544	0.311	0.049
ENSG00000185222	WBP5	148.783	184.529	0.310636	0.042
ENSG00000130340	SNX9	18.2381	22.5853	0.30843	0.048

Supplementary Table 6: Top 18 genes uniquely inhibited by anacardic acid in MDA-MB-231 cells after 6 h treatment with 35 μM. Values are FKPM. All values are significantly different, P < 0.0005. The GO terms are listed in the order provided by DEG analysis and subsequent network analysis in MetaCore Gene Ontology (GO) algorithm to characterize the biological pathways altered by AnAc.

Gene	Control	AnAc	P value	Description	Top 3 GO terms	Function
LDLR	173.56	68.71	5.00E-05	low density lipoprotein receptor	GO:0070508: cholesterol import<br>GO:0030669: clathrin-coated endocytic vesicle membrane<br>GO:0030229:very-low-density lipoprotein particle receptor	*LDLR* is responsible for receptor-mediated endocytosis of cholesterol which is known to enhance tumorigenesis [1]. Overexpression of *LDLR* increased nuclear beta-catenin activity and facilitates MDA-MB-231 cell proliferation whereas siRNA targeting *LDLR* inhibited MDA-MB-231 cell proliferation [2]. Thus, our results are in agreement that downregulation of *LDLR* inhibits MDA-MB-231 cell viability.
MSMO1	161.87	68.71	5.00E-05	methylsterol monooxygenase 1	GO:0000254:C-4 methylsterol oxidase activity<br>GO:0008202: steroid metabolic process<br>GO:0006695: cholesterol biosynthetic process	MSMO protein is localized to the ER membrane and is believed to function in cholesterol biosynthesis. Its inhibition seems to functionally correlate with AnAc inhibition of *SCD* and *LDLR*. Aromatase inhibitors (AIs) stimulate *MSMO1* transcription in primary breast tumors [3]. The cholesterol biosynthetic pathway was upregulated in ERα+ long term estrogen deprived MCF-7 (MCF-7-LTED) cell lines and siMSMO1 inhibited MCF-7-LTED

						cell proliferation [3]. The authors hypothesized that 25-hydroxycholesterol and 27-hydroxycholesterol may replace E_2 to activate ERα in MCF-7-LTED cells. How inhibition of cholesterol synthesis may impact TNBC has not been examined, although lovostatin inhibited the proliferation and stimulated apoptosis of MDA-MB-231 cells *in vitro* [4].
BMP4	145.80	63.66	5.00E-05	Bone Morphogenetic Protein 4	GO:2000137: negative regulation of cell proliferation involved in heart morphogenesis GO:2000105: positive regulation of DNA-dependent DNA replication GO:2000007: negative regulation of metanephric comma-shaped body morphogenesis	BMP4 belongs to the transforming growth factor β (TGFβ) family of proteins that regulate cell proliferation, differentiation and motility [5]. E_2 decreased *BMP4* in MCF-7 cells [6]. BMP4 (100 ng/ml) treatment of MDA-MB-231 cells stimulated 3D migration in Matrigel and induced a 'stellate phenotype" by upregulating *MMP3* and *MMP14* [5]. Thus, the downregulation of *BMP4* by AnAc would be expected to inhibit the metastatic phenotype of MDA-MB-231 cells.
TMEM165	84.71	50.32	5.00E-05	Transmembrane Protein 165	GO:0016020: membrane GO:0035751: regulation of lysosomal lumen pH GO:0032472: Golgi calcium ion transport	*TMEM165* mutations cause a type-II Congenital Disorder of Glycosylation (CDG) affecting Golgi glycosylation processes [7]. TMEM165 is a Golgi ion transporter and is involved in Golgi manganese and calcium homeostasis [8]. Thus, AnAc

						reduction of *TMEM165* expression would be expected to reduce Golgi ion transport and inhibit the correct glycosylation of secretory proteins.
MICB	46.46	29.04	5.00E-05	MHC Class I Polypeptide-Related Sequence B	GO:0019882: antigen processing and presentation GO:0006955: immune response GO:0016020: membrane	MICA/MICB are ligands for the stimulatory natural killer (NK) cell receptor NKG2D [9]. NK cells are one of the major infiltrating immune cells in breast cancer that prevent tumorigenesis and metastasis [10]. Reduced expression of *MICB* in breast cancer stem cells (BCSC), due to increased miR-20a, contribute to their resistance to NK cell lysis and increase metastasis [9]. Interestingly, MICB was one of the genes downregulated by methylation in DMBA-induced mammary tumors from rats exposed to ethinyl estradiol *in utero* that was associated with TAM-resistance [11]. The plant-derived anticancer agent sulforaphane (SFN) increased MICA/MICB in MDA-MB-231 cells [12].
IL11	57.07	27.21	5.00E-05	Interleukin 11	GO:0005142: interleukin-11 receptor binding GO:0030219: megakaryocyte differentiation GO:0046888: negative regulation of hormone secretion	IL-11 is a mediator of osteoclastogenesis and predicts bone metastasis in breast cancer [13,14]. Inhibition of IL-11 (siRNA) inhibited MDA-MB-231 cell migration and invasion [15]. Thus, downregulation of *IL11* by AnAc would be beneficial to inhibit

						metastatic spread in breast cancer.
PCSK9	52.24	21.72	5.00E-05	Proprotein Convertase Subtilisin/Kexin Type 9	GO:0034190: apolipoprotein receptor binding<br>GO:0032802: low-density lipoprotein particle receptor catabolic process<br>GO:0001920: negative regulation of receptor recycling	PCSK9 is constitutively secreted as an inactive protease into the extracellular matrix and trans-Golgi network. PCSK9 is a Ca^{2+}-dependent protease plays a role in cholesterol and fatty acid metabolism by inducing degradation of the LDL receptor (LDLR), LDLR related protein 1 (LRP1), the apolipoprotein E receptor (ApoER), annexin A-2, and other targets [16]. Antibodies targeting PCSK9 (evolocumab and alirocumab) are FDA-approved for use in high cardiovascular risk patients to lower LDL-C and is used in patients who are statin-resistant or severe dyslipidemia [17]. Treatment of HepG2 and Huh7 HCC cells with LPS activated NFκB signaling, decreased *PCSK9* expression, and increased LDLR, HMGCR, and SREBF2 proteins, while increasing intracellular cholesterol and pro-inflammatory cytokines [18]. Given these reports and literature supporting that cholesterol has a variety of roles in carcinogenesis and that statins have anticancer activity (reviewed in [19,20]), we speculate the AnAc inhibition of *PCSK9*

						would be expected to decrease cholesterol uptake by MDA-MB-231 cells and this would adversely affect cell viability and migration.
AMOTL2	32.51	20.10	5.00E-05	Angiomotin Like 2	GO:0042802: identical protein binding GO:0055037: recycling endosome GO:0016324: apical plasma membrane	*AMOTL2* belongs to the Angiomotin (Amot) family of membrane-associated scaffold proteins and is involved in junction formation, apical polarity and actin cytoskeleton [21]. *AMOT2* expression correlates with loss of tissue architecture in tumors from human breast and colon cancer patients [22]. AMOTL2 plays a role tumor progression by inactivating the TRAPPII trans-Golgi complex which would disrupt cell polarity by inhibiting apical transport of polarity proteins [22]. Thus, the inhibition of *AMOTL2* by AnAc would be expected to keep breast cancer cells in a more 'epithelial' state.
TGOLN2	35.44	19.79	5.00E-05	Trans-Golgi Network Protein 2	GO:0030133: transport vesicle GO:0005802: trans-Golgi network GO:0005794: Golgi apparatus	TGOLN2 is a protein involved in trafficking to and from the trans-Golgi network. A search in the NURSA Transcriptomine database [23] revealed that E_2 (6 nM 24 h), genistein (300 nM), and BPA (10 µM) inhibited *TGOLN2* transcript expression in MCF-7 cells.

SDPR	23.69	14.27	5.00E-05	Serum Deprivation Response	GO:0001786: phosphatidylserine binding GO:0005901: caveolae GO:0005543: phospholipid binding	SDPR is a phosphatidylserine-interacting protein that was recently identified as a novel metastasis suppressor gene in breast cancer [24]. SDPR inhibits ERK and NFκB activation and *SDPR* expression is epigenetically silenced during metastatic cancer progression by DHA hypermethylation [24]. Another recent report demonstrated that overexpression of SDPR suppressed MDA-MB-231 cell proliferation and invasion and inhibited TGF-β signaling [25]. Thus, the downregulation of *SDPR* by AnAc would not appear to be beneficial in breast cancer.
TNFSF10	21.09	11.95	5.00E-05	Tumor Necrosis Factor Superfamily Member 10	GO:2001239: regulation of extrinsic apoptotic signaling pathway in absence of ligand GO:0090200: positive regulation of release of cytochrome c from mitochondria GO:0006919: activation of cysteine-type endopeptidase activity involved in apoptotic process	TNFSF10 is a cytokine member of the TNF ligand family that binds to a number of receptor, *e.g.*, TRAIL1, TRAIL2, TRAIL3, and TRAIL4, and induces apoptosis (reviewed in [26]). MDA-MB-231 cells are highly sensitive to TRAIL killing [27]. A SNP, rs13074711, 26.5 Kb upstream of TNFSF10 at 3q26.21, was significantly associated with risk of ERα-negative breast cancer in women of African ancestry [28].
SDC3	17.49	11.09	5.00E-05	Syndecan 3	GO:0008092: cytoskeletal protein binding GO:0016021: integral to membrane	SDC3 is a member of the transmembrane syndecan proteoglycan family that are cell surface receptors responsible for

					GO:0007603: phototransduction, visible light	interaction with extracellular matrix and intracellular kinases (reviewed in [29]). SDC3 interacts with tissue factor pathway inhibitor (TFPI) isoforms TFPIα at the surface of breast cancer cells [30]. However, *SDC3* expression is not different between normal and neoplastic human breast tissue samples [31]. Interesting, SDC3 interacts with c-Src in neurites [29].
FAM111B	15.65	8.01	5.00E-05	Family With Sequence Similarity 111 Member B	GO:0003824: catalytic activity	*FAM111B* encodes a protein with a trypsin-like cysteine/serine peptidase but little is known about its function other than that mutations cause hereditary fibrosing poikiloderma [32]. Interestingly, *FAM111B* and *BMP5* were among the top 10 genes downregulated in metformin (5 mM) + aspirin (2 mM) treated PANC-1 human pancreatic cancer cells [33]. Aspirin and anacardic acid (6-alkylsalicylic acid) are structurally related and both inhibit prostaglandin synthase [34].
CHST3	14.85	7.88	5.00E-05	Carbohydrate (Chondroitin 6) Sulfotransferase 3	GO:0050698: proteoglycan sulfotransferase activity GO:0008459: chondroitin 6-sulfotransferase activity GO:0006790: sulfur compound metabolic process	CHST3 catalyzes the transfer of sulfate to chondroitin and is a proteoglycan found in the extracellular matrix which is involved in cell migration and differentiation [35].
INHBB	15.13	7.85	5.00E-05	Inhibin Beta B	GO:0048178: negative regulation of hepatocyte	INHBB is a member of the TGFβ-family of proteins. INHBB is a

					growth factor biosynthetic process<br>GO:0046789: host cell surface receptor binding<br>GO:0044320: cellular response to leptin stimulus	heterodimer subunit of either activin or inhibin. INHBB heterodimerization with INHBC (Inhibin Beta C subunit) forms a glycoprotein hormone that down-regulates FSH production by anterior pituitary gonadotropes, acts as a paracrine factor regulating ovarian folliculogenesis and steroidogenesis, testis development and spermatogenesis, and has roles in the adrenal, bone metabolism, and retinal development and vision (reviewed in [36]). A SNP flanking *INHBB* was associated with breast size and breast cancer risk [37]. Inhibins inhibit activin-TGFβ interaction thus blocking activin's tumor suppressor activity; thus inhibin acts as a cancer cell survival factor [38]. Thus, AnAc inhibition of I*NHBB* would be expected to have a beneficial effect in breast cancer.
CDC25A	14.85	7.67	5.00E-05	Cell Division Cycle 25A	GO:0000087: mitotic M phase<br>GO:0009314: response to radiation<br>GO:0000082: G1/S transition of mitotic cell cycle	CDC25A is a phosphatase required for G1-S progression that cooperates with HER2 in promoting breast carcinogenesis and overexpression of CDC25A is involved in trastuzumab insensitivity [39]. CDC25A phosphatase inhibitors are of interest in cancer therapeutics [40].

						Thus, AnAc downregulation of *CDC25A* would appear to be beneficial in breast cancer.
LFNG	11.65	7.05	5.00E-05	LFNG O-Fucosylpeptide 3-Beta-N-Acetylglucosaminyltransferase	GO:0033829: O-fucosylpeptide 3-beta-N-acetylglucosaminyltransferase activity GO:0007386: compartment pattern specification GO:0030173: integral to Golgi membrane	LFNG modifies EGF repeats in the Notch extracellular domain thus suppressing Jagged/Notch signaling and thus has a tumor suppressor role in breast and other cancers [41-43]. However, *LFNG* appears to play different roles in luminal A *versus* TNBC since higher *LFNG* expression was correlated with good prognosis in luminal A whereas lower *LFNG* expression was associated with better disease free survival in TNBC [44].
BCAM	9.25	4.82	5.00E-05	Basal Cell Adhesion Molecule (Lutheran Blood Group)	GO:0005515: protein binding GO:0004888: transmembrane signaling receptor activity GO:0009897: external side of plasma membrane	BCAM is a cell surface receptor for laminin [45]. BCAM was identified as a cell surface marker in MDA-MB-231 cells [46]. Serum BCAM was significantly higher in breast cancer patients *versus* healthy controls [47]. A recent study reported that an antibody to BCAM inhibited A549 lung cancer cell migration [48].

Supplementary Table 7: Genes uniquely upregulated in MDA-MB-231 cells after 6 h. of 35 μM AnAc treatment. Genes are arranged from the most to least statistical significance. Values are gene expression are FKPM. All values are significantly different, P < 5.00E-05. The GO terms are listed in the order provided by DEG analysis and subsequent network analysis in MetaCore Gene Ontology (GO) algorithm to characterize the biological pathways altered by AnAc.

Gene	Control	AnAc	P value	Description	Top 3 GO terms	Function related to ERS responses
HSPA5<br><br>(also called BIP, GRP78)	509.64	845.97	5.00E-05	Heat shock 70kDa protein 5	GO:0060904: regulation of protein folding in endoplasmic reticulum GO:0034663: endoplasmic reticulum chaperone complex GO:0051787: misfolded protein binding	To relieve ERS, HSPA5/GRP78 binds unfolded proteins and prevents oligomerization of the ER transmembrane receptors EIF2AK3/PERK, IRE1α/ERN1, and ATF6 [49].
SLC3A2	381.44	737.15	5.00E-05	Solute Carrier Family 3	GO:0015827: tryptophan transport GO:0060356: leucine import GO:0015175: neutral amino acid transmembrane transporter activity	*SLC3A2* is a member of the solute carrier family and encodes a cell surface transmembrane protein which plays a role in intracellular Ca^{++} levels and transports L-type amino acids [50]. *SLC3A2* was upregulated by Tunicamycin-induced ER stress in neonatal rat cardiomyocytes [51]. SLC3A2 promotes cancer cell growth, cell survival, modulates integrin-induced signaling involved in cell migration and high expression in TNBC correlates with poor prognosis in breast tumors [52].
IER3	417.90	664.57	5.00E-05	Immediate Early Response 3	GO:0043066: negative regulation of apoptotic process; GO:2001020: regulation of response to DNA damage stimulus	

					GO:0006282: regulation of DNA repair	
ERRFI1 (also known as Gene 33, MIG6, and RALT)	234.41	553.85	5.00E-05	ERBB Receptor Feedback Inhibitor 1	GO:0045616: regulation of keratinocyte differentiation; GO:0031953: negative regulation of protein autophosphorylation GO:0060426: lung vasculature development	ERRFI1 is an adaptor protein in the cytoplasm, is induced by cell stress, and inhibits ERBB-PI3K-AKT signaling [53]. ERRFI1/MIG6 has tumor suppressor function in endometrial tumorigenesis [54] and in acute myeloid leukemia (AML) [55].
HERPUD1	157.73	496.48	5.00E-05	Homocysteine-inducible endoplasmic reticulum stress-inducible, ubiquitin-like domain member 1	GO:0006986: response to unfolded protein GO:0006987: activation of signaling protein activity involved in unfolded protein response GO:0031396: regulation of protein ubiquitination	HERPUD1 plays a role in UPR and the ER-associated protein degradation (ERAD) system by interacting with a subunit of the 19S regulatory complex of the 26S proteasome [56].
PLIN2	93.60	387.50	5.00E-05	perilipin 2	GO:0015909: long-chain fatty acid transport GO:0019915: lipid storage GO:0005811: lipid particle	PLIN2 is a key regulator of the UPR and ERS resolution [57]. *PLIN2* expression was increased in pancreatic β cells exposed to a lipid load or an ERS chemical inducer [57]. *PLIN2* is associated with fatty acid uptake, lipid accumulation, and intracellular lipid droplets in many tissues, including mammary gland [58]. Higher levels of *PLIN2* are thought to protect against lipotoxicity and insulin resistance in skeletal muscle [59].
MT1X (also called MT-IL or MT1)	119.86	259.90	5.00E-05	Metallothionein 1X	GO:0036018: cellular response to erythropoietin GO:0045926: negative regulation of growth	Metallothionein (MT) proteins bind heavy metals and are involved in detoxification, protection against oxidative damage, cell

					GO:0010038: response to metal ion	proliferation, and apoptosis [60]Overexpression of cardiac *MT1X* protects mice from intermittent hypoxia -induced ERS and cell death [61]. *MT1X* acts as a tumor suppressor in ovarian, liver, and colon cancer [60].
MYC	91.59	166.49	5.00E-05	V-Myc Avian Myelocytomatosis Viral Oncogene Homolog	GO:0006355: regulation of transcription, DNA-dependent GO:0003700: sequence-specific DNA binding transcription factor activity GO:0005634: nucleus	Previous studies demonstrate that overexpression of *MYC* in endocrine-resistant breast cancer cells allows cell survival in glucose-deprived conditions by increasing glutamine uptake and metabolism and upregulating the UPR including increases in *HSP5A, IRE1*, and *XBP1* [62]. Thus, the increase in *MYC* also appears to be part of the potential UPR response mediated by AnAc in MDA-MB-231 cells whereby the cells may be responding to altered cell metabolism by increasing glutamine metabolism.
PPP1R15A Also called GADD34	85.90	161.05	5.00E-05	Protein Phosphatase 1 Regulatory Subunit 15A	GO:0030968; endoplasmic reticulum unfolded protein response GO:0030512; negative regulation of transforming growth factor beta receptor signaling pathway GO:0007179; transforming growth factor beta receptor signaling pathway	PPP1R15A is a regulatory subunit of PP1 and functions in response to stressful growth arrest conditions and DNA damage by increasing total and phosphorylated (ser-15) p53, stimulating apoptosis and activating the ER stress response [63]

DDIT3<br><br>(also called C/EBP zeta, CHOP, CHOP10, GADD153	52.98	160.00	5.00E-05	DNA Damage Inducible Transcript 3	GO:2000016: negative regulation of determination of dorsal identity<br>GO:0043620: regulation of DNA-dependent transcription in response to stress<br>GO:0006986: response to unfolded protein	DDIT3/CHOP is a member of the CCAAT/enhancer binding protein (C/EBP) family of transcription factors [64]. DDIT3/CHOP plays an important role in ERS-induced apoptosis by increasing *PPP1R15A*/GADD34 and *BIM* and decreasing *BCL2* and also interacts with AP-1 to increase *PUMA* and *BAX* [65]. As in the previous paragraphs, the increase in *DDIT3* is another indication of ER stress induced by AnAc in MDA-MB-231 cells.
DUSP5	79.66	156.81	5.00E-05	Dual Specificity Phosphatase 5	GO:0017017: MAP kinase tyrosine/serine/threonine phosphatase activity<br>GO:0000188: inactivation of MAPK activity<br>GO:0016311: dephosphorylation	Upregulated in the ERS response of cultured zebrafish liver cells [66].
TGIF1	77.02	134.09	5.00E-05	TGFB Induced Factor Homeobox 1	GO:0038092: nodal signaling pathway<br>GO:0010470: regulation of gastrulation<br>GO:0048146: positive regulation of fibroblast proliferation	
ZFP36L1	63.52	126.66	5.00E-05	ZFP36 Ring Finger Protein-Like 1	GO:0001570: vasculogenesis<br>GO:0033077: T cell differentiation in thymus<br>GO:0043488: regulation of mRNA stability	

XBP1	62.16	110.12	5.00E-05	X-Box Binding Protein 1	GO:0060691: epithelial cell maturation involved in salivary gland development GO:0060096: serotonin secretion, neurotransmission GO:1900103: positive regulation of endoplasmic reticulum unfolded protein response	In response to the unfolded protein response of the endoplasmic reticulum (UPR^{ER}), XBP1 is upregulated by splicing of an unconventional 26-nt intron by the transmembrane kinase and ribonuclease IRE1 resulting in XBP1s which regulates transcription of targets for ERS response [67].
TRIB3 (also known as TRB3, SKIP3, and NIPK)	41.97	93.54	5.00E-05	Tribbles Pseudokinase 3	GO:0043405: regulation of MAP kinase activity GO:0004860: |protein kinase inhibitor activity GO:0048011: neurotrophin TRK receptor signaling pathway	*TRIB3* is an ERS-induced gene via the ATF4-CHOP pathway in normal and cancer cells [68]. TRIB3 protein interacts directly with CHOP and inhibits its activity, thus leading to apoptosis. TRB3 forms a complex with MIB1, JAG1 and the deubiquitinating enzyme USP9x to promote JAG1 transcriptional upregulation and Notch pathway activation in the tumor microenvironment [69].
SEC24D	29.87	72.76	5.00E-05	SEC24 homolog D	GO:0012507: ER to Golgi transport vesicle membrane GO:0019886: antigen processing and presentation of exogenous peptide antigen via MHC class II; GO:0048208: COPII vesicle coating	Increased in response to quinocetone to induce the ERS response in HepG2 cells and participates in ER vesicle trafficking [70].
DUSP1 (also called	38.35	64.78	5.00E-05	Dual Specificity Phosphatase 1	GO:0008330: protein tyrosine/threonine phosphatase activity	DUSP1 (MKP-1) is upregulated by ERS and inactivates all three MAPK families by

MKP-1, MKP1)					GO:0051447: negative regulation of meiotic cell cycle<br>GO:0004726: non-membrane spanning protein tyrosine phosphatase activity	dephosphorylating them at catalytic tyrosine and threonine residues, but it acts preferentially on p38 and JNK [71] leading to either cell survival or death depending on other cellular responses [72].
GADD45A	37.40	64.01	5.00E-05	Growth Arrest And DNA Damage Inducible Alpha	GO:1900745: positive regulation of p38MAPK cascade<br>GO:0000185: activation of MAPKKK activity<br>GO:0071479: cellular response to ionizing radiation	Increased in response to ERS in both ER+ and ER- breast cancer cells and leads to cell cycle arrest [73 74].
AMIGO2	25.99	61.45	5.00E-05	Adhesion Molecule With Ig Like Domain 2	GO:0043069: negative regulation of programmed cell death<br>GO:0007157: heterophilic cell-cell adhesion<br>GO:0007156: homophilic cell adhesion	AMIGO2 is a cell surface adhesion molecule that that has tumor suppressor activity in gastric cancer [75]. AMIGO2 is also a scaffold protein that anchors PDK1 in the cell membrane and suppressed angiogenesis in murine tumors [76]. Lower methylation and hence higher *AMIGO2* expression in lymph node metastases from TNBC patients correlated with better survival [77]. We also note that although AnAc has p300/PCAF histone acetyltransferase (HAT) inhibitor activity (reviewed in [78]), it was recently reported to increase DNA methylation and decrease histone acetylation of the *IL6* promoter in synovial fibroblasts from osteoarthritis patients [79].

						Whether AnAc stimulates the expression of *AMIGO2* by altered methylation is currently unknown and will require further examination.
GDF15 (also called MIC-1, NAG-1, PL74, PTGF-β, PDF, and PLAB).	20.95	58.92	5.00E-05	Growth Differentiation Factor 15	GO:0005125: cytokine activity<br>GO:0007179: transforming growth factor beta receptor signaling pathway<br>GO:0007267: cell-cell signaling	*GDF15*, a member of the TGF-β superfamily, is linked to the pathogenesis of cancer and is induced by ERS, and plays a role in apoptotic cell death [80]. *GDF15* is also induced by mitochondrial dysfunction [81]. GDF15 has tumor suppressor and apparent pro-survival activities (reviewed in [82]). *GDF15* is transcriptionally activated by a variety of natural products with anticancer activity, *e.g.*, flavonoids (apigenin, quercetin), isoflavonoids (genistein), catechin (epigallocatechin gallate), and stilbenoids (resveratrol), by activating transcription factors: EGR-1, p53, ATF-3, Sp1, and PPARγ [83].

Supplementary Table 8: Summary of sequence analysis. The raw data of our RNA-seq are available at Gene Expression Omnibus (GEO) database: accession number GSE78011.

Sample #, cells, treatment	Raw Reads	Raw Bases	Aligned Reads
1-MCF-7control_S12	36,573,300	2,751,962,330	27,245,316 (75.5%)
2-MCF-7control_S6	27,338,734	2,057,896,791	26,530,705 (97.0%)
3-MCF-7control_S2	25,740,013	1,937,678,403	24,577,882 (95.5%)
4-MCF-7AnActrt_S8	26,890,905	2,024,606,798	20,941,565 (77.9%)
5-MCF-7AnActrt_S1	27,109,962	2,042,102,260	26,196,287 (96.6%)
6-MCF-7AnActrt_S7	27,433,671	2,066,666,745	26,274,779 (95.8%)
7-MDA-MB-231control_S11	60,871,824	4,582,378,054	51,652,469 (84.9%)
8-MDA-MB-231control_S10	25,937,591	1,951,495,416	22,670,390 (87.4%)
10-MDA-MB-231AnActrt_S4	25,851,699	1,948,077,018	22,589,546 (87.4%)
11-MDA-MB-231AnActrt_S3	26,413,653	1,990,423,798	22,752,392 (86.1%)

Supplementary Table 9: Primers used for qPCR.

Gene	Forward primer	reverse primer	refere nce
SCD	5’-TTCCCGACGTGGCTTTTTCT-3’	5’-AGCCAGGTTTGTAGTACCTCC-3’	[84]
STIM1	5’-TGTGGAGCTGCCTCAGTATG-3’	5’-CTTCAGCACAGTCCCTGTCA-3	[85]
EGR1	5’-TGCCCAGTGGAGTCCTGTGAT-3’	5’-CGCTCCTGGCAAACTTTCTTC-3’	[85]
CDIP	5′ ATGACCTACAAATCTCAACATGACTTGGAT-3’	5′-AGAGGAAGGGACGCCCTAACAGAGTTAC-3’	[86]
NSIG1	5’-GACAGTCACCTCGGAGAACC-3’	5’-CACCAAAGGCCCAAAGATAG-3’	[87]
MIR22HG	5’-CGGACGCAGTGATTTGCT-3’	5’-GCTTTAGCTGGGTCAGGACA-3	[88]
CPT1A	5'-CCTCCAGTTGGCTTATCGTG-3	5'-TTCTTCGTCTGGCTGGACAT-3'	[89]

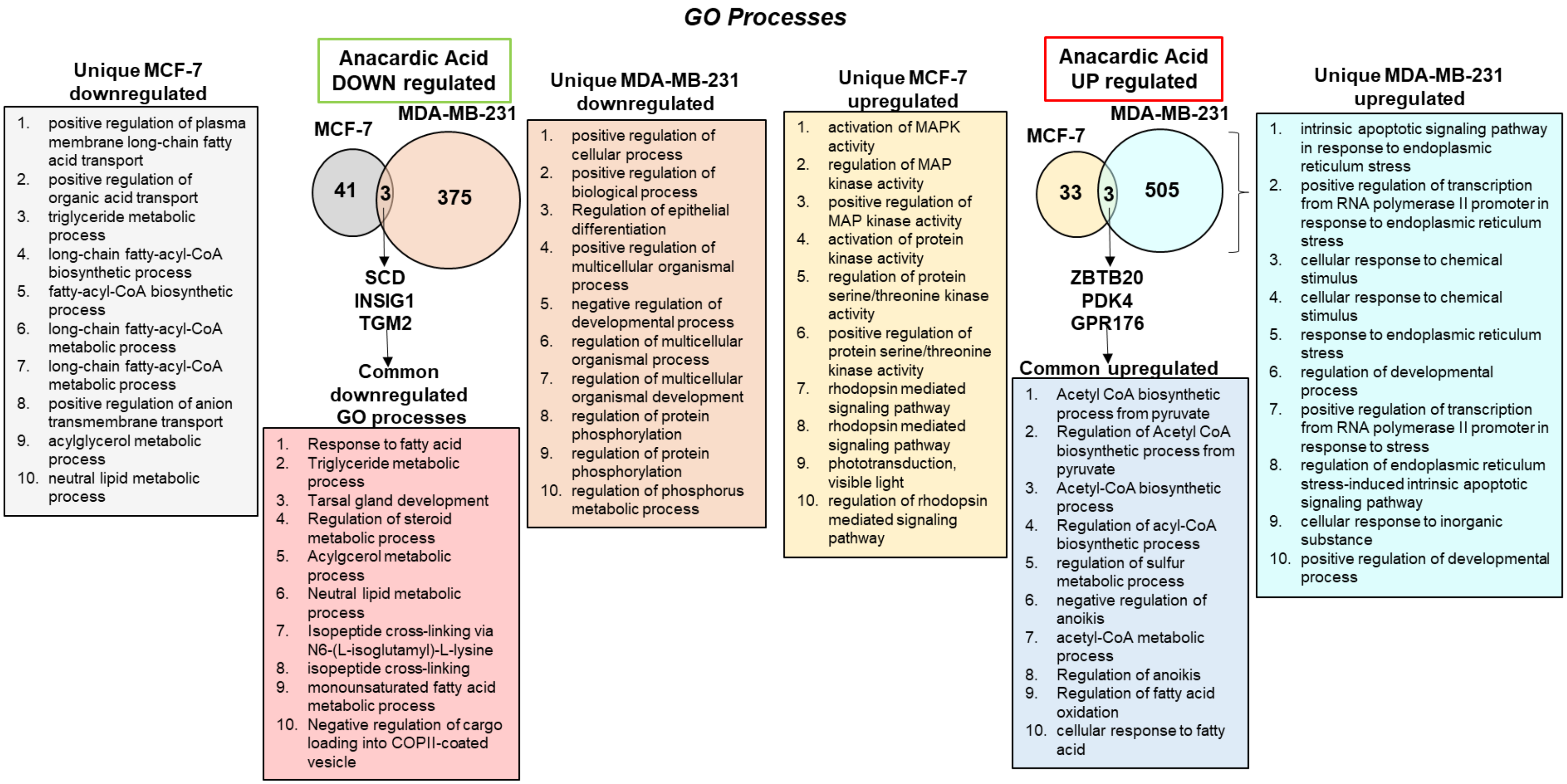

Supplementary Figure 1: Enrichment analysis of RNA seq data. Differentially expressed genes were identified in pairwise comparisons: MCF7 AnAc vs. MDA-MB-231 AnAc using the tuxedo suite of programs including cufflink-cuffdiff2. The Venn diagrams show the number of common and differentially expressed genes significantly downregulated (A) and upregulated (B). Pathway analysis was performed using GeneGo Pathways Software (MetaCore^{TM}). The GO Processes identified for each comparison are listed in the order provided by MetaCore^{TM} analysis.

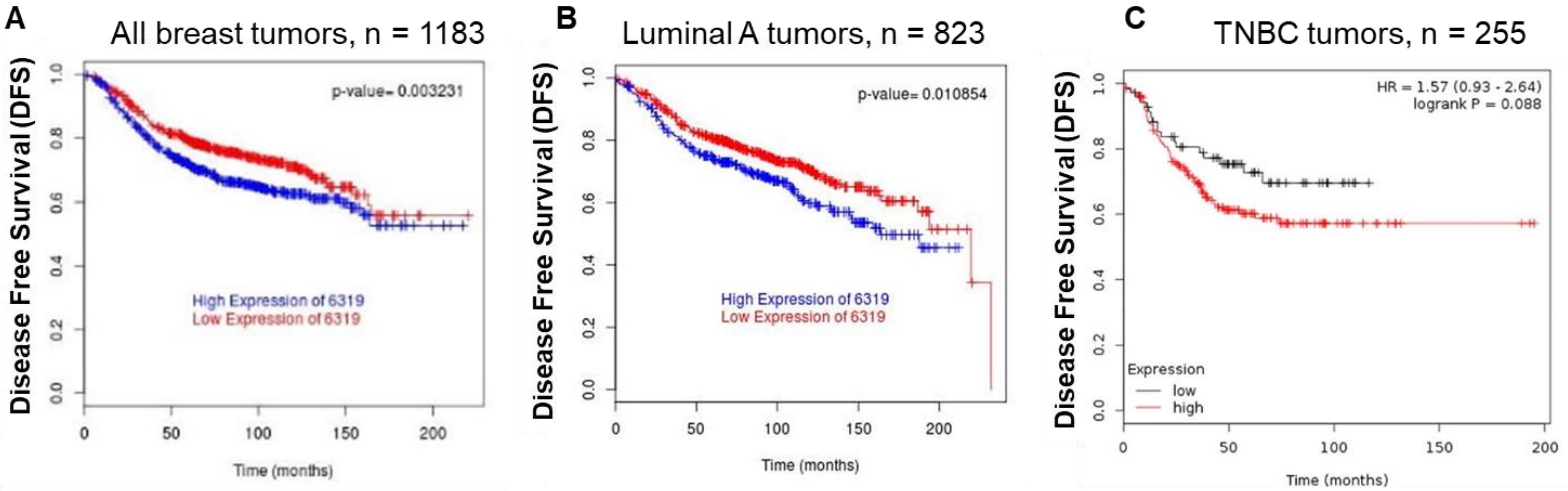

Supplementary Figure 2: Correlation of SCD transcript expression in human breast tumors with overall disease free survival (DFS). SCD transcript expression in all breast tumors (A) and luminal A subtype (B) from BreastMark http://glados.ucd.ie/BreastMark/index.html and TNBC (C) from KM Plotter http://kmplot.com.

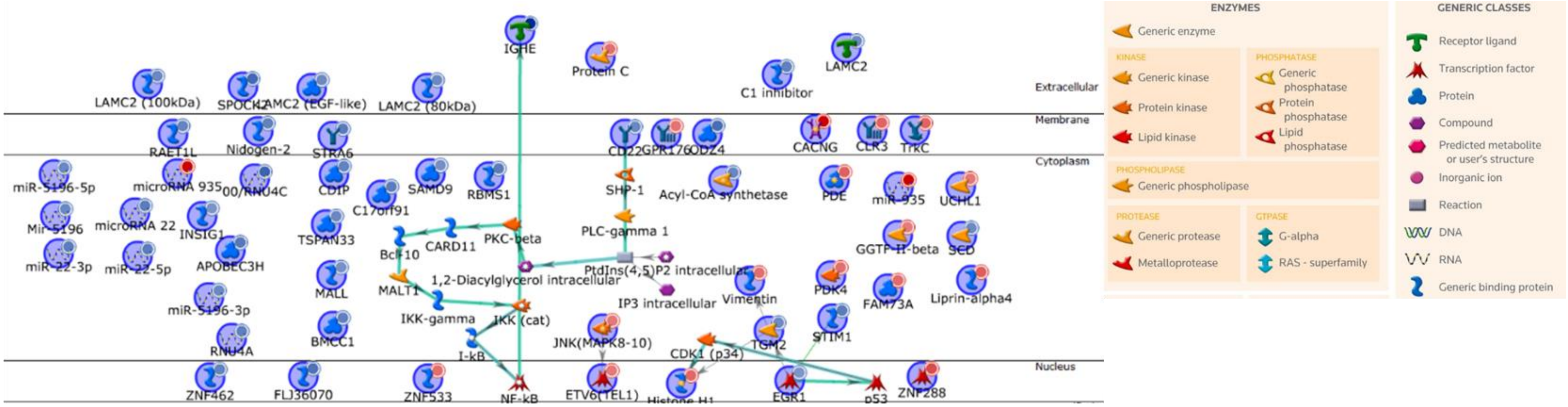

Supplementary Figure 3: MetaCore network analysis of DEG in MCF-7 cells treated with AnAc. MetaCore pathway enrichment analysis identified networks associated with gene expression changes that were uniquely regulated by AnAc versus EtOH in MCF-7 breast cancer cells. Shown are the results of Dijkstra's shortest paths algorithm calculating the shortest directed paths between the genes differentially expressed in MCF-7 cells treated with AnAc with two steps in the path. The lines are connections that have been documented in the literature with green lines indicating canonical pathways. All objects with the red circle are upregulated by AnAc and those with blue circles are downregulated by AnAc.

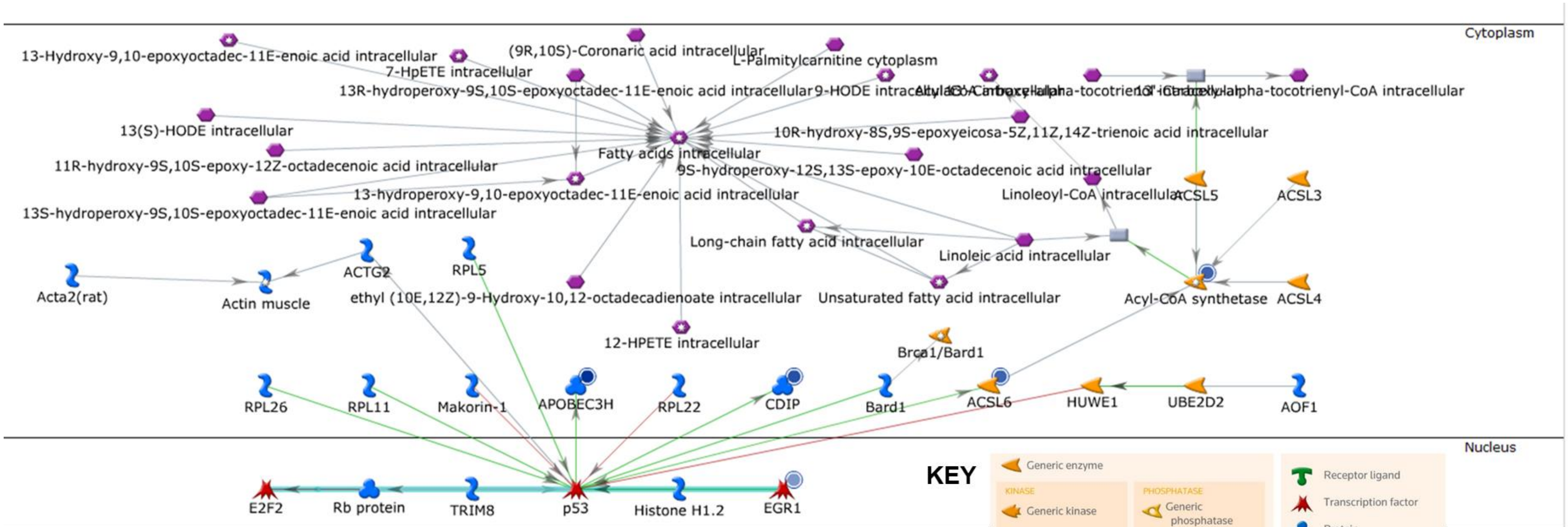

KEY

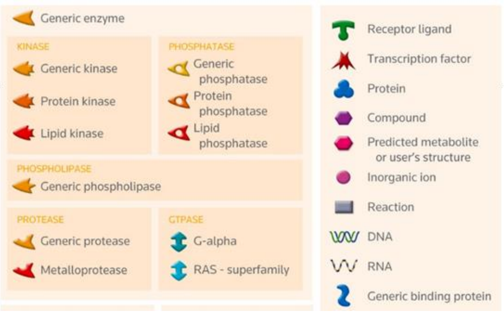

Supplementary Figure 4: The top network for AnAc-downregulated genes in MCF-7 cells generated by MetaCore: AcylCoA synthetase, CDIP, APOBEC3H, ACLS6, and EGR1: "positive regulation of plasma membrane long-chain fatty acid transport (11.5%), long-chain fatty-acyl-CoA biosynthetic process (19.2%), fatty-acyl-CoA biosynthetic process (19.2%), long-chain fatty-acyl-CoA metabolic process (19.2%), fatty-acyl-CoA metabolic process (19.2%). P value 1.57 e-13". The lines are connections that have been documented in the literature with green lines indicating canonical pathways. All objects with the blue circle are downregulated by AnAc.

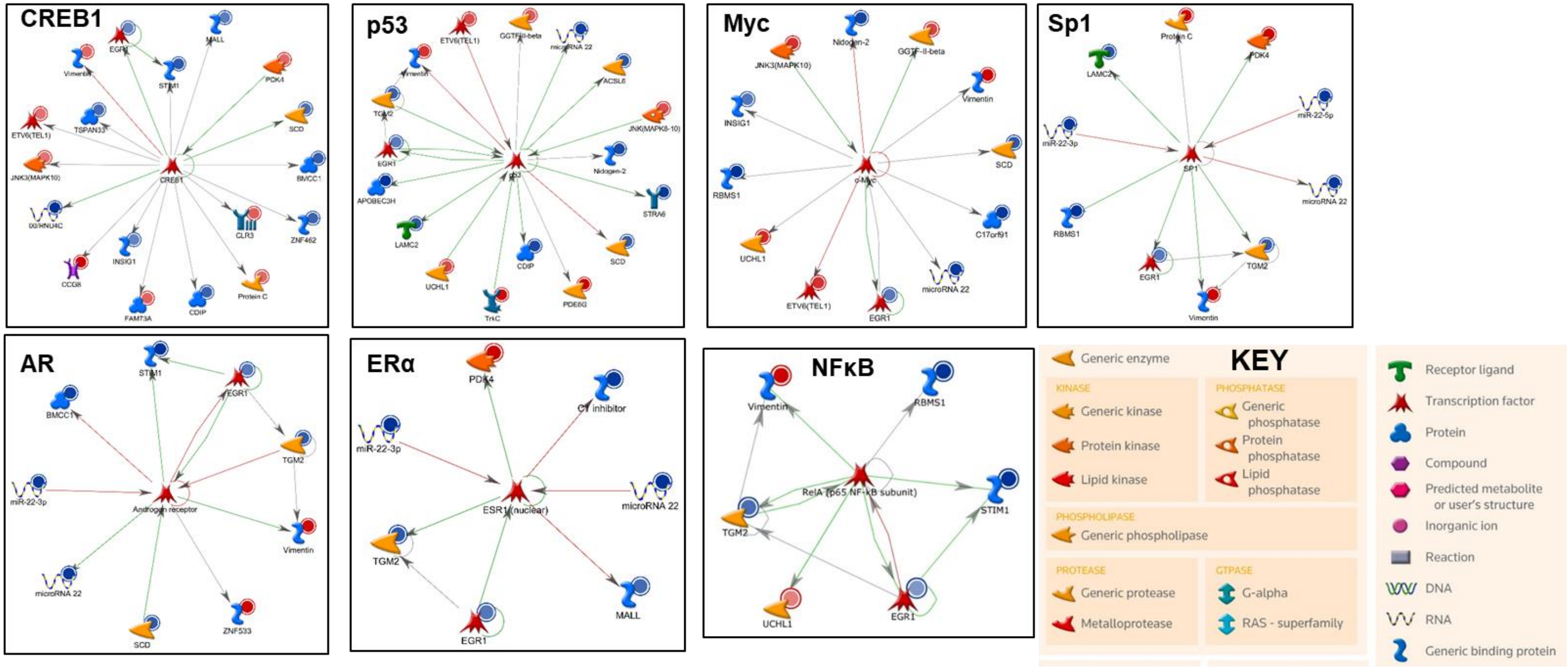

Supplementary Figure 5: Transcription factor regulation of DEGs in AnAc-treated MCF-7 cells generated by MetaCore™: MetaCore™ transcription factor network analysis identified 30 transcription factors in the DEGs in MCF-7 cells including CREB, p53, Myc, Sp1, AR, ESR1 (ERα), and RelA/NFκB.

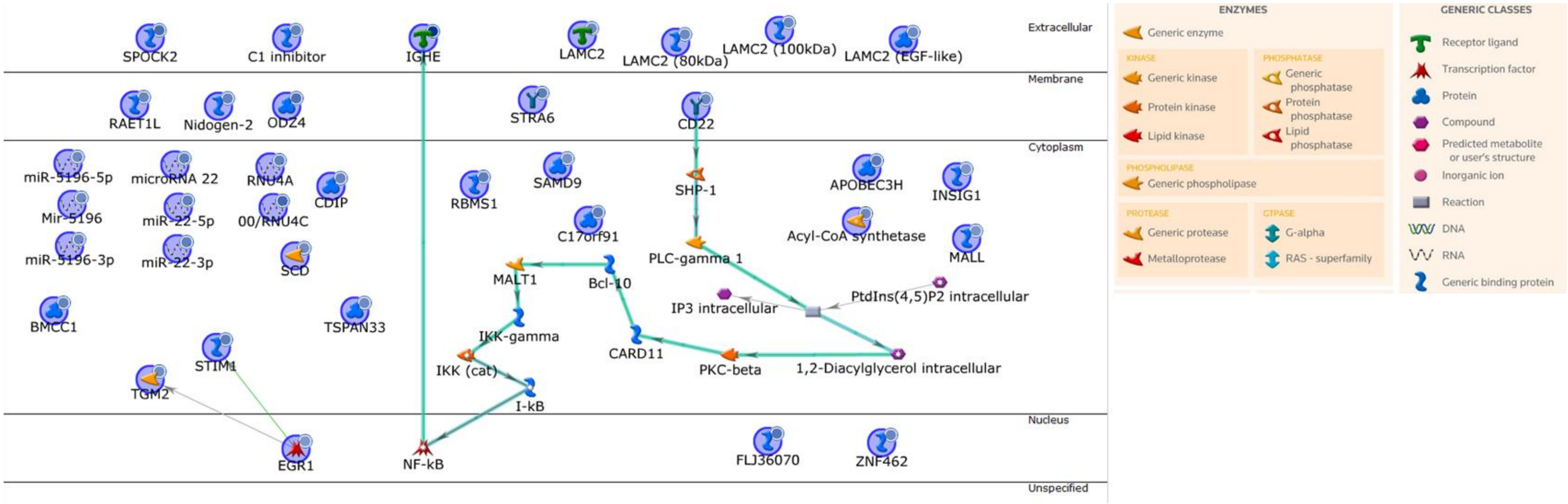

Supplementary Figure 6: Shortest paths with one step generated by MetaCore for genes downregulated by AnAc in MCF-7 cells. The lines are connections that have been documented in the literature with green lines indicating canonical pathways. All objects with the blue circle are downregulated by AnAc.

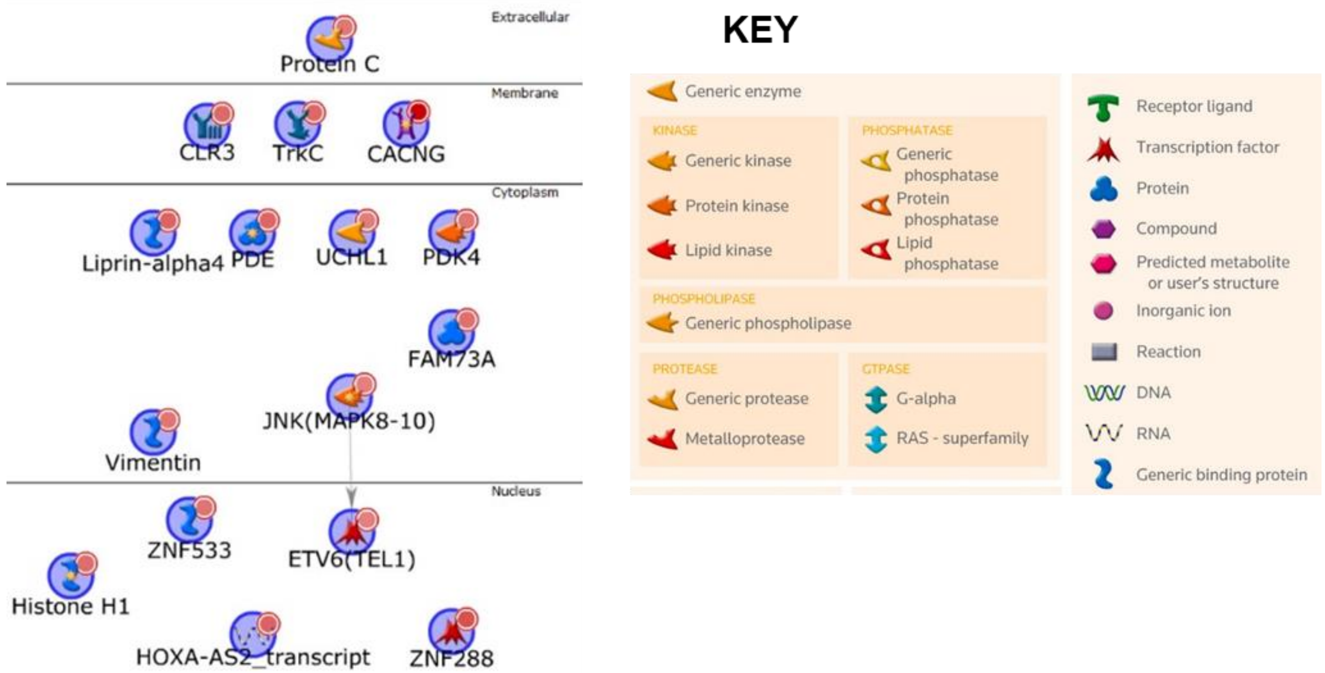

Supplementary Figure 7: Shown is the shortest paths analysis with 1 step generated by MetaCore for genes upregulated by AnAc in MCF-7 cells. The lines are connections that have been documented in the literature with green lines indicating canonical pathways. All objects with the red circle are upregulated by AnAc. The MetaCore legend is shown at the right.

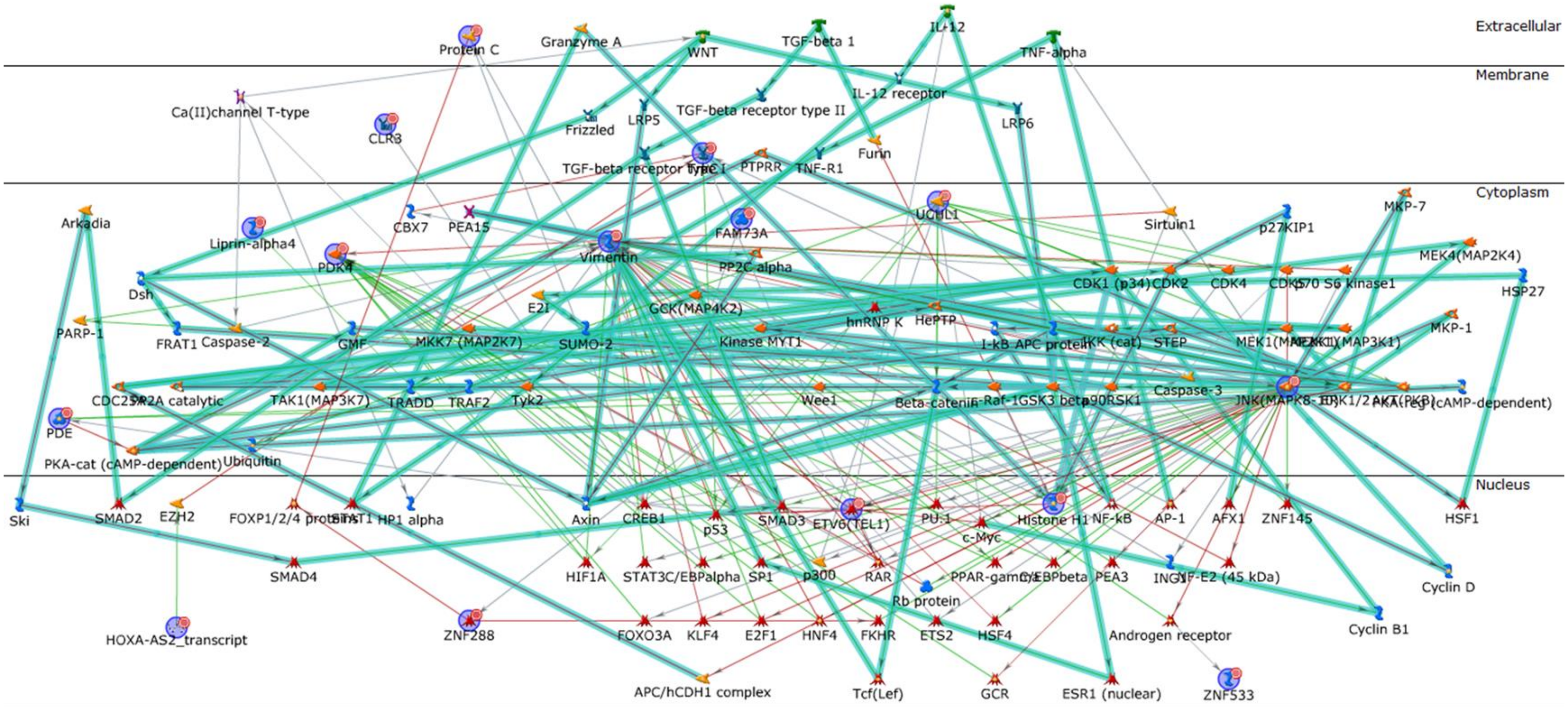

Supplementary Figure 8: Shown is the shortest paths analysis with 2 steps generated by MetaCore for genes upregulated by AnAc in MCF-7 cells. The lines are connections that have been documented in the literature with green lines indicating canonical pathways. All objects with the red circle are upregulated by AnAc. The network of up-regulated genes centers on *Vimentin, JNK(MAPK8-10), PDK4, UCHL1, ETV6(TEL1*). The MetaCore legend is shown in Supplementary Fig. 6.

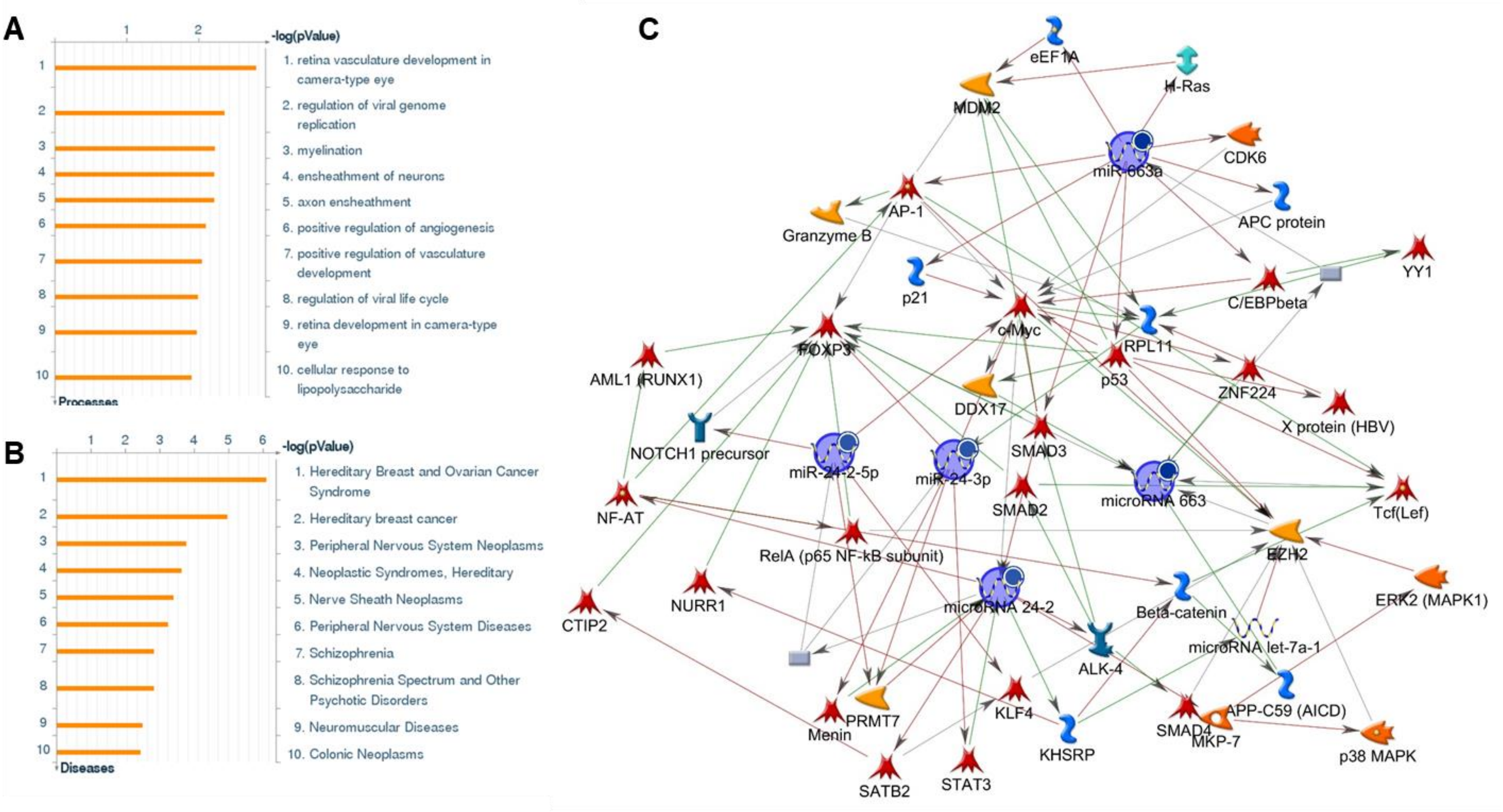

Supplementary Figure 9: These are Gene Ontology (GO) cellular processes (A) and diseases (B) and the Network (5 steps) identified by MetaCore by enrichment analysis of the lncRNAs regulated by AnAc in MDA-MB-231 cells (Table 9).

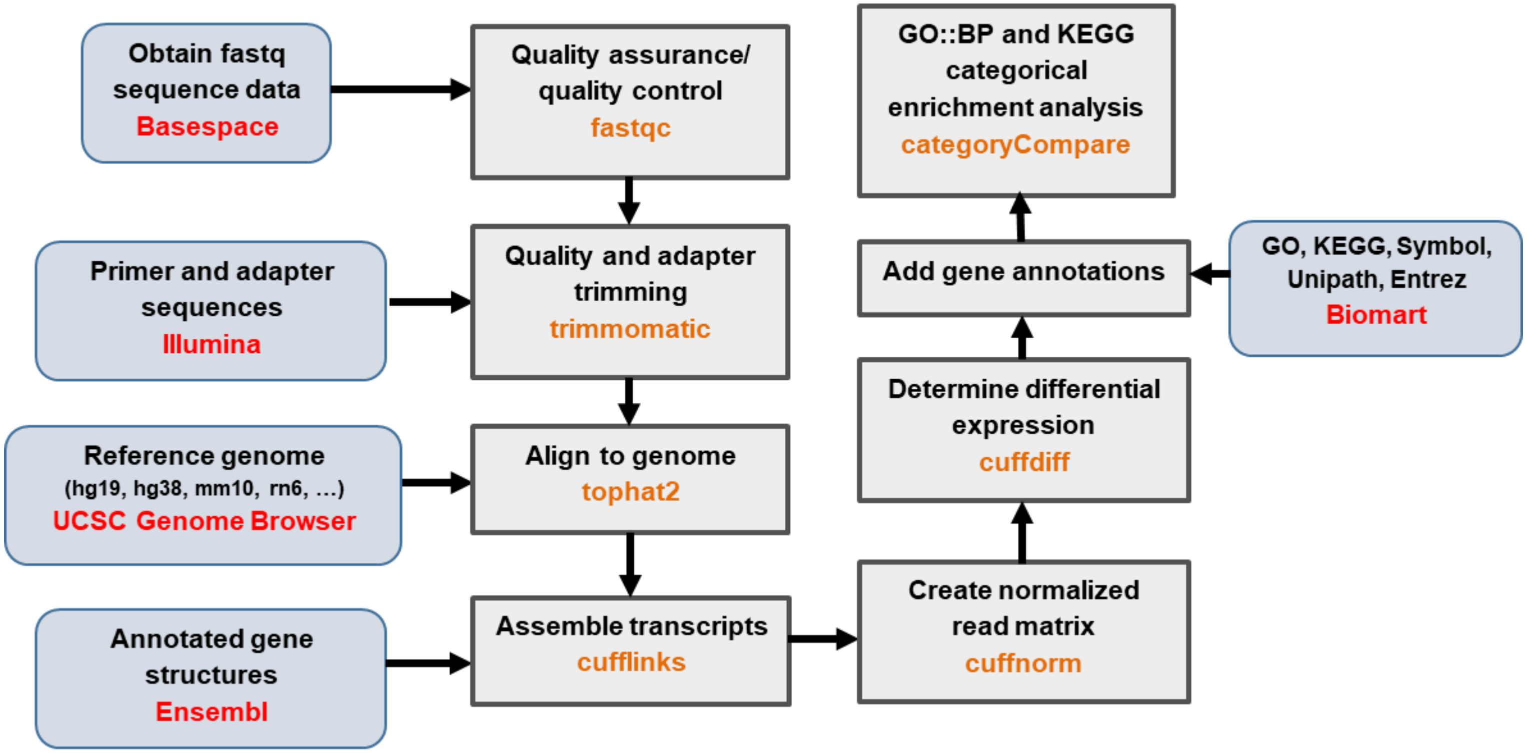

Supplementary Figure 10: Data analysis pipeline for differential expression analysis.

REFERENCES in Supplementary Tables.

1 Llaverias, G. *et al.* Role of Cholesterol in the Development and Progression of Breast Cancer. *The American Journal of Pathology* **178**, 402-412, doi:10.1016/j.ajpath.2010.11.005 (2011).

2 Liu, J. *et al.* Cholesterol-induced mammary tumorigenesis is enhanced by adiponectin deficiency: role of LDL receptor upregulation. *Oncotarget* **4**, 1804-1818, doi:10.18632/oncotarget.1364 (2013).

3 Simigdala, N. *et al.* Cholesterol biosynthesis pathway as a novel mechanism of resistance to estrogen deprivation in estrogen receptor-positive breast cancer. *Breast Cancer Research* **18**, 1-14, doi:10.1186/s13058-016-0713-5 (2016).

4 Yang, T. *et al.* Effects of Lovastatin on MDA-MB-231 Breast Cancer Cells: An Antibody Microarray Analysis. *J Cancer* **7**, 192-199, doi:10.7150/jca.13414 (2016).

5 Ampuja, M. *et al.* BMP4 inhibits the proliferation of breast cancer cells and induces an MMP-dependent migratory phenotype in MDA-MB-231 cells in 3D environment. *BMC Cancer* **13**, 429, doi:10.1186/1471-2407-13-429 (2013).

6 Frasor, J. *et al.* Profiling of estrogen up- and down-regulated gene expression in human breast cancer cells: insights into gene networks and pathways underlying estrogenic control of proliferation and cell phenotype. *Endocrinology* **144**, 4562-4574 (2003).

7 Krzewinski-Recchi, M.-A. *et al.* Evidence for splice transcript variants of TMEM165, a gene involved in CDG. *Biochimica et Biophysica Acta (BBA) - General Subjects* **1861**, 737-748, doi:10.1016/j.bbagen.2017.01.011 (2017).

8 Dulary, E., Potelle, S., Legrand, D. & Foulquier, F. TMEM165 deficiencies in Congenital Disorders of Glycosylation type II (CDG-II): Clues and evidences for roles of the protein in Golgi functions and ion homeostasis. *Tissue Cell*, doi:10.1016/j.tice.2016.06.006 (2017).

9 Wang, B. *et al.* Metastatic consequences of immune escape from NK cell cytotoxicity by human breast cancer stem cells. *Cancer Res.* **74**, 5746-5757, doi:10.1158/0008-5472.can-13-2563 (2014).

10 Roberti, M., Mordoh, J. & Levy, E. Biological role of NK cells and immunotherapeutic approaches in breast cancer. *Frontiers in Immunology* **3**, doi:10.3389/fimmu.2012.00375 (2012).

11 Hilakivi-Clarke, L. *et al.* Effects of In Utero Exposure to Ethinyl Estradiol on Tamoxifen Resistance and Breast Cancer Recurrence in a Preclinical Model. *JNCI: Journal of the National Cancer Institute* **109**, 1-11, doi:10.1093/jnci/djw188 (2017).

12 Amin, P. J. & Shankar, B. S. Sulforaphane induces ROS mediated induction of NKG2D ligands in human cancer cell lines and enhances susceptibility to NK cell mediated lysis. *Life Sci.* **126**, 19-27, doi:10.1016/j.lfs.2015.01.026 (2015).

13 Girasole, G., Passeri, G., Jilka, R. L. & Manolagas, S. C. Interleukin-11: a new cytokine critical for osteoclast development. *J. Clin. Invest.* **93**, 1516-1524, doi:10.1172/jci117130 (1994).

14 Ren, L., Wang, X., Dong, Z., Liu, J. & Zhang, S. Bone metastasis from breast cancer involves elevated IL-11 expression and the gp130/STAT3 pathway. *Med. Oncol.* **30**, 634, doi:10.1007/s12032-013-0634-4 (2013).

15 Lim, J. H. Inhibition of the Interleukin-11-STAT3 Axis Attenuates Hypoxia-Induced Migration and Invasion in MDA-MB-231 Breast Cancer Cells. *The Korean journal of physiology & pharmacology : official journal of the Korean Physiological Society and the Korean Society of Pharmacology* **18**, 391-396, doi:10.4196/kjpp.2014.18.5.391 (2014).

16 Schulz, R. & Schlüter, K.-D. PCSK9 targets important for lipid metabolism. *Clinical Research in Cardiology Supplements*, 1-10, doi:10.1007/s11789-017-0085-0 (2017).
17 Krysa, J. A., Ooi, T. C., Proctor, S. D. & Vine, D. F. Nutritional and Lipid Modulation of PCSK9: Effects on Cardiometabolic Risk Factors. *The Journal of Nutrition*, doi:10.3945/jn.116.235069 (2017).
18 He, M. *et al.* Pro-inflammation NF-kappaB signaling triggers a positive feedback via enhancing cholesterol accumulation in liver cancer cells. *J. Exp. Clin. Cancer Res.* **36**, 15, doi:10.1186/s13046-017-0490-8 (2017).
19 Cruz, P., Mo, H., McConathy, W., Sabnis, N. & Lacko, A. The role of cholesterol metabolism and cholesterol transport in carcinogenesis: a review of scientific findings, relevant to future cancer therapeutics. *Frontiers in Pharmacology* **4**, doi:10.3389/fphar.2013.00119 (2013).
20 Beloribi-Djefaflia, S., Vasseur, S. & Guillaumond, F. Lipid metabolic reprogramming in cancer cells. *Oncogenesis* **5**, e189, doi:10.1038/oncsis.2015.49 (2016).
21 Ernkvist, M. *et al.* The Amot/Patj/Syx signaling complex spatially controls RhoA GTPase activity in migrating endothelial cells. *Blood* **113**, 244-253, doi:10.1182/blood-2008-04-153874 (2009).
22 Mojallal, M. *et al.* AmotL2 disrupts apical-basal cell polarity and promotes tumour invasion. *Nat Commun* **5**, 4557, doi:10.1038/ncomms5557 (2014).
23 Ochsner, S. A. *et al.* Transcriptomine, a web resource for nuclear receptor signaling transcriptomes. *Physiol Genomics* **44**, 853-863, doi:10.1152/physiolgenomics.00033.2012 (2012).
24 Ozturk, S. *et al.* SDPR functions as a metastasis suppressor in breast cancer by promoting apoptosis. *Proceedings of the National Academy of Sciences* **113**, 638-643, doi:10.1073/pnas.1514663113 (2016).
25 Tian, Y. *et al.* Serum deprivation response inhibits breast cancer progression by blocking transforming growth factor-β signaling. *Cancer Sci.* **107**, 274-280, doi:10.1111/cas.12879 (2016).
26 Johnstone, R. W., Frew, A. J. & Smyth, M. J. The TRAIL apoptotic pathway in cancer onset, progression and therapy. *Nat Rev Cancer* **8**, 782-798 (2008).
27 Yoshida, T., Zhang, Y., Rivera Rosado, L. A. & Zhang, B. Repeated Treatment with Subtoxic Doses of TRAIL Induces Resistance to Apoptosis through Its Death Receptors in MDA-MB-231 Breast Cancer Cells. *Mol. Cancer Res.* **7**, 1835 (2009).
28 Huo, D. *et al.* Genome-wide association studies in women of African ancestry identified 3q26.21 as a novel susceptibility locus for oestrogen receptor negative breast cancer. *Hum. Mol. Genet.*, doi:10.1093/hmg/ddw305 (2016).
29 Afratis, N. A. *et al.* Syndecans – key regulators of cell signaling and biological functions. *The FEBS Journal* **284**, 27-41, doi:10.1111/febs.13940 (2017).
30 Tinholt, M. *et al.* Syndecan-3 and TFPI colocalize on the surface of endothelial-, smooth muscle-, and cancer cells. *PLoS One* **10**, e0117404, doi:10.1371/journal.pone.0117404 (2015).
31 Wu, Z. S. *et al.* Prognostic significance of the expression of GFRalpha1, GFRalpha3 and syndecan-3, proteins binding ARTEMIN, in mammary carcinoma. *BMC Cancer* **13**, 34, doi:10.1186/1471-2407-13-34 (2013).
32 Mercier, S. *et al.* Mutations in FAM111B Cause Hereditary Fibrosing Poikiloderma with Tendon Contracture, Myopathy, and Pulmonary Fibrosis. *The American Journal of Human Genetics* **93**, 1100-1107, doi:10.1016/j.ajhg.2013.10.013 (2013).

33 Yue, W. *et al.* Transcriptomic analysis of pancreatic cancer cells in response to metformin and aspirin: an implication of synergy. *Scientific reports* **5**, 13390, doi:10.1038/srep13390 (2015).

34 Grazzini, R. *et al.* Inhibition of lipoxygenase and prostaglandin endoperoxide synthase by anacardic acids. *Biochem. Biophys. Res. Commun.* **176**, 775-780 (1991).

35 Nordgard, S. H. *et al.* Genome-wide analysis identifies 16q deletion associated with survival, molecular subtypes, mRNA expression, and germline haplotypes in breast cancer patients. *Genes. Chromosomes Cancer* **47**, 680-696, doi:10.1002/gcc.20569 (2008).

36 Makanji, Y. *et al.* Inhibin at 90: from discovery to clinical application, a historical review. *Endocr. Rev.* **35**, 747-794, doi:10.1210/er.2014-1003 (2014).

37 Eriksson, N. *et al.* Genetic variants associated with breast size also influence breast cancer risk. *BMC medical genetics* **13**, 53, doi:10.1186/1471-2350-13-53 (2012).

38 Wilson, C. Reproductive hormones in breast cancer bone metastasis: The role of inhibins. *Journal of Bone Oncology* **5**, 139-142, doi:10.1016/j.jbo.2016.03.005 (2016).

39 Brunetto, E. *et al.* CDC25A protein stability represents a previously unrecognized target of HER2 signaling in human breast cancer: implication for a potential clinical relevance in trastuzumab treatment. *Neoplasia* **15**, 579-590 (2013).

40 Sur, S. & Agrawal, D. K. Phosphatases and kinases regulating CDC25 activity in the cell cycle: clinical implications of CDC25 overexpression and potential treatment strategies. *Mol. Cell. Biochem.* **416**, 33-46, doi:10.1007/s11010-016-2693-2 (2016).

41 Zhang, S. *et al.* Manic Fringe Promotes a Claudin-Low Breast Cancer Phenotype through Notch-Mediated PIK3CG Induction. *Cancer Res.* **75**, 1936 (2015).

42 Xu, K. *et al.* Lunatic Fringe Deficiency Cooperates with the Met/Caveolin Gene Amplicon to Induce Basal-like Breast Cancer. *Cancer Cell* **21**, 626-641, doi:10.1016/j.ccr.2012.03.041 (2012).

43 Zhang, S., Chung, W. C. & Xu, K. Lunatic Fringe is a potent tumor suppressor in Kras-initiated pancreatic cancer. *Oncogene* **35**, 2485-2495, doi:10.1038/onc.2015.306 (2016).

44 Orzechowska, M., Jędroszka, D. & Bednarek, A. K. Common profiles of Notch signaling differentiate disease-free survival in luminal type A and triple negative breast cancer. *Oncotarget* **8** (2016).

45 Udani, M. *et al.* Basal cell adhesion molecule/lutheran protein. The receptor critical for sickle cell adhesion to laminin. *J. Clin. Invest.* **101**, 2550-2558, doi:10.1172/jci1204 (1998).

46 Rust, S. *et al.* Combining phenotypic and proteomic approaches to identify membrane targets in a 'triple negative' breast cancer cell type. *Mol Cancer* **12**, 11, doi:10.1186/1476-4598-12-11 (2013).

47 Li, L. *et al.* Serum cytokine profile in patients with breast cancer. *Cytokine* **89**, 173-178, doi:10.1016/j.cyto.2015.12.017 (2017).

48 Enomoto-Okawa, Y. *et al.* An Anti-Human Lutheran Glycoprotein Phage Antibody Inhibits Cell Migration on Laminin-511: Epitope Mapping of the Antibody. *PLOS ONE* **12**, e0167860, doi:10.1371/journal.pone.0167860 (2017).

49 Ariazi, E. A. *et al.* Estrogen induces apoptosis in estrogen deprivation-resistant breast cancer through stress responses as identified by global gene expression across time. *Proceedings of the National Academy of Sciences* **108**, 18879-18886, doi:10.1073/pnas.1115188108 (2011).

50 Cai, S. *et al.* CD98 modulates integrin beta1 function in polarized epithelial cells. *J. Cell Sci.* **118**, 889-899, doi:10.1242/jcs.01674 (2005).
51 Liu, C.-L. *et al.* Genome-wide analysis of tunicamycin-induced endoplasmic reticulum stress response and the protective effect of endoplasmic reticulum inhibitors in neonatal rat cardiomyocytes. *Mol. Cell. Biochem.* **413**, 57-67, doi:10.1007/s11010-015-2639-0 (2016).
52 El Ansari, R. *et al.* The multifunctional solute carrier 3A2 (SLC3A2) confers a poor prognosis in the highly proliferative breast cancer subtypes. *Br. J. Cancer*, doi:10.1038/s41416-018-0038-5 (2018).
53 Park, S., Li, C., Zhao, H., Darzynkiewicz, Z. & Xu, D. Gene 33/Mig6 inhibits hexavalent chromium-induced DNA damage and cell transformation in human lung epithelial cells. *Oncotarget* **7**, 8916-8930, doi:10.18632/oncotarget.6866 (2016).
54 Kim, T. H., Yoo, J. Y. & Jeong, J. W. Mig-6 Mouse Model of Endometrial Cancer. *Adv. Exp. Med. Biol.* **943**, 243-259, doi:10.1007/978-3-319-43139-0_8 (2017).
55 Li, Z. *et al.* Overexpression and knockout of miR-126 both promote leukemogenesis. *Blood* **126**, 2005 (2015).
56 van Laar, T., van der Eb, A. J. & Terleth, C. A role for Rad23 proteins in 26S proteasome-dependent protein degradation? *Mutation Research/Fundamental and Molecular Mechanisms of Mutagenesis* **499**, 53-61, doi:10.1016/S0027-5107(01)00291-3 (2002).
57 Chen, E. *et al.* PLIN2 is a Key Regulator of the Unfolded Protein Response and Endoplasmic Reticulum Stress Resolution in Pancreatic β Cells. *Scientific reports* **7**, 40855, doi:10.1038/srep40855 (2017).
58 Conte, M., Franceschi, C., Sandri, M. & Salvioli, S. Perilipin 2 and Age-Related Metabolic Diseases: A New Perspective. *Trends in Endocrinology & Metabolism* **27**, 893-903, doi:10.1016/j.tem.2016.09.001 (2016).
59 Bosma, M. *et al.* Perilipin 2 Improves Insulin Sensitivity in Skeletal Muscle Despite Elevated Intramuscular Lipid Levels. *Diabetes* **61**, 2679 (2012).
60 Moleirinho, A. *et al.* Gains, Losses and Changes of Function after Gene Duplication: Study of the Metallothionein Family. *PLOS ONE* **6**, e18487, doi:10.1371/journal.pone.0018487 (2011).
61 Zhou, S. *et al.* Metallothionein prevents intermittent hypoxia-induced cardiac endoplasmic reticulum stress and cell death likely via activation of Akt signaling pathway in mice. *Toxicol. Lett.* **227**, 113-123, doi:10.1016/j.toxlet.2014.03.011 (2014).
62 Shajahan-Haq, A. *et al.* MYC regulates the unfolded protein response and glucose and glutamine uptake in endocrine resistant breast cancer. *Molecular Cancer* **13**, 239, doi:10.1186/1476-4598-13-239 (2014).
63 Hollander, M. C., Zhan, Q., Bae, I. & Fornace, A. J., Jr. Mammalian GADD34, an apoptosis- and DNA damage-inducible gene. *J. Biol. Chem.* **272**, 13731-13737 (1997).
64 Oyadomari, S. & Mori, M. Roles of CHOP//GADD153 in endoplasmic reticulum stress. *Cell Death Differ.* **11**, 381-389 (2003).
65 Tabas, I. & Ron, D. Integrating the mechanisms of apoptosis induced by endoplasmic reticulum stress. *Nat Cell Biol* **13**, 184-190 (2011).
66 Faltermann, S., Grundler, V., Gademann, K., Pernthaler, J. & Fent, K. Comparative effects of nodularin and microcystin-LR in zebrafish: 2. Uptake and molecular effects in eleuthero-embryos and adult liver with focus on endoplasmic reticulum stress. *Aquat. Toxicol.* **171**, 77-87, doi:https://doi.org/10.1016/j.aquatox.2015.12.001 (2016).

67 Frakes, A. E. & Dillin, A. The UPR^{ER}: Sensor and Coordinator of Organismal Homeostasis. *Mol. Cell* **66**, 761-771, doi:10.1016/j.molcel.2017.05.031 (2017).

68 Ohoka, N., Yoshii, S., Hattori, T., Onozaki, K. & Hayashi, H. TRB3, a novel ER stress-inducible gene, is induced via ATF4-CHOP pathway and is involved in cell death. *EMBO J.* **24**, 1243-1255, doi:10.1038/sj.emboj.7600596 (2005).

69 Izrailit, J., Jaiswal, A., Zheng, W., Moran, M. F. & Reedijk, M. Cellular stress induces TRB3/USP9x-dependent Notch activation in cancer. *Oncogene* **36**, 1048-1057, doi:10.1038/onc.2016.276 (2017).

70 Zhou, Y. *et al.* Quinocetone triggered ER stress-induced autophagy via ATF6/DAPK1-modulated mAtg9a trafficking. *Cell Biol. Toxicol.* **32**, 141-152, doi:10.1007/s10565-016-9323-3 (2016).

71 Li, Z. *et al.* LPA rescues ER stress-associated apoptosis in hypoxia and serum deprivation-stimulated mesenchymal stem cells. *J. Cell. Biochem.* **111**, 811-820, doi:10.1002/jcb.22731 (2010).

72 Li, B. *et al.* Differences in endoplasmic reticulum stress signalling kinetics determine cell survival outcome through activation of MKP-1. *Cell. Signal.* **23**, 35-45, doi:https://doi.org/10.1016/j.cellsig.2010.07.019 (2011).

73 Galluzzi, L. *et al.* Induction of endoplasmic reticulum stress response by the indole-3-carbinol cyclic tetrameric derivative CTet in human breast cancer cell lines. *PLoS One* **7**, e43249, doi:10.1371/journal.pone.0043249 (2012).

74 Tan, H. K., Muhammad, T. S. T. & Tan, M. L. 14-Deoxy-11,12-didehydroandrographolide induces DDIT3-dependent endoplasmic reticulum stress-mediated autophagy in T-47D breast carcinoma cells. *Toxicol. Appl. Pharmacol.* **300**, 55-69, doi:https://doi.org/10.1016/j.taap.2016.03.017 (2016).

75 Rabenau, K. E. *et al.* DEGA//AMIGO-2, a leucine-rich repeat family member, differentially expressed in human gastric adenocarcinoma: effects on ploidy, chromosomal stability, cell adhesion//migration and tumorigenicity. *Oncogene* **23**, 5056-5067 (2004).

76 Park, H. *et al.* AMIGO2, a novel membrane anchor of PDK1, controls cell survival and angiogenesis via Akt activation. *J. Cell Biol.* **211**, 619-637, doi:10.1083/jcb.201503113 (2015).

77 Mathe, A. *et al.* DNA methylation profile of triple negative breast cancer-specific genes comparing lymph node positive patients to lymph node negative patients. *Scientific reports* **6**, 33435, doi:10.1038/srep33435 (2016).

78 Gerhauser, C. in *Natural Products in Cancer Prevention and Therapy* Vol. 329 *Topics in Current Chemistry* (eds John M. Pezzuto & Nanjoo Suh) Ch. 360, 73-132 (Springer Berlin Heidelberg, 2013).

79 Yang, F. *et al.* Epigenetic modifications of interleukin-6 in synovial fibroblasts from osteoarthritis patients. *Scientific reports* **7**, 43592, doi:10.1038/srep43592 (2017).

80 Park, S. H. *et al.* Two in-and-out modulation strategies for endoplasmic reticulum stress-linked gene expression of pro-apoptotic macrophage-inhibitory cytokine 1. *J. Biol. Chem.* **287**, 19841-19855, doi:10.1074/jbc.M111.330639 (2012).

81 Montero, R. *et al.* GDF-15 Is Elevated in Children with Mitochondrial Diseases and Is Induced by Mitochondrial Dysfunction. *PLOS ONE* **11**, e0148709, doi:10.1371/journal.pone.0148709 (2016).

82 Wang, X., Baek, S. J. & Eling, T. E. The diverse roles of nonsteroidal anti-inflammatory drug activated gene (NAG-1/GDF15) in cancer. *Biochem. Pharmacol.* **85**, 597-606, doi:10.1016/j.bcp.2012.11.025 (2013).

83 Yang, M. H., Kim, J., Khan, I. A., Walker, L. A. & Khan, S. I. Nonsteroidal anti-inflammatory drug activated gene-1 (NAG-1) modulators from natural products as anti-cancer agents. *Life Sci.* **100**, 75-84, doi:10.1016/j.lfs.2014.01.075 (2014).

84 Colacino, J. A., McDermott, S. P., Sartor, M. A., Wicha, M. S. & Rozek, L. S. Transcriptomic profiling of curcumin-treated human breast stem cells identifies a role for stearoyl-coa desaturase in breast cancer prevention. *Breast Cancer Res. Treat.*, doi:10.1007/s10549-016-3854-4 (2016).
85 Cheng, H., Wang, S. & Feng, R. STIM1 plays an important role in TGF-beta-induced suppression of breast cancer cell proliferation. *Oncotarget* **7**, 16866-16878, doi:10.18632/oncotarget.7619 (2016).
86 Brown, L. *et al.* CDIP, a novel pro-apoptotic gene, regulates TNFalpha-mediated apoptosis in a p53-dependent manner. *EMBO J.* **26**, 3410-3422, doi:10.1038/sj.emboj.7601779 (2007).
87 Einbond, L. S. *et al.* Gene expression analysis of the mechanisms whereby black cohosh inhibits human breast cancer cell growth. *Anticancer Res.* **27**, 697-712 (2007).
88 Tani, H., Onuma, Y., Ito, Y. & Torimura, M. Long Non-Coding RNAs as Surrogate Indicators for Chemical Stress Responses in Human-Induced Pluripotent Stem Cells. *PLoS ONE* **9**, e106282, doi:10.1371/journal.pone.0106282 (2014).
89 Linher-Melville, K. *et al.* Establishing a relationship between prolactin and altered fatty acid β-Oxidation via carnitine palmitoyl transferase 1 in breast cancer cells. *BMC Cancer* **11**, 1-15, doi:10.1186/1471-2407-11-56 (2011).
